# Effectiveness of behavioural interventions with motivational interviewing on physical activity outcomes in adults: systematic review and meta-analysis

**DOI:** 10.1136/bmj-2023-078713

**Published:** 2024-07-10

**Authors:** SuFen Zhu, Deepra Sinha, Megan Kirk, Moscho Michalopoulou, Anisa Hajizadeh, Gina Wren, Paul Doody, Lucy Mackillop, Ralph Smith, Susan A Jebb, Nerys M Astbury

**Affiliations:** 1 Nuffield Department of Primary Care Health Sciences, University of Oxford, Oxford, UK; 2 St Hugh’s College, University of Oxford, Oxford, UK; 3 Nuffield Department of Women’s and Reproductive Health, University of Oxford, Oxford, UK; 4 Sport and Exercise Medicine Department, Nuffield Orthopaedic Centre Oxford University Hospitals NHS Foundation Trust, Oxford, UK

## Abstract

**Objective:**

To evaluate the effectiveness of behavioural interventions that include motivational interviewing on physical activity outcomes in adults.

**Design:**

Systematic review and meta-analysis.

**Study selection:**

A search of seven databases for randomised controlled trials published from inception to 1 March 2023 comparing a behavioural intervention including motivational interviewing with a comparator without motivational interviewing on physical activity outcomes in adults. Outcomes of interest were differences in change in quantitative measures of total physical activity, moderate to vigorous physical activity (MVPA), and sedentary time.

**Data extraction and synthesis:**

Two reviewers extracted data and assessed risk of bias. Population characteristics, intervention components, comparison groups, and outcomes of studies were summarised. For overall main effects, random effects meta-analyses were used to report standardised mean differences (SMDs) and 95% confidence intervals (CIs). Differential effects based on duration of follow-up, comparator type, intervention duration, and disease or health condition of participants were also examined.

**Results:**

129 papers reporting 97 randomised controlled trials totalling 27 811 participants and 105 comparisons were included. Interventions including motivational interviewing were superior to comparators for increases in total physical activity (SMD 0.45, 95% CI 0.33 to 0.65, equivalent to 1323 extra steps/day; low certainty evidence) and MVPA (0.45, 0.19 to 0.71, equivalent to 95 extra min/week; very low certainty evidence) and for reductions in sedentary time (−0.58, −1.03 to −0.14, equivalent to −51 min/day; very low certainty evidence). Evidence for a difference in any outcome compared with comparators of similar intensity was lacking. The magnitude of effect diminished over time, and evidence of an effect of motivational interviewing beyond one year was lacking. Most interventions involved patients with a specific health condition, and evidence of an effect of motivational interviewing to increase MVPA or decrease sedentary time was lacking in general population samples.

**Conclusions:**

Certainty of the evidence using motivational interviewing as part of complex behavioural interventions for promoting total physical activity in adults was low, and for MVPA and sedentary time was very low. The totality of evidence suggests that although interventions with motivational interviewing increase physical activity and decrease sedentary behaviour, no difference was found in studies where the effect of motivational interviewing could be isolated. Effectiveness waned over time, with no evidence of a benefit of motivational interviewing to increase physical activity beyond one year.

**Systematic review registration:**

PROSPERO CRD42020219881.

## Introduction

Physical inactivity, or the failure to meet physical activity recommendations, is one of the leading risk factors for non-communicable diseases,^1^ and it is responsible for an estimated 9% of premature deaths worldwide.^2^ The benefits on health of being physically active are dose dependent, so most people—including those who currently achieve physical activity recommendations—are likely to benefit from being more physically active.^3^

Guidelines from the World Health Organization recommend that adults (aged 18-64 years) should engage in a minimum of 150-300 minutes of moderate intensity, or 75 minutes of vigorous intensity, physical activity each week, combined with strength training activities to develop or maintain strength in major muscle groups, as well as a reduction in sedentary time.^4^ Despite longstanding policy initiatives, however, one in three women and one in four men do not meet the levels of physical activity set out in the guidelines.^5^ ^6^ A systematic review found that individual level interventions to promote physical activity that provided professional advice and guidance with continued support can encourage people to be more physically active in the short to medium term.^7^ More research is, however, needed to establish which behaviour change techniques are most effective in the long term.^7^

Motivational interviewing is a communication technique commonly used in multicomponent, complex interventions to elicit behavioural change.^8^ It is a patient centred counselling style that helps patients change their problematic behaviours by exploring and resolving their ambivalence towards behavioural change in a non-confrontational style.^9^ Motivational interviewing empowers patients to increase their autonomous motivation, such that change arises from within the individual rather than being imposed by others,^10^ ^11^ and it has been used successfully in people who smoke, have addiction problems, or have an eating disorder, and in diabetes management.^12^ ^13^ ^14^ Therefore motivational interviewing may be a useful technique to help people achieve physical activity guidelines, since interventions that include motivational interviewing could feasibly be delivered at scale by healthcare professionals who have regular contact with people.

Previous systematic reviews and meta-analyses have reported that interventions with motivational interviewing led to a small but significant increase in physical activity in the short term in patients with specific health conditions.^15^ ^16^ ^17^ However, less consideration has been given to longer term effects and the effects in general population samples. Motivational interviewing requires the people who deliver the interventions to undergo specialist training and continued professional development to learn and develop the skills required to enhance motivation towards behaviour change. Interventions that include motivational interviewing therefore require extended intervention contact time and sessions, resulting in additional time and financial resources for delivery. As such it is important to determine the effectiveness of interventions with motivational interviewing and to examine the durability of the effect beyond the active intervention period.

We systematically reviewed the evidence from randomised controlled trials for behavioural interventions that included motivational interviewing for the promotion of physical activity in adults. Additionally, we examined the effect of treatment duration, durability of any effect, and the effectiveness in groups selected on the basis of pre-existing disease or health conditions, or in the general population who were not specifically selected because of their health condition or disease status.

## Methods

This systematic review and meta-analysis was performed in accordance with the PRISMA (Preferred Reporting Items for Systematic reviews and Meta-Analyses) guidelines.^18^ A protocol was developed and prospectively registered with PROSPERO and is available at https://www.crd.york.ac.uk/prospero/display_record.php?RecordID=219881.

### Eligibility criteria

Eligible criteria were randomised controlled trials, including cluster randomised trials, in adults (≥18 years) that compared interventions comprising motivational interviewing to support or promote physical activity as the primary or secondary treatment goal versus interventions without a motivational interviewing component. The interventions with motivational interviewing had to specify that a component of the intervention included the core principles of motivational interviewing as outlined by Miller and Rollick.^11^ These principles include having a clear focus on the behaviour change (in this case, physical activity), empathetic listening to establish a relationship, and evoking patients’ own motivation for change.^9^ We determined that the study was eligible in terms of intervention content if authors stated motivational interviewing or motivational interviewing techniques were applied. Using a checklist, reviewers allocated eligible comparator interventions to one of three groups: no intervention, minimal control (including usual care) intervention, or active control intervention, all of which used alternative approaches or interventions to promote physical activity that did not include motivational interviewing techniques. To be eligible for inclusion the study had to include a quantitative physical activity outcome at baseline and follow-up.

### Outcomes

Eligible studies needed to report at least one of the physical activity outcomes of interest—total physical activity, moderate to vigorous physical activity (MVPA), or sedentary time, or a combination of these—using a quantitative unit (eg, steps/day, min/day, min/week, energy expenditure, metabolic equivalents (METs)). When a study did not report all the outcomes of interest, we included the study results in the analysis for only the outcomes reported. If studies used more than one method to assess total physical activity and sedentary time outcomes, we prioritised device-measured outcomes over self-reported outcome measures. If more than one device-measured method was reported, we prioritised measures reporting time spent on physical activity, followed by step counts, distance walked, and energy expenditure or metabolic equivalent of task. If no result for a device-measured outcome was available, we extracted self-reported measures such as questionnaires and diaries.

To determine the effect of interventions with motivational interviewing on physical activity over time, we examined effectiveness at 0-3 months, 4-6 months, 7-12 months, and >1 year from baseline.

### Search methods for identification of studies

We systematically searched seven electronic databases (CINAHL, Embase, AMED, Medline, PsychINFO, SPORTDiscus, and Cochrane Central Register of Controlled Trials) for articles, including theses, published from inception until 1 March 2023. Searches were restricted to studies in English language. To locate further relevant publications we performed forward and backwards citation searches of previous systematic reviews. To identify ongoing clinical trials, we searched ClinicalTrials.gov and contacted the authors of published study protocols if there was uncertainty about a trial’s status (ie, if the anticipated completion date was overdue but we could not identify the published study). Supplementary table 1 presents a sample of our search strategy.

### Study selection, data extraction, and risk of bias

After duplicates had been removed, a combination of two reviewers (DS, MM, AH, SZ, PD, GW, MK, NMA) independently screened the titles and abstracts of identified studies using the Cochrane systematic review software Covidence (www.covidence.org).^19^ Two reviewers then independently assessed the full text of articles against the defined eligibility criteria, with discrepancies resolved by discussion or by consultation with a third reviewer. A prespecified and piloted data extraction form was used to obtain key information from included studies on study setting, population characteristics, intervention characteristics (according to the Template for Intervention Description and Replication^20^), and outcome data. Data were extracted by one reviewer and verified by a second reviewer. Disagreements were resolved by consultation with a third reviewer.

### Risk of bias assessment

Two reviewers independently assessed risk of bias of included studies using version 2 of the Cochrane risk of bias tool for randomised trials.^21^ Studies were judged to be at low or high risk of bias or to have some concerns in several domains: randomisation process, deviation from the intended intervention, missing outcome data, measurement of the outcome, and selection of reported results. Overall ratings were taken from the most biased rating across all domains (ie, if one domain was judged to be high then the overall rating was high). Disagreements between reviewers were discussed until consensus was reached.

### Data synthesis and analysis

We extracted the mean and standard deviation (SD) for outcome measures. If these were not reported or unavailable, they were estimated using reported data or graphical figures, or if only medians were available we used these as a direct replacement for mean values, as recommended by the Cochrane Handbook.^22^ We contacted authors for missing data and clarification when necessary. To overcome variability in the way physical activity outcomes were measured in different studies, we calculated the difference in the change in physical activity from baseline (pre-intervention) to follow-up between intervention and comparator groups using standardised mean difference (SMD) with 95% confidence interval (CI). Because studies dealt with missing follow-up data in different ways, to reduce spurious heterogeneity we extracted the complete case data and then used the baseline observation carried forward for missing data to recalculate the change in physical activity.^23^ For studies that were eligible for inclusion but did not provide enough data for meta-analysis, we synthesised the study results narratively.

Pooled data were summarised using Hartung-Knapp-Sidik-Jonkman random effects meta-analysis.^24^ Based on feedback from our patient and public involvement group, to make findings more meaningful for the main findings we transformed the SMDs from the pooled analyses on total physical activity, MVPA, and sedentary time into equivalent weighted mean differences in daily steps (for total physical activity), weekly minutes (for MVPA), and daily minutes (for sedentary time).^22^ For this conversion, we used median SDs of 2940 steps/day for total physical activity, 211 min/week for MVPA, and 87 min/day for sedentary time.^25^ If a study contributed more than one intervention arm to a meta‐analysis, we divided the control group equally between interventions to avoid double counting in the pooled result.

Two types of meta-analyses were performed. For the meta-analyses on overall main effects, we included the longest follow-up measure of physical activity from each study. For meta-analyses split by follow-up assessment time, each study was eligible for inclusion once in each follow-up group (0-3 months, 4-6 months, 7-12 months, and >1 year). If a study reported outcomes at several follow-up time points within each follow-up group (eg, four weeks and 12 weeks), we used the longest follow-up in the analysis (ie, the study was only included once in each follow-up group analysis).

We used the Cochrane Q test to identify heterogeneity, and quantified it using the I^2^ statistic and the between study variance τ^2^. We followed Cochrane Handbook recommendations to interpret I^2^ values (<0.4 representing a small effect, 0.4-0.7 a moderate effect, >0.7 a large effect).^22^ Heterogeneity was explored by determining the effect of several variables on the outcomes: comparator type, intervention durations, outcome assessment method, and participant disease or health condition status.

Funnel plots were generated and Egger’s test was performed to detect small study and publication bias. For all statistical analyses, we considered an α of <0.05 to be statistically significant. STATA SE 17.0 was used for all analyses. The statistical code used in the analysis is available at https://github.com/nerysastbury/MI_SR.git.

To determine the effect of excluding studies at high risk of bias on overall outcomes, we performed a sensitivity analysis. We had planned a sensitivity analysis excluding studies that reported poor intervention fidelity—however, although studies did report assessing fidelity of the intervention, the outcomes of the assessments were poorly reported, resulting in the inability to create discrete groups based on fidelity outcomes, which is required to undertake a sensitivity analysis.

Two reviewers (NMA and SZ) independently rated the certainty of evidence for each outcome using GRADE (Grading of Recommendations Assessment, Development and Evaluation). The certainty of the evidence was assessed for the domains of risk of bias, inconsistency, indirectness, imprecision, and publication bias.^26^

### Patient and public involvement

We convened a focus group of five individuals who self-identified as not being physically active but wanted to increase daily physical activity or had been advised to do so by a healthcare professional. The purpose of the review was described in detail to them, and the findings were explained. Our patient and public involvement panel agreed the review was useful, and it provided feedback on interpretation of the findings and suggested we describe the results in a more meaningful way. This led to our decision to convert SMD into more meaningful outcomes to be more easily interpretable by members of the public, and we present these conversions alongside the main results.

## Results

**Fig 1:**
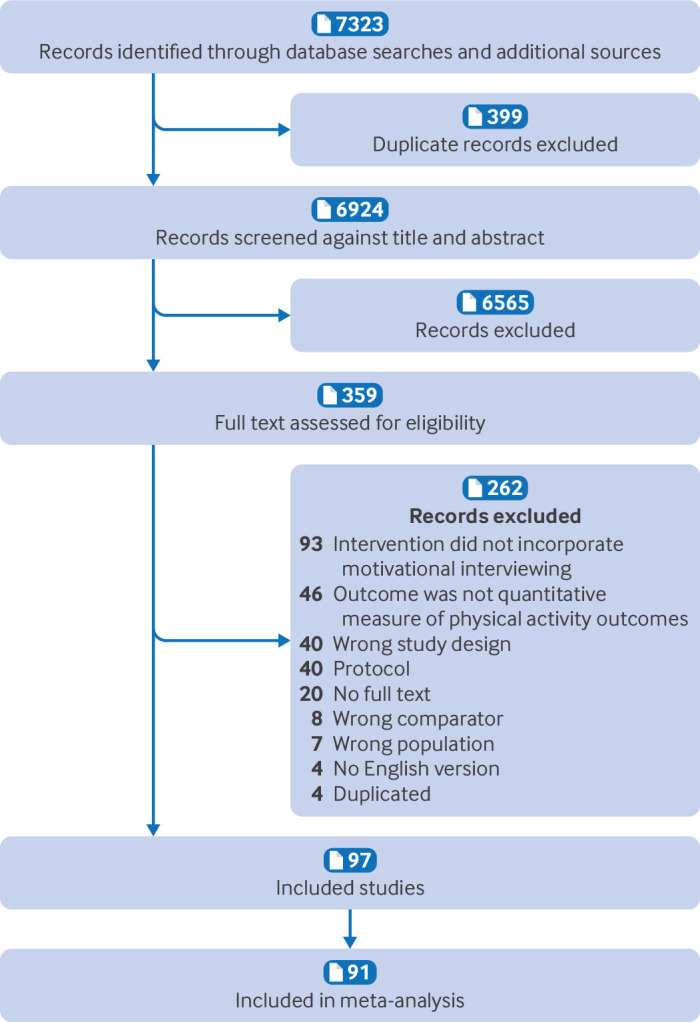
Flow of study selection through review

A literature search on 1 March 2023 identified a total of 7323 unique records, of which 359 full texts were assessed for eligibility. The main reasons for exclusion at the full text stage were that studies did not specify use of motivational interviewing in their intervention or they did not report a quantitative physical activity outcome measure (fig 1). In total, 97 unique randomised controlled trials comparing the effect of 105 interventions comprising motivational interviewing in 27 811 participants were included.^27^ ^28^ ^29^ ^30^ ^31^ ^32^ ^33^ ^34^ ^35^ ^36^ ^37^ ^38^ ^39^ ^40^ ^41^ ^42^ ^43^ ^44^ ^45^ ^46^ ^47^ ^48^ ^49^ ^50^ ^51^ ^52^ ^53^ ^54^ ^55^ ^56^ ^57^ ^58^ ^59^ ^60^ ^61^ ^62^ ^63^ ^64^ ^65^ ^66^ ^67^ ^68^ ^69^ ^70^ ^71^ ^72^ ^73^ ^74^ ^75^ ^76^ ^77^ ^78^ ^79^ ^80^ ^81^ ^82^ ^83^ ^84^ ^85^ ^86^ ^87^ ^88^ ^89^ ^90^ ^91^ ^92^ ^93^ ^94^ ^95^ ^96^ ^97^ ^98^ ^99^ ^100^ ^101^ ^102^ ^103^ ^104^ ^105^ ^106^ ^107^ ^108^ ^109^ ^110^ ^111^ ^112^ ^113^ ^114^ ^115^ ^116^ ^117^ ^118^ ^119^ ^120^ ^121^ ^122^ ^123^

### Characteristics of included studies

Supplementary table 2 provides the characteristics of the included studies. Study sample sizes varied from 23 to 4283 participants, with six studies comprising >1000 participants.^52^ ^57^ ^62^ ^73^ ^82^ ^118^ The median age of participants was 55.5 (interquartile range (IQR) 45.7-64.2) years and median baseline body mass index (BMI) was 28.9 (27.2-30.8). The median proportion of female participants was 66% (34%). Most of the included studies were conducted in high income countries in North America (n=44, 45%), Europe (n=35, 36%), Asia (n=2, 2%), Middle East (n=1, 1%), and Australasia (n=12, 12%), with only three studies (2%) conducted in low and middle income countries (Iran, North Korea, Turkey). Around one quarter of the studies (n=25, 28%) were conducted in generally healthy participants, with the remainder (n=72, 74%) in patients with a health condition or pre-existing disease. The most common health condition or disease of interest was cardiovascular disease (including risk reduction and secondary prevention; n=19, 20%), with fewer studies conducted specifically in people with overweight or obesity (n=10, 10%) and those with musculoskeletal conditions (including osteoarthritis and rheumatoid arthritis; n=9, 9%). Thirty three studies assessed outcomes using device-measured methods such as pedometers or accelerometers, and 47 studies used self-reported methods, including questionnaires, physical activity logs, and diaries.

Motivational interviewing was delivered in a variety of ways between the studies (see supplementary table 3). The number of motivational interviewing sessions offered to participants assigned to receive interventions with motivational interviewing ranged from one to 70 over an intervention period of one to 24 months, with a median duration of 32.9 (23.1-60.0) minutes each. In half of the motivational interviewing interventions (n=53, 50%), treatment duration lasted up to three months, with the remaining interventions reporting longer durations: 4-6 months (n=25, 24%), 7-12 months (n=24, 23%), and >1 year, to a maximum of 24 months (n=3, studies, 3%). Most studies (n=89, 92%) reported who delivered the intervention with motivational interviewing, and 74 studies (76%) reported the training and qualifications of the interventionists, which ranged from undergraduate students to experienced psychologists, with considerable variation in amount of training and experience of delivering motivational interviews.

Most interventions with motivational interviewing (n=96, 91%) were delivered in a one-to-one format. Five interventions (5%) were delivered in a combination of individual and group motivational interviewing sessions, and four interventions (4%) offered group sessions only. Motivational interviewing was delivered using a range of modalities, with 33 interventions (31%) delivered face-to-face, 31 (30%) by telephone, and six (6%) through the internet or a mobile application, and 35 studies (n=33) used a combination of in-person and remote delivery methods.

Most of the interventions with motivational interviewing were compared with no intervention or minimal control interventions (n=74, 70%) and 31 (30%) had an active comparator that was either an alternative behavioural intervention to promote physical activity that did not include motivational interviewing of similar (n=11, 10%) or less intensity (n=20, 19%) to the intervention with motivational interviewing being delivered.

### Risk of bias and quality assessment

**Fig 2:**
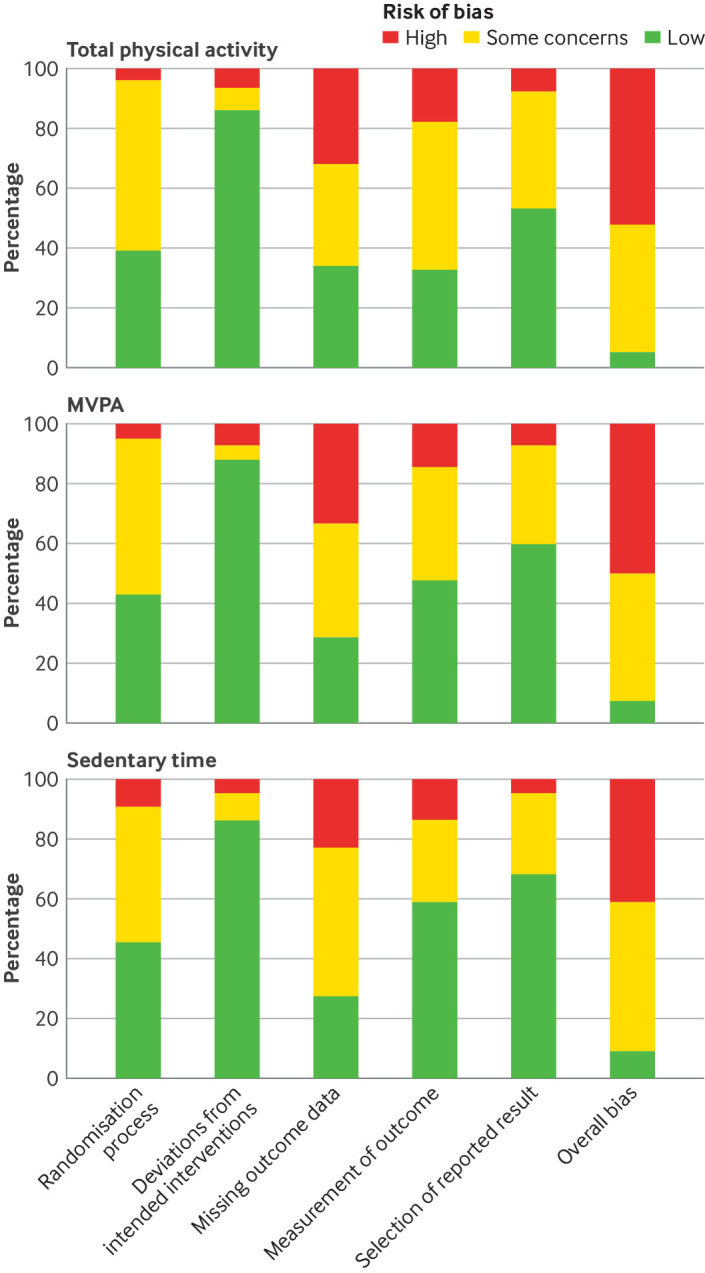
Risk of bias in studies reporting outcomes for total physical activity, MVPA, and sedentary time. CI=confidence interval; MVPA=moderate to vigorous physical activity

In studies reporting total physical activity, most (n=41, 52%) were judged to be at overall high risk of bias, four (5%) were judged to be at overall low risk of bias, and 34 (43%) had some concerns (fig 2).

The main reason for a high overall risk of bias judgement was primarily from bias due to the missing outcome data domain (eg, emphasis on per protocol analysis, incorrect procedures to account for missing data, loss to follow-up concerns). Supplementary figure 1 presents details of the risk of bias assessments for individual studies.

Funnel plot asymmetry was found among studies included in the meta-analysis of motivational interviewing on total physical activity, MVPA, and sedentary time (Egger’s g=2.51 (95% CI 1.34 to 3.66), 2.34 (0.71 to 3.97), and −2.62 (−4.49 to −0.74), respectively) suggesting that studies could be missing in the literature that reported null or negative findings of motivational interviewing or small study effects (supplementary figure 2).^124^

### Overall effect of motivational interviewing on physical activity

**Fig 3:**
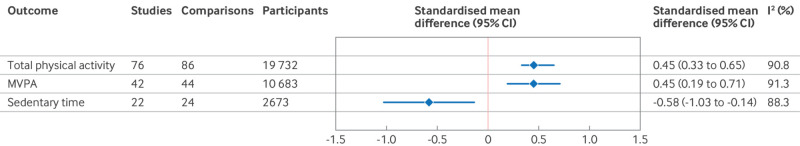
Summary standardised mean differences for overall meta-analysis. CI=confidence interval; MVPA=moderate to vigorous physical activity

Interventions including motivational interviewing were superior to comparators for total physical activity (n=76, 19 732 participants, 86 comparisons; SMD 0.45, 95% CI 0.33 to 0.65; I^2^=90.8%), equivalent to 1323 extra steps/day (95% CI 970 to 1911) (fig 3, supplementary figure 3). Three studies could not be included in this meta-analysis because they failed to report SDs, or information was insufficient that would permit calculation of SDs. Of these studies excluded from the meta-analysis, none reported a difference in physical activity between motivational interviewing and control groups.^41^ ^48^ ^90^

Interventions including motivational interviewing were also superior to comparators for MVPA (n=42, 10 683 participants, 44 comparisons; SMD 0.45 (95% CI 0.19 to 0.71); I^2^=91.3%), equivalent to 95 extra min/week (95% CI 40 to 150) (fig 3, supplementary figure 4). Three studies reporting MVPA outcomes could not be included in the meta-analysis because they failed to report SDs or information to permit calculation of SDs was insufficient. However, these studies reported minimal or no effect of interventions with motivational interviewing on MVPA outcomes.^41^ ^49^ ^52^

Interventions including motivational interviewing were superior to comparators in reducing sedentary time (n=23, 2673 participants, 24 comparisons; SMD −0.582, 95% CI 1.03 to 0.14; I^2^=88.3%) (fig 3, supplementary figure 5) equating to 51 fewer minutes (95% CI −90 to −12) of sedentary time each day.

### Effect of motivational interviewing on physical activity and sedentary time

#### Comparator type

As heterogeneity in the outcome measures was considerable, we analysed whether the type of comparator groups had an influence on the overall findings.

*No or minimal comparator intervention*—Interventions including motivational interviewing were superior to no or minimal comparator interventions (eg, usual care) for total physical activity (n=55, 16 079 participants, 60 comparisons; SMD 0.56 (95% CI 0.37 to 0.76); I^2^=90.4%), MVPA (n=28, 8318 participants, 30 comparisons; 0.45 (0.24 to 0.65); I^2^=90%), and sedentary time (n=13, 1734 participants, 15 comparisons; −0.59 (1.18 to −0.01); I^2^=85.5%) (supplementary figures 6-8).

*Less intensive comparator interventions*—Some studies compared interventions including motivational interviewing with other active interventions of lower intensity (eg, educational website on how to increase physical activity, single lecture on self-management) that did not include motivational interviewing. Evidence was lacking for a difference between groups for total physical activity (n=18, 2312 participants, 18 comparisons; 0.23 (−0.09 to 0.56); I^2^=92.9%), MVPA (n=12, 1997 participants, 12 comparisons; 0.61 (−0.436 to 1.65); I^2^=94.3%), or sedentary time (n=7, 598 participants, 7 comparisons; −0.65 (−1.80 to 0.51); I^2^=93.7%) (supplementary figure 9).

*Similar intensity comparator interventions*—To isolate the effect of motivational interviewing on physical activity in complex interventions, we compared interventions including motivational interviewing with comparator interventions of similar intensity that did not include motivational interviewing. Evidence was lacking for a difference between groups for total physical activity (n=7, 1340 participants, eight comparisons; 0.43 (−0.08 to 0.927); I^2^=83.3%), MVPA n=2, 368 participants, two comparisons; 0.02 (−0.55 to 0.59); I^2^=0%), or sedentary time (n=1, 302 participants, one comparison; −0.08 (−0.31 to 0.152) (supplementary figure 10).

#### Outcome assessment method

We compared effects in studies with device-measured outcomes with studies using self-reported outcome assessment methods. No significant between group heterogeneity was found for device-measured and self-reported measured groups for total physical activity (P=0.33), MVPA (P=0.43), or sedentary time (P=0.07), and heterogeneity remained substantial within these subgroups (supplementary figures 11-13).

*Device-measured outcome assessment methods*—In the studies using device-measured outcome assessment methods, evidence suggested that interventions with motivational interviewing were superior to comparators for total physical activity (n=25, 19 732 participants, 27 comparisons; 0.37 (0.10 to 0.64); I^2^=91.3%), MVPA (n=42, 10 683 participants, 44 comparisons; 0.61 (0.19 to 0.71); I^2^=90.7%), and sedentary time (n=11, 1123 participants, 13 comparisons; −0.26 (−0.89 to 0.38); I^2^=86.8%).

*Self-reported outcome assessment methods*—For the studies that used self-reported outcome assessment methods, interventions with motivational interviewing were superior to comparators for total physical activity (n=54, 15 152 participants, 59 comparisons; 0.53 (0.34 to 0.73); I^2^=90.6%), MVPA (n=23, 8243 participants, 24 comparisons; 0.37 (0.11 to 0.62); I^2^=91.7%), and sedentary time (n=9, 1550 participants, 11 comparisons; −1.01 (−1.65 to −0.37); I^2^=90.1%).

### Durability of effectiveness of motivational interviewing over time

**Fig 4:**
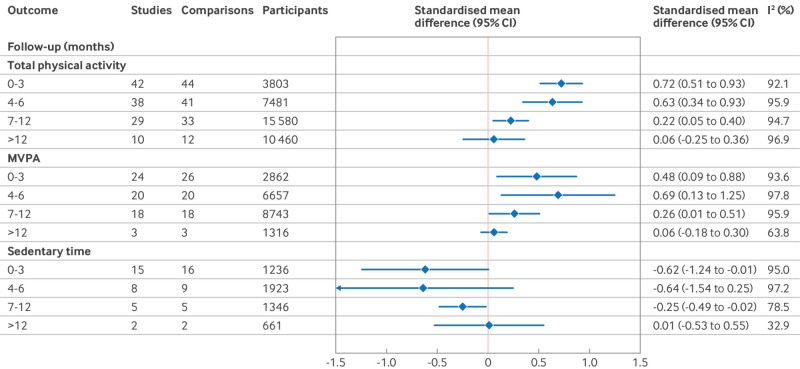
Summary standardised mean differences for meta-analysis at each follow-up time. CI=confidence interval; MVPA=moderate to vigorous physical activity

We examined the effectiveness of interventions with motivational interviewing at 0-3 months, 4-6 months, 7-12 months, and >1 year from baseline. For all follow-up times up to 12 months, interventions with motivational interviewing were superior to comparators for total physical activity, MVPA, and sedentary time, with a trend for declining effect size with increasing duration of follow-up (fig 4). For studies with follow-up beyond one year, evidence that interventions with motivational interviewing were any different from comparators for any of the three outcomes was lacking (fig 4, supplementary figures 14-25). Heterogeneity between studies in each outcome at each time point was substantial, apart for sedentary time at >1 year follow-up, where only two eligible studies were included in the meta-analysis (I^2^=0%).

### Effect of treatment duration

For interventions of up to three months’ duration, those with motivational interviewing were superior to comparators for total physical activity at the end of the intervention period (n=34, 2182 participants, 32 comparisons; 0.70 (0.46 to 0.92); I^2^=79.2%) (supplementary figure 26), but evidence was lacking for a statistically significant difference between groups after the intervention ended (supplementary figures 27 and 28). Heterogeneity was substantial at all time points.

For interventions of 4-6 months’ duration, those with motivational interviewing were superior to comparators for total physical activity at the end of the intervention period (n=19 studies, 3218 participants, 20 comparisons; 0.99 (0.49 to 1.49); I^2^=94.1%) (supplementary figure 29), and these effects were sustained at 7-12 months follow-up (n=4, 1221 participants, 2 comparisons; 0.23 (0.10 to 0.37); I^2^=0%) (supplementary figure 30), but not for follow-up beyond one year (n=2, 648 participants, 2 comparisons; 0.004 (−0.18 to 0.19); I^2^=0%) (supplementary figure 31). Heterogeneity was substantial at the end of intervention follow-up, but the results of longer term follow-up showed no heterogeneity.

For interventions of 7-12 months’ duration, those with motivational interviewing were superior to comparators for total physical activity at the end of the intervention period (n=17, 11 262 participants, 21 comparisons; 0.26 (0.06 to 0.47); I^2^=89.7%) (supplementary figure 32), but evidence of a difference between groups in studies reporting follow-up beyond one year was lacking (n=5 studies, 8358 participants, 7 comparisons; 0.13 (−0.11 to 0.37); I^2^=78.6%) (supplementary figure 33).

For interventions of 12 months’ duration or longer, no statistically significant difference was found between interventions with motivational interviewing and comparators for physical activity at the end of the intervention period (n=3 studies, 1103 participants, 73 comparisons; −0.20 (–2.24 to 1.85); I^2^=98.2%) (supplementary figure 34).

For MVPA outcomes, interventions with motivational interviewing of up to 12 months’ duration were superior to comparators at 0-3 months follow-up (supplementary figure 35), but no significant differences were evident at longer follow-up times (supplementary figures 36-38). Interventions with motivational interviewing of longer than one year’s duration were not statistically significantly different from comparators at any follow-up time (supplementary figures 35-38).

For sedentary time outcomes, evidence for interventions with motivational interviewing being superior to comparators was lacking, regardless of intervention duration at any follow-up time (supplementary figures 39-42).

### Impact of participant health status

In studies of people with a pre-existing health condition or disease, evidence suggested that interventions with motivational interviewing led to greater increases in total physical activity (n=60, 10 525 participants, 66 comparisons; 0.55 (0.35 to 0.76); I^2^=91.2%) and MVPA (n=34, 7094 participants, 36 comparisons; 0.46 (0.26 to 0.67); I^2^=90.3%), and reductions in sedentary time (n=18, 2171 participants, 20 comparisons; −0.72 (−1.2 to −0.22); I^2^=87.8%) (supplementary figures 43-45).

In studies of people not specifically selected on the basis of a pre-existing health condition or disease, interventions with motivational interviewing were superior to comparators for total physical activity (n=19 studies, 9207 participants, 20 comparisons; 0.35 (0.13 to 0.57); I^2^=89.6%). Evidence that interventions with motivational interviewing were superior to comparators for MVPA (n=8, 3589 participants; 0.43 (−0.99 to 1.84); I^2^=94.6%) or sedentary time (n=4, 502 participants; −0.02 (−1.27 to 1.23); I^2^=90.5%) (supplementary figures 46-48) was lacking.

### Sensitivity analysis

We conducted sensitivity analyses on the principal outcomes, excluding those studies judged at overall high risk of bias.

When studies at high risk of bias were excluded from analysis, interventions with motivational interviewing remained superior to comparators for total physical activity (n=38, 8467 participants, 40 comparisons; 0.54 (0.31 to 0.78); I^2^=89.2%) (supplementary figure 49) but showed no difference for MVPA (n=21, 4935 participants, 22 comparisons; 0.41 (−0.07 to 0.90); I^2^=92.1%) (supplementary figure 50) or sedentary time (n=13, 1476 participants, 3 comparisons; −0.22 (−0.70 to 0.26); I^2^=82.7%) (supplementary figure 51).

### Certainty of evidence

#### Total physical activity

Certainty in the effect estimates for interventions with motivational interviewing on total physical activity was rated as low.

Sensitivity analysis removing studies judged at overall high risk of bias did not affect the outcome on total physical activity. Heterogeneity was, however, substantial (I^2^=90.8%), which could not be fully explained by comparator type, follow-up duration, intervention duration, disease status of participants, device outcome assessment method, or risk of bias. Furthermore, funnel plot asymmetry was evident, suggesting potentially over-optimistic estimates of the effect of interventions with motivational interviewing on physical activity, possibly due to publication and small study bias. Certainty in the effect estimate was therefore downgraded one level owing to unexplained inconsistency, and one level owing to publication bias.

#### MVPA

Certainty in the effect estimates for interventions with motivational interviewing on MVPA was rated as very low.

Heterogeneity was substantial (I^2^=91.3%), which could not be fully explained by comparator type, follow-up duration, intervention duration, disease status of participants, device outcome assessment method, or risk of bias. Funnel plot asymmetry was evident, suggesting publication or small study bias, and sensitivity analysis excluding studies judged to be at high risk of bias resulted in no difference between interventions with motivational interviewing and comparators. Certainty of evidence was therefore downgraded two levels owing to very serious unexplained inconsistency, and one level owing to publication bias.

#### Sedentary time

Certainty of the effect estimates for sedentary time was very low.

Heterogeneity was substantial (I^2^=88.3%), which could not be fully explained. Funnel plot asymmetry was significant, and the sensitivity analysis removing studies judged at overall high risk of bias resulted in no difference between interventions with motivational interviewing and comparators, suggesting risk of bias was high. Certainty of evidence for sedentary time was therefore downgraded two levels owing to very serious unexplained inconsistency, and one level owing to publication bias.

## Discussion

Overall, 97 randomised controlled trials examining the effectiveness of 105 interventions comprising motivational interviewing to increase physical activity were included in this review. The totality of the evidence showed that interventions with motivational interviewing led to a greater increase in total physical activity (an extra 1300 steps/day) and MVPA (an extra 95 min/day) and reductions in sedentary time (50 fewer min/day) compared with comparator interventions. Certainty of the evidence for interventions with motivational interviewing promoting physical activity was low for total physical activity and very low for MVPA and reduction in sedentary time, with few high quality studies.

Effect sizes for studies using device-measured total physical activity and sendentary time outcomes were more modest, and for MVPA were higher than for studies using self-reported outcomes. This finding is consistent with reports that self-reported measures can over-report physical activity levels.^125^

We found no evidence of an effect when interventions with motivational interviewing were compared with comparator interventions of similar intensity. Most studies were judged to be at high risk of bias, and when these were removed, only the effect on total physical activity remained.

Subgroup analyses helped to contextualise the circumstances when interventions with motivational interviewing might be superior to comparators. The effectiveness of interventions with motivational interviewing diminished with duration of follow-up, with no evidence of a benefit for interventions lasting more than one year. We also found no evidence of differences in any outcomes beyond the end of the active intervention period.

In groups with pre-existing health conditions, interventions with motivational interviewing increased total physical activity and MVPA and reduced sedentary time. However, in general population groups, not selected on the basis of a pre-existing health condition or disease, although interventions with motivational interviewing were effective on total physical activity, we found no evidence to suggest they were effective at increasing MVPA or reducing sedentary time.

### Strengths and limitations of this review

To limit bias and minimise confounding, we included only randomised controlled trials, did not exclude studies based on year of publication, and followed established Cochrane methods.^126^ Our searches were designed to be comprehensive and therefore included many studies, with interventions of variable type, content, and duration. As a result, whereas previous reviews have had a more targeted approach, we included many more studies in this review. We included studies regardless of the method used to assess physical activity outcomes as long as it quantified physical activity. Furthermore, guided by our patient and public involvement group, who suggested we should make the findings easier to understand, we converted the SMD to more meaningful physical activity outcomes using established methods.^22^

Several limitations should also be considered when interpreting the results. Although we systematically reviewed the evidence from randomised controlled trials, it is difficult to isolate the effectiveness of motivational interviewing because it is was almost always included alongside other behavioural components. Value may be had in exploring evidence from other types of study design, which could provide insight into possible explanations or mechanisms for the observed effects.The variability in interventions and comparators likely contributes to the substantial heterogeneity we observed in most analyses. It is also likely, given the breadth of the inclusion criteria for this study, that differences in study populations, interventions, and outcome assessment methods, and other factors such as geographical or temporal differences, contributed to heterogeneity, and it was unlikely our analysis would fully explain these differences.

We found significant evidence of bias in the findings. Our searches were limited to studies published in English, but we only identified four other eligible papers, and given the large number of studies included in this review we believe the exclusion of these papers is not likely to have meaningfully affected the overall findings. Of note, the included studies were undertaken predominately in female participants with overweight or obesity from high income countries, which could limit the generalisability of results reported here to other populations.

### Comparison with other studies

Several previous systematic reviews have explored the effects of motivational interviewing on physical activity.^15^ ^127^ ^128^ ^129^ The most notable previous systematic review included only eight trials and the meta-analysis reported that there was a very small effect in increasing physical activity levels favouring the interventions with motivational interviewing in people with chronic conditions (SMD 0.19, 95% CI 0.06 to 0.32).^15^

One study reported a systematic review and meta-analysis of the effectiveness of interventions with motivational interviewing for increasing physical activity in older adults. Only three trials with 84 participants were included in that review, and no difference was found between interventions with motivational interviewing and comparators for increasing physical activity (SMD −0.02, 95% CI 0.05 to 0.46, I^2^=16%).^127^ Another review included 72 randomised controlled trials exploring the effect of motivational interviewing on a range of health outcomes. To our knowledge, this is the only previous review to explore effects of motivational interviewing at different horizon or follow-up time frames. The authors reported a large effect size in favour of interventions including motivational interviewing for improving a composite measure of diet and physical activity (Cohen’s *d*=0.78, 95% CI 0.41 to 1.16), but the findings from studies targeting diet or physical activity behaviour were based on only four studies and the authors did not explore the effect on physical activity or exercise in itself, nor did they break down the effects of motivational interviewing on diet and exercise outcomes by follow-up time.^129^

Previous research suggests that an increase of 1000 steps/day equates to about 10 mins of moderate intensity activity.^130^ Here we show that, overall, interventions with motivational interviewing increase physical activity by about 1300 steps/day, or an extra 95 min/day of moderate to vigorous intensity physical activity. The mean population average MVPA in England is 118 min/week in men and 80 min/week in women. The low levels of participation in physical activity coupled with longer periods of sedentary time (mean 8.65 h/day),^131^ in the population are concerning and may be contributing to adverse health outcomes.^132^ ^133^

To realise these health benefits the intervention effect needs to be sustained. We hypothesised that interventions including motivational interviewing may prove more durable than some other interventions. However, as with many other interventions to increase physical activity, effectiveness waned over time. Moreover, we noted that the effects were only observed when interventions with motivational interviewing were compared with a no or minimal intervention comparator. No significant effects were observed when complex interventions with motivational interviewing were compared with interventions of similar intensity but not including motivational interviewing. This suggests the observed effect of motivational interviewing may be attributable to the extent of support provided, rather than a specific effect of motivational interviewing itself.

Interventions with motivational interviewing were effective in people with pre-existing health conditions for all outcomes but were ony effective in the general population for total physical activity. Motivational interviewing requires intensive formal training and lifelong continued professional development to instruct interventionist to deliver the intervention to a satisfactory standard. These findings should therefore sound a note of caution in adopting motivational interviewing as part of routine interventions to increase physical activity given the added costs. Because the findings indicate that interventions with motivational interviewing are effective in promoting physical activity in specific clinical populations, however, it may be cost effective for people who could benefit from short term improvements in physical activity, such as before surgery or during pregnancy.

Other systematic reviews have found similar increases in physical activity to those reported here for less expensive, self-directed interventions, such as wearable devices.^134^ ^135^

### Conclusions

Despite considerable interest in motivational interviewing as a behaviour change technique and a large number of studies, the certainty of evidence for the effectiveness of interventions with motivational interviewing to increase total physical activity is low and very low for MVPA and sedentary time. Overall, the evidence showed that interventions with motivational interviewing led to increases in physical activity and MVPA and reductions in sedentary time, but the effect diminished over time and did not persist beyond the active intervention period. Effects were largely driven by studies that compared interventions with motivational interviewing versus those with minimal or no intervention comparator. In the small number of studies that compared interventions differing only in the presence of motivational interviewing, evidence of a difference in any physical activity outcome was lacking.

**What is already known on this topic**

Motivational interviewing is a person centred, behaviour change approach, and it is recommended for interventions to promote health related behaviour change
Previous meta-analyses of small numbers of trials examining the effect of motivational interviewing on physical activity reported that motivational interviewing was superior to comparators in people with chronic health conditions
These reviews could have overestimated effects by comparing motivational interviewing interventions with no or minimal controls and by focusing on effects at the end of the intervention rather than longer term follow-up

**What this study adds**

Across all studies in this meta-analysis, interventions with motivational interviewing were associated with significant increases in total physical activity (1323 steps/day) and moderate to vigorous physical activity (MVPA, 95/min/week) and reduction in sedentary behaviour (−51 min/day).
Interventions with motivational interviewing were only better than comparators in the short term and no durable effect of motivational interviewing was found beyond the intervention period
There was also no evidence that behavioural interventions with motivational interviewing were any better at increasing physical activity compared with other behavioural interventions of similar intensity that did not include motivational interviewing

## Data Availability

The statistical code used in the analysis is available from https://github.com/nerysastbury/MI_SR.git .

## Web extra

Extra material supplied by authors

## Notes

Contributors: SZ and DS contributed equally and are joint first authors. NMA, LM, RS, and SAJ conceived the study. DS and NMA developed the search strategy. DS, SZ, MM, GW, AH, PD, MK, and NMA screened and selected studies. DS and SZ performed analyses, with input from NMA. DS and SZ wrote the first draft of the manuscript with critical input from all authors. All authors have read and approved the final submitted version. NMA acts as guarantor. The corresponding author attests that all listed authors meet authorship criteria and that no others meeting the criteria have been omitted.

Transparency: The lead author (NMA) affirms that the manuscript is an honest, accurate, and transparent account of the study being reported; that no important aspects of the study have been omitted; and that any discrepancies from the study as originally planned (and, if relevant, registered) have been explained.

Dissemination to participants and related patient and public communities: We plan to share the findings of this study with academics working in physical activity and behaviour change to enhance the quality of the evidence base. We will also discuss our findings with clinicians and commissioners by presenting at relevant events, publishing companion blogs and/or non-specialist articles, and using social media opportunities.

Provenance and peer review: Not commissioned; externally peer reviewed.
