# Supplementary material for: Effectiveness of behavioural interventions with motivational interviewing on physical activity outcomes in adults: systematic review and meta-analysis
Source: BMJ. 2024 Jul 10;386:e078713. doi: 10.1136/bmj-2023-078713 (PMC11234249; doi:10.1136/bmj-2023-078713)

Supplementary Materials

Supplementary Table 1: Example search strategy. 1

Supplementary Table 2: Characteristics of included studies 2

Supplementary Table 3: Intervention characteristics 8

Supplementary Figure 1: Risk of bias assessment for individual studies 40

Supplementary Figure 2: Funnel plots with pseudo 95% confidence intervals of studies reporting total PA (A) MVPA (B) and sedentary time (C) outcomes. 41

Supplementary Figure 3: Forest plot on studies reporting total physical activity. 42

Supplementary Figure 4: Forest plot on studies reporting moderate & vigorous physical activity (longest follow-up). 43

Supplementary Figure 5: Forest plot on studies reporting sedentary time (longest follow-up). 44

Supplementary Figure 6: Forest plot of studies comparing MI interventions with no or minimal intervention comparators on Total PA. 45

Supplementary Figure 7: Forest plot of studies comparing MI interventions with no or minimal intervention comparators on MVPA 46

Supplementary Figure 8: Forest plot of studies comparing MI interventions with no or minimal intervention comparators on sedentary time. 47

Supplementary Figure 9: Forest plot of studies comparing MI interventions with other active interventions of lower intensity that did not include MI on Total PA, MVPA and sedentary time. 48

Supplementary Figure 10: Forest plot of studies comparing MI interventions vs. comparator interventions of similar or lesser intensity on all outcomes. 49

Supplementary Figure 11: Forest plot of studies using device measured outcomes on total PA, MVPA and sedentary time outcomes 50

Supplementary Figure 12: Forest plot of studies using self-reported outcome assessment methods for total PA outcomes. 51

Supplementary Figure 13: Forest plot of studies using self-reported outcome assessment methods for MVPA and sedentary time outcomes 52

Supplementary Figure 14: Forest plot of studies reporting total PA at 0-3 months follow-up 53

Supplementary Figure 15: Forest plot of studies reporting total PA at 4-6 months follow-up 54

Supplementary Figure 16: Forest plot of studies reporting total PA at 7-12 months follow-up 55

Supplementary Figure 17: Forest plot of studies reporting total PA of >1 year follow-up 56

Supplementary Figure 18: Forest plot of studies reporting total MVPA at 0-3 months follow-up 57

Supplementary Figure 19: Forest plot of studies reporting total MVPA at 4-6 months follow-up 58

Supplementary Figure 20: Forest plot of studies reporting total MVPA at 7-12 months follow-up 59

Supplementary Figure 21: Forest plot of studies reporting total MVPA at >1 year follow-up 60

Supplementary Figure 22: Forest plot of studies reporting sedentary time at 0-3 months follow-up 61

Supplementary Figure 23: Forest plot of studies reporting sedentary time at 4-6 months follow-up 62

Supplementary Figure 24: Forest plot of studies reporting sedentary time at 7-12 months follow-up 63

Supplementary Figure 25: Forest plot of studies reporting sedentary time >1 year follow-up 64

Supplementary Figure 26: Forest plot of studies with interventions 0-3 months duration reporting total physical activity outcomes at 0-3 months follow-up 65
Supplementary Figure 27: Forest plot of studies with interventions 0-3 months duration reporting total physical activity outcomes at 4-6 months follow-up 66
Supplementary Figure 28: Forest plot of studies with interventions 0-3 months duration reporting total physical activity outcomes at 7-12 months follow-up 67
Supplementary Figure 29: Forest plot of studies with interventions 4-6 months duration reporting total physical activity outcomes at 0-3 months follow-up 68
Supplementary Figure 30: Forest plot of studies with interventions 4-6 months duration reporting total physical activity outcomes at 7-12 months follow-up 69
Supplementary Figure 31: Forest plot of studies with interventions 4-6 months duration reporting total physical activity outcomes at >1 year follow-up......70
Supplementary Figure 32: Forest plot of studies with interventions 7-12 months duration reporting total physical activity outcomes at 7-12 months follow-up 71
Supplementary Figure 33: Forest plot of studies with interventions 7-12 months duration reporting total physical activity outcomes at >1 year follow-up......72
Supplementary Figure 34: Forest plot of studies with interventions >1 year duration reporting total physical activity outcomes at >1 year......73
Supplementary Figure 35: Forest plot of studies with interventions reporting MVPA outcomes at 0-3 months follow-up by intervention duration 74
Supplementary Figure 36: Forest plot of studies with interventions reporting MVPA outcomes at 4-6 months follow-up by intervention duration 75
Supplementary Figure 37: Forest plot of studies with interventions reporting MVPA outcomes at 7-12 months follow-up by intervention duration 76
Supplementary Figure 38: Forest plot of studies reporting MVPA outcomes at >1 year follow-up by intervention duration......77
Supplementary Figure 39: Forest plot of studies with interventions reporting sedentary time outcomes at 0-3 months follow-up by intervention duration......78
Supplementary Figure 40: Forest plot of studies with interventions reporting sedentary time outcomes at 4-6 months follow-up by intervention duration......79
Supplementary Figure 41: Forest plot of studies with interventions reporting sedentary time outcomes at 7-12 months follow-up by intervention duration. 80
Supplementary Figure 42: Forest plot of studies with interventions reporting sedentary time outcomes at >1 year follow-up by intervention duration. 81
Supplementary Figure 43: Total PA outcomes in studies conducted in people with pre-existing disease or health condition......82
Supplementary Figure 44: MVPA outcomes in studies conducted in people with pre-existing disease or health condition......83
Supplementary Figure 45: Sedentary time outcomes in studies conducted in people with pre-existing disease or health condition......84
Supplementary Figure 46: Total PA outcomes in studies conducted in healthy people......85
Supplementary Figure 47: MVPA outcomes in studies conducted in healthy people......86

Supplementary Figure 48: Sedentary time outcomes in studies conducted in healthy people 87
Supplementary Figure 49: Sensitivity analysis of studies reporting total PA outcomes excluding studies judged to be at overall high risk of bias 88
Supplementary Figure 50: Sensitivity analysis of studies reporting MVPA outcomes excluding studies judged to be at overall high risk of bias 89
Supplementary Figure 51: Sensitivity analysis of studies reporting sedentary time outcomes excluding studies judged to be at overall high risk of bias 90

Supplementary Table 1: Example search strategy.

Medline (Ovid MEDLINE® Epub Ahead of Print, In-Process & Other Non-Indexed Citations, Ovid MEDLINE® Daily and Ovid MEDLINE®) 1946 to present	
1	Motivational Interviewing/
2	motivation/ and Counseling/
3	(motivat* adj2 (interview* or counsel* or advice* or session?)).ti,ab,kw.
4	(motivat* adj2 (practi* or behavi*)).ti,ab,kw.
5	(motivat* adj2 (enhanc* or improv* or encourage* or facilitat*)).ti,ab,kw.
6	"motivation*".m_titl.
7	1 or 2 or 3 or 4 or 5 or 6
8	Physical Activity/
9	Exercise/
10	Physical fitness/
11	Physical Endurance/
12	Resistance training/
13	8 or 9 or 10 or 11
14	randomized controlled trial.pt.
15	controlled clinical trial.pt.
16	randomized.ab.
17	placebo.ab.
18	randomly.ab.
19	trial.ab.
20	groups.ab.
21	13 or 14 or 15 or 16 or 17 or 18 or 19
22	exp animals/ not humans.sh.
23	21 not 22
24	7 and 13
25	23 and 24

Supplementary Table 2: Characteristics of included studies

**Study ID**	Country	Participants					Measurement type	Specifc outcome measure
		**N**	**Mean age (years)**	**% Female**	**BMI (kg/m²)**	**Health conditions/Diseases**		
Albright 2014	US	311	31.9	100	27.9	Healthy	Device-measured	Lifecorder EX accelerometer (NewLifestyles NL-2200, Inc.,MO)
Alothman 2022	Saudi Arabia	50	33.7	82.1	25.4	Healthy	Self-reported	The Global Physical Activity Questionnaire (GPAQ)
Anderson 2014	UK	329	63.6	26	30.7	Adenoma and overweight	Device-measured	SenseWear armband accelerometer (BodyMedia, Pittsburgh, PA)
Anderson 2018	UK	78	47.1	88	32.7	Family history of colorectal or breast cancer	Device-measured	SenseWear armband accelerometer (BodyMedia, Pittsburgh, Pennsylvania, USA)
Ang 2013	US	216	45.8	95	31.4	Fibromyalgia	Device-measured	ActiGraph GT1M accelerometer (Pensacola, FL USA)
Arbillaga-Etxarri 2018	Spain	407	69	13		COPD	Device-measured	Dynaport accelerometer; (McRoberts BV, The Hague, The Netherlands)
Asvat 2014	US	66	56.2	100	28.6	Breast cancer	Self-reported	Modified version of the Godin Leisure-Time Exercise Questionnaire
Aunger 2020	UK	35	73.1	57	30.7	Osteoarthritis	Device-measured	ActivPal3 accelerometer (PAL Technologies Ltd, Glasgow, UK)
Baig 2013	US	100	53.7	81	31.7	Diabetes	Self-reported	Summary of Diabetes Self-Care Activities (SDSCA) scale
Barrett 2018	Australia	72	53	75	30.8	Healthy	Device-measured	Actigraph wGT3X-BT accelerometer (Pensacola, USA)
Becker 2008	Germany	899	48.8	60		Low back pain	Self-reported	Freiburg Questionnaire on Physical Activity (FQPA)
Befort 2008	US	44	44.3	100	39.8	Obesity	Self-reported	The Community Healthy Activities Model Program for Seniors (CHAMPS) physical activity measure
Bennett 2007	New Zealand	56	57.8	89	28.1	Cancer	Self-reported	The Community Healthy Activities Model Program for Seniors (CHAMPS) Physical Activity Questionnaire for Older Adults

Bennett 2008	New Zealand	86	58	90		Healthy	Self-reported	The Community Healthy Activities Model Program for Seniors (CHAMPS) Physical Activity Questionnaire for Older Adults
Benzo 2016*	US	215	68	55		COPD	Device-measured	BodyMedia armband accelerometer (BodyMedia, Pittsburgh, PA)
Berlant 2004	US	237	50.3	58	28.4	Healthy	Self-reported	Self-reported physical activity logs
Blackford 2016	Australia	401	60.9	66	31.1	Metabolic syndrome	Self-reported	The International Physical Activity Questionnaire Short Form (IPAQ-SF)
Bombardier 2008	US	130	46.3	78		Multiple sclerosis	Self-reported	The Health Promoting Lifestyle Profile II (HPLP-II)
Bombardier 2013	US	92	48.5	86	29	Major depressive disorder with multiple sclerosis	Self-reported	Seven Day PA Recall (7 Day PAR) interview
Brodie 2005	UK	92	77.7			Chronic heart failure	Self-reported	Leisure-time physical activity questionnaire and a 3-day physical activity diary.
Brown 2015	US	760	53	64	31.2	Stroke risk	Self-reported	The modified Stanford 7 day Physical Activity Recall Instrument
Burtin 2015*	Belgium	80	66.5	82.5	25.5	COPD	Device-measured	Minimod (McRoberts BV, The Hague, the Netherlands) and the SenseWear Pro Armband accelerometer (SWA; Body Media, Inc., Pittsburgh, PA, USA)
Celano 2018*	US	128	63.1	59	30	Acute coronary syndrome	Device-measured	Actigraph GT3X+ accelerometers (Actigraph, Pensacola, FL USA)
Celano 2020	US	30	70.8	27		Heart failure	Device-measured	Accelerometer
Collins 2019	US	69	58.7	58		CVD risk	Device-measured	Striiv pedometer (LA, USA)
Conn 2003	US	143	75	100		Multiple chronic conditions	Device-measured	Digiwalker pedometer (Shropshire, UK)
Coumans 2022*	Netherlands	1142	52.1	60.6	26.4	Healthy	Self-reported	Dutch Short Questionnaire to ASsess Health (SQUASH)
De Vries 2016	Netherlands	130	78.5	72		Mobility problems	Self-reported	LASA Physical Activity Questionnaire (LAPAQ)
Dennett 2018	Australia	46	59	63	33	Cancer	Device-measured	Tri-axal accelerometer
Djuric 2011	US	40	52.2	100	26.6	Breast cancer	Self-reported	Validated questionnaire from the Women's Health Initiative
Dunn 2019	US	24	59	29		Ischeamic heart diseae	Device-measured	Actigraph GT9X accelerometer (Pensacola, FL USA)

Dwinger 2020	Germany	4283	67.3	56	28.9	Healthy	Self-reported	The Freiburg Questionnaire for Physical Activity (FFKA)
Elley 2003	New Zealand	878	57.9	67	30	Healthy	Self-reported	Self report questionnaire from the Auckland heart study
Ellingson 2019	US	91	41.7	53	29.6	Healthy	Device-measured	Accelerometer
Elliot 2007	US	599	41	3		Healthy	Self-reported	Self report questionnaire (healthy physical activity level score)
Ferrara 2020	US	394	32.5	100	29.4	Overweight/obesity and pregnant	Device-measured (for MVPA); Self-reported (for change of PA)	ActiGraph wGT3X-BT accelerometer (Pensacola, FL USA) and self-report questionnaire
Friederichs 2016	Netherlands	1989	44.7	69	26	Healthy	Self-reported	Dutch Short Questionnaire to Assess Health Enhancing PA (SQUASH
Frost 2004	US	24	66.1	75	29.9	Osteoarthritis	Self-reported	Modifiable Activity Questionnaire (MAQ)
Gale 2019	US	40	74.5	57		Demention	Self-reported	International Physical activity questionnaire (IPAQ)
Gianos E 2018	US	397	64.6	24	29.7	Coronary heart disease	Self-reported	Yale Physical Activity Survey (part 2)
Gilbert 2018	US	340	58.6	73	29.4	Knee osteoarthritis or rheumatoid arthritis	Device-measured	ActiGraph GT1M accelerometer (Pensacola, FL USA)
Gillham 2010	UK	52	68.3	29		Stroke	Self-reported	Self reported exercise frequency in interview
Gonzalez-Cutre 2020	Spain	40	45.2	78.1	≥40	severe obesity with sleeve gastrectomy	Device-measured	Actigraph GT3X accelerometers (Pensacola, FL)
Grischott 2023	Switzerland	149	51.1	50		Smoking	Self-reported	Self-report questionnaire
Groeneveld 2011	Netherlands	408	47.4	0	28.8	CVD risk	Self-reported	Short QUestionnaire to ASsess Health enhancing PA (SQUASH)
Haejung 2021	Korea	88	61.8	18.2		Heart failure	Self-reported	Korean Activity Scale/Index
Hardcastle 2013	UK	334	50.2	67	33.7	CVD risk	Self-reported	Short interview version of the International Physical Activity Questionnaire (IPAQ)
Havenar 2007	US	41	45.6	84	27.7	Healthy	Self-reported	Seven Day PA Recall (7 Day PAR)
Hollis 2015	Australia	54	47.3	100	25.1	Pre-menopausal obesity	Device-measured	Yamax SW200 Pedometers (Bridgenorth, UK)
Ismail 2020	UK	1742	69.8	15		CVD risk	Device-measured	ActiGraph GT3X accelerometer (Pensacola, FL USA)

Janssen 2013	Netherlands	210	57.7	19	28	Ischaemic coronary heart disease	Device-measured	Yamax Digiwalker SW-200 pedometers (Bridgenorth, UK)
Katz 2008	US	316		67		Healthy	Self-reported	The Physical Activity Readiness Questionnaire
Knittle 2015	Netherlands	78	62.8	67	27	Rheumatoid arthritis	Self-reported	Self-report postal questionnaires
Koelewijn-van Loon 2010	Netherlands	615	56.9	55	29	CVD risk	Self-reported	Validated questionnaire of self-reported lifestyle in physical activity
Kolt 2007	New Zealand	186	74.2	66		Healthy	Self-reported	Auckland Heart Study Physical Activity Questionnaire (AHSPAQ) REF
Lakerveld 2013	Netherlands	622	43.5	58	29	Type 2 diabetes and CVD risk	Self-reported	The Short QUestionnaire to ASsess Health-enhancing physical activity (SQUASH)
Larsen 2020	Denmark	70	71.9	60	27.2	Healthy	Self-reported	Garmin Vivofit 3 tri-axial accelerometer (
Lawton 2008	New Zealand	1089	58.9	100	27.2	Healthy	Self-reported	Self-reported physical activity questionnaire
Lilienthal 2014	Canada	86	64.5	66	26.8	Healthy	Self-reported	Modified Community Healthy Activities Model Program for Seniors Questionnaire (CHAMPS)
Lin 2016	Taiwan	76	63.5	100		Metabolic syndrome	Self-reported	Chinese version of the International Physical Activity Questionnaire (IPAQ)-short form, Chinese version
Lindeman 2020	US	72	20.7	69		Healthy	Self-reported	International Physical Activity Questionnaire – Short Form (IPAQ-SF)
Lion 2020	Luxembourg	25	45.7	100	24.4	Non metastatic cancer	Device-measured	ActiGraphTM GT3X+ Accelerometer (Pensacola, FL USA)
Lo 2021	Taiwan	43	64.4	48.8	25.4	Multimorbidity	Self-reported	The International Physical Activity Questionnaire (IPAQ)-Chinese version short form
MacKinnon 2010	US	369	40.7	2	27.7	Healthy	Self-reported	A survey of self-reported physical activity
Mahmood 2023*	US	74	71.1	10.6		Mild Cognitive Impairment	Device-measured	The Community Healthy Activities Model Program for Seniors (CHAMPS) physical activity questionnaire
Marcus 1998	US	194	44.3	76	28.4	Healthy	Self-reported	self-report instrument adapted from a seven-day physical activity recall questionnaire
Marcus 2007	US	239	44.5	82	28.5	Healthy	Self-reported	Physical Activity Recall (PAR) interview
Marques 2017	Portugal	99	48.1	98		Chronic fatigue	Self-reported	Short Questionnaire toAssess Health-enhancingPhysicalActivity(SQUASH)

Mascola 2009	US	26	43.8	92	35	Overweight/obesity	Self-reported	Stanford SevenDay Physical Activity Recall
Mose 2020	Denmark	98	44	68		Medication-overuse headache	Self-reported	Physical Activity Scale 2 (PAS 2.1)
Nooijen 2016	Netherlands	39	44	15	24.7	Subacute spinal cord injury	Device-measured	Ambulatory monitoring system with accelerometers
Nourizadeh 2020	Iran	70	28.5	100	30.3	Preconception with overweight or obesity	Self-reported	Iranian version of International Physical Activity Questionnaire (IPAQ) short form
O'Halloran 2016	Australia	30	82.6	84	23	Hip fracture	Device-measured	ActivPal Accelerometer(Glasgow, UK)
Pedersen 2019	Norway	202	42.5	24		Healthy	Self-reported	The International Physical Activity Index
Pellegrini 2022	USA	45	65.4	60	32.8	Total knee replacement	Device-measured	Actigraph Link GT9X Accelerometer (Pensacola, FL USA)
Quintiliani 2016	US	60	32.2	58		Healthy	Self-reported	Behavioral Risk Factor Surveillance Survey
Quintiliani 2021	USA	102	46.5	88	>25	Overweight	Device-measured	Actigraph wGT3X-BT accelerometer (Pensacola, FL USA)
Quirk 2012	Australia	23				Peripheral artery disease	Self-reported	The International Physical Activity Questionnaire (IPAQ)
Rausch-Osthoff 2017	Switzerland	42	68.2	50	25.8	Chronic obstructive pulmonary disease	Device-measured	SenseWear Pro armband accelerometer (BodyMedia, Inc., Pittsburgh, PA, USA)
Reid 2012	Canada	141	60.5	27	29.4	Acute coronary syndrome	Device-measured	Yamax DIGIWALKERTM Pedometer (Bridgewater, UK)
Reinhardt 2012	Australia	38	32.5	100	28.8	Previous gestational diabetes mellitus	Self-reported	The International Physical Activity Questionnaire (IPAQ) long version
Resnicow 2005	US	571	46.5	76		Healthy	Self-reported	An adaptation of the Community Healthy Activities Model Program for Seniors (CHAMPS) PA recall
Scales 1998	US	61	59.6	29	27.4	Coronary artery disease	Self-reported	The modified Physical Activity Recall
Scott 2019	UK	35	59.3	43	31.5	Chronic health conditions	Device-measured	Pedometer and the Community Healthy Activities Model Programme for Seniors Scale (CHAMPS)
Selcuk-Tosun 2019	Turkey	70	50.5	66	36.3	Type 2 diabetes	Self-reported	OmronHJ - 321 - E Pedometers (Kyoto, Japan)
Sheppard 2016	US	31	54.7	100	37.5	Breast cancer	Self-reported	International Physical Activity Questionnaire Short Form (IPAQ-SF)
Simpson 2015	UK	166	49.9	84	34.3	Obesity	Self-reported	IPAQ (International Physical Activity Questionnaire)
Turner 2016	US	64	53.1	36	29.1	Multiple sclerosis	Self-reported	The Godin Leisure-Time Exercise Questionnaire

Tuvemo 2020	Sweden	114	83	70		Requiring walking aids or home help services	Self-reported	The Frändin–Grimby Activity Scale
Valeiro 2022	Spain	46	66	25.6	27.3	COPD	Device-measured	Dynaport accelerometer (McRoberts BV, The Hague, The Netherlands)
Van Keulen 2011	Netherlands	1224	57.1	45	27.4	Hypertension	Device-measured	The modified Community Healthy Activities Model Program for Seniors PA questionnaire
vanBakel 2022	Netherlands	212	63	23	27.1	Coronary artery disease	Device-measured	ActivPAL3TMmicro Accelerometer (PAL Technologies Ltd., Glasgow, United Kingdom)
Vlaar 2017	Netherlands	642	44.8	52	27.5	Type 2 diabetes	Self-reported	Short Questionnaire to Assess Health-Enhancing Physical Activity, supplemented with culturally specific activities
Weinstock 2019	US	66	34.8	61	27.1	Alcohol use disorder	Self-reported	The Timeline Followback (TLFB)
West 2016	US	398	48.4	90	36	Overweight/obesity	Self-reported	Self-monitoring journal submissions
Whitehead 2022*	US	214		90.7		Healthy	Self-reported	Weekly Leisure-Time Exercise Questionnaire (WLTEQ)
Young 2019	US	67	61.2	61	31.9	Prediabetes or diabetes	Device-measured	Actigraph 7185 accelerometers (Pensacola, FL UK)

*Study eligible for inclusion but not included in meta-analyses

Supplementary Table 3: Intervention characteristics

Study ID	Intervention content	Control content	MI provider	Training for MI provider	MI Fidelity	Duration (Month)	Personal contact in MI delivery	Mode of MI delivery	No. of MI session	Duration each MI session
Albright 2014	A condition-specific tailored website (tailored mom-centric PA "resource directories" and newsletters) + telephone calls with a counsellor using MI + a pedometer to track and set goals using steps	A condition-specific website and resources on how to increase PA	-	-	Fidelity to the key intervention components (e.g., goal setting, barrier resolution, resources) was 88%. Setting the woman's next MVPA goal was discussed in 100% of the evaluated calls.	12	Individual	Telephone	17	12.7
Alothman 2022	MI sessions containing different components and topics of a healthy lifestyle	Brief advice to promote a healthy lifestyle delivered by different therapists. The therapist firmly and clearly discussed general lifestyle health topics and provided educational materials during each session.'	Therapists	MI training provided by a certified lifestyle medicine specialist on intervention delivery using a training manual, role play and didactic instruction	-	0.75	Individual	Web-based	6	30
Anderson 2014	One to one MI consultations to improve diet and physical activity	Weight loss booklet	Trained lifestyle counsellors	-	-	12	Individual	Combination of in-person and remote delivery	12	26.3
Anderson 2018	Personalised advice on dietary intake and a graduated approach aimed at increasing activity + consultations (self-identification, goal	Lifestyle booklet	Lifestyle coaches (with a nursing background)	Bespoke training on the delivery of the intervention programme	62% of the intervention components were delivered as per protocol. LCs assessed perceived patient engagement, receptivity and motivation highly:	3	Individual	Combination of in-person and remote delivery	5	25

	setting, etc.)+ a ‘LivingWELL’ information pack + pedometer and walking programme + MI techniques used to explore self-assessed confidence to change and self-perceived benefits				89%, 92%, and 89% respectively, in Likert-scaled questions					
Ang 2013	Aerobic exercise prescription and individualized supervised exercise session + MI telephone calls to improve exercise	Aerobic exercise prescription and individualized supervised exercise sessions + didactic health information on topics of : overview of FM; pain; fatigue; sleep; stress; and living well with FM	MI-trained health practitioners	-	High score	3	Individual	Telephone	6	-
Arbillaga-Etxarri 2018	Interview including MI and stage-matched approach to eliciting behavioural change + a dossier containing maps of walking trails with explanation from physiotherapist + pedometer and a personalised calendar to monitor their physical activity + ELF information brochure and the link to project website + walking group accompanied by an experienced physical activity trainer	Usual standardised pharmacological and/or non-pharmacological treatment for COPD, including pulmonary rehabilitation at the discretion of their physician + general health counselling and the European Lung Foundation (ELF) information brochure “Living an active life with COPD”	Respiratory physiotherapist	Adequately trained in behavioural strategies	-	12	Individual	Combination of in-person and remote delivery	5	18

Asvat 2014	MI sessions to improve physical activity	Sessions including reviewing BMI, physical activity and nutrition, prescribing specific lifestyle modifications and providing general information on stress and cancer.	Interventionist	16 hours of MI training with an experienced MI trainer + several hours of self-training in MI by reviewing MI book and watching the videotape MI training series	An average of 4 on Global Scores and Global Spirit Rating (5-point scale) represents the threshold for competency in MI	1	Individual	Combination of in-person and remote delivery	3	40
Aunger 2020	Behaviour change intervention based on Self-Determination Theory, comprising multiple behaviour change techniques, such as social support (MI and emotional), individual feedback on sedentary behaviour and physical activity, etc.	Regular orthopaedic care including physiotherapy post-operation	Researcher	-	Overall fidelity of treatment delivery in the study was competent, with a mean of 3.1 (out of 5), indicating adequate standard of delivery of the study. The mean of the independently rated and self-rated scores for supporting basic psychological needs during the MI session was 2.7 (out of 5), indicating that this aspect was sub-optimally delivered.	1.5	Individual	Combination of in-person and remote delivery	4	33.8
Baig 2013	A curriculum (group classes)based on self-determination theory including emphasis on the importance of intrinsic motivation of behaviour change, information about diabetes, nutrition and physical activity, cognitive approach to behavioural problem solving, photovoice	One lecture on diabetes self-management by a bilingual chronic disease health educator	Lay leaders identified from the church community to lead the classes	Three 3-hour trainings that included training on program content and process, with emphasis on acquisition of coaching skills through modelling, role play, and feedback. They taught mock lessons, facilitated	-	2	Group	In person	8	90

	exercise encouraging the use of photographs and storytelling, etc.			group discussions, and demonstrated their skill in coaching and patient-centred communication.						
Barrett 2018	An education session (same as the control group) + a MI-CBT intervention exploring participants' feelings about change and evoking intentions to change, identifying drivers of ambivalence and resistance and helping to form goal-directed action plans	An education session based around self-management and lifestyle modification and using a self-determination theory (SDT) framework to support, educate and motivate participants around positive lifestyle choices	Experienced allied health clinician	Trained in MI-CBT, including workshop attendances, and one-on-one coaching from an experienced practicing psychologist.	-	3	Individual	Telephone	8	30
Becker 2008	Counselling about the guideline + encouragement to use specifically designed brochures on motivational and behaviour change and posters to communicate the key messages	Guideline via mail	Nurses	A 20-hour training (2 full-day workshops and 1–3 supervision sessions) in using the LBP guideline of the DEGAM and introducing to motivational counselling strategies to increase skills to motivate LBP patients for regular physical activity.	-	6	Individual	In person	3	12.5
Befort 2008	Culturally-targeted behavioural weight loss program (individual calorie and fat gram goal setting and self-monitoring logs of weight, food	Health education using handouts and flip-charts on four topics from six options: breast, colon, or cervical cancer screening, smoking cessation, helping	Advanced doctoral clinical psychology student	Extensively trained and supervised by a doctoral-level clinical psychologist. Training elements included reading the seminal text on the	-	4	Individual	Combination of in-person and remote delivery	4	30

	and physical activity) + MI session with counsellor	others quit smoking and improving sleep		topic, watching MI training videotapes, participating in a 2-day training session, and conducting simulated counselling sessions.						
Bennett 2007	Tailored MI counselling session including summarizing, feedback, affirmation, build-up of participant's self-efficacy or confidence, identifying barriers, and etc. + a pedometer to encourage walking	Telephone calls from the physical activity counsellor to set times for measurement appointments and for brief social conversations	Master's-prepared research assistant	8 hours of group training and 6 hours of individual training by an experienced MI trainer	-	6	Individual	Combinatio n of in-person and remote delivery	3	23.3
Bennett 2008	Telephone call, including guidelines for safety, a discussion of perceived exertion in terms of maintaining moderate levels of exercise, tailored MI content on barriers, goal setting, solving problems, offering encouragement, and reformulating goals	Telephone call about their physical activity using a prescribed set of five questions in a script with no MI content	A master's-prepared research assistant	16 hours of MI training from a certified trainer in preparation for providing an MI intervention	-	6	Individual	Telephone	6	17.5
Benzo 2016	MI based health coaching plus a written action plan for exacerbations (the use of antibiotics and oral steroids) and brief exercise advice	Care in accordance with the Global Initiative for Chronic Obstructive Lung Disease and referral for conventional pulmonary rehabilitation	Nurse or therapist	-	-	1	Combinati on of individual and group	Combinatio n of in-person and remote delivery	4	90

Berlant 2004	Standard WALKING project intervention + MI telephone call to build the motivation for change through the appliance of the five general principle + a brief intervention for smoking	Standard WALKING project intervention + check in calls with unstructured prompts to adhere to physical activity prescription	Advanced graduate student and intern	Supervised by a licensed psychologist and trained in using MI, training consisted of 5.5 h of video training and 5 h didactic group training	98% of the MI matched the protocol	3	Individual	Telephone	5	-
Blackford 2016	Email and telephone support with MI techniques to improve diet, physical activity, and healthy weight maintenance + printed and online material including booklet, exercise charts, resistance band, nutrition panel wallet card, and a website with progress tracker and interactive blog	Waiting list	Research assistants	Trained in motivational interviewing techniques	-	6	Individual	Telephone	6	-
Bombardier 2008	Motivational interview and goal-setting meeting to build motivation to change + a short letter to affirm the participant's strengths and motivation+ telephone counselling sessions to promote follow-through with the plan	Waiting list	Research care manager	A standard 2-day training program in motivational interviewing and additional training plus ongoing supervision from a clinical psychologist experienced in motivational interviewing	-	3	Individual	Combination of in-person and remote delivery	6	37.5
Bombardier 2013	Motivational interview and goal-setting meeting to build motivation to change	Waiting list	Master's-level counsellors	A standard 2- to 3-day training program in MI and additional training	Overall fidelity to MI-consistent behaviours and spirit was good—72% of questions were	3	Individual	Combination of in-person and	9	32.5

	+ a short letter to affirm the participant's strengths and motivation+ telephone counselling sessions to promote follow-through with the plan			plus on-going supervision from a clinical psychologist and experienced MI trainer.	open rather than closed, and the ratio of reflections to questions was 2.9:1. The mean (SD) frequency of observed therapist behaviours that were MI inconsistent was 0.26 (0.57) per session. The mean (SD) frequency of observed client-resistive behaviour was similarly rare, 0.28 (0.76) occurrences per session. Ratings of MI spirit were satisfactory (means 5.73–5.88; range 4–7)			remote delivery		
Brodie 2005 (MI and others)	Standard care + motivational interviewing programme including problem solving, practice of behavioural change strategies and discussion	Standard care including directed provision of information and recommendations to increase physical activity	Researcher	-	-	5	Individual	In person	8	60
Brodie 2005 (MI only)	Motivational interviewing programme including problem solving, practice of behavioural change strategies and discussion	Standard care including directed provision of information and recommendations to increase physical activity	Researcher	-	-	5	Individual	In person	8	60
Brown 2015	Culturally-sensitive self-help materials + motivational interviewing calls to discuss BP medication adherence, eating	Skin cancer awareness materials or sunblock to maintain contact	Project personnel	Trained in motivational interviewing and the SHARE intervention	-	12	Individual	Telephone	5	-

	more fruits and vegetables and less sodium, and increasing physical activity + two tailored newsletters + a workshop									
Burtin 2015	Physical activity counselling program, consisting of eight individual sessions focusing on patients' motivation and confidence of change physical activity, evaluation of physical activity and feedback.	Duration and timing of the individualized sessions were similar to the intervention group, but the general health status of the patient and the progression during training was discussed during the conversations. Intermediate evaluation of physical activity was performed, but no structured feedback was provided	Research assistants (Master in Science in Physiotherapy)	Exercise physiology background and specific expertise in respiratory physiotherapy + three individual training sessions of 60 minutes in the principles of MI were organised by an experienced health psychologist (FD).	-	6	Individual	In person	8	25
Celano 2018	MI session including goal setting, feedback, assessment of physical activity and advice on the benefit of physical activity + positive Psychology strategy which required patients to remember small, daily successes	Positive psychology strategy	-	-	-	9.5	Individual	Telephone	9.5	-
Celano 2020	The PP-MI intervention focusing on both PP (Positive psychology) exercises and MI with systematic goal-setting related to	Regular clinical care for HF and other medical illnesses	Study interventionist(researcher)	-	-	3	Individual	Telephone	12	-

	physical activity, diet, and/or medications									
Collins 2019	Standard educational print material on risk factor modification for CVD + text messages + brief MI phone call	Standard educational print material on risk factor modification for CVD	Bachelor's level research assistant	A two-day training workshop was provided by an internationally recognized expert in MI	-	3	Individual	Telephone	4	20
Conn 2003 (MI and others)	Motivational intervention including consciousness-raising, self-re-evaluation and guided problem solving to increase exercise	Standard educational information emphasizing exercise benefits and appropriate exercise	-	-	-	3	Combination of individual and group	Combination of in-person and remote delivery	3	-
Conn 2003 (MI only)	Motivational intervention + prompting (telephone calls and mailed materials of exercise-related information)	Standard educational information emphasizing exercise benefits and appropriate exercise	-	-	-	3	Combination of individual and group	In person	3	-
Coumans 2022	MyLifestyleCoach is a web-based computer tailoring intervention that consists of a general opening session, followed by a diet module to promote dietary behaviour and a previously tested PA module to improve PA levels of Dutch adults. This intervention is based on principles of SDT and uses counselling techniques derived from MI.	Waiting list	-	-	-	3	Individual	Web-based	5	30

De Vries 2016	Coaching using MI technique to improve physical activity	Usual care physical therapy	Geriatric physical therapists	2 days training in Coach2Move strategy	-	6	Individual	In person	10	90
Dennett 2018	Motivational interviewing sessions for increasing general movement before increasing the amount of moderate-intensity activity	Individualized exercise + group education + a video or written home exercise program + diary to encourage exercise outside the program and monitor exercise adherence	Physiotherapist	-	-	1.75	Individual	Telephone	7	21
Djuric 2011	Written educational materials + telephone counselling using motivational interviewing + pedometers, daily food and exercise log + fast tracker + fast food booklet + fat gram counter/food exchange list book and example menus at individually appropriate calorie levels	“My Pyramid” plan including recommendations for daily exercise and dietary guidelines +“Choices for Good Health” and “Cooking Smart” brochures + pedometers and study newsletters	Registered dietitian	Trained in motivational interviewing techniques	-	12	Individual	Telephone	19	-
Dunn 2019 (MI and others)	Motivational interview to encourage PA based on instructions provided by the hospital staff and social support text messages from nurses + social support text messages from their self-identified significant other	No details on control	Nurses	Online and in-person training from an experienced motivational interviewer	Audio fidelity to the protocol was confirmed by the review of randomly selected recordings from 25% of the sessions. Audio reviews included the transcription and coding of the content by a trained research assistant, with each review being repeated by the principle	1.5	Individual	In person	1	60

					investigator to confirm accuracy.					
Dunn 2019 (MI only)	Motivational interview to encourage PA based on instructions provided by the hospital staff and social support text messages from nurses	No details on control	Nurses	Online and in-person training from an experienced motivational interviewer	Audio fidelity to the protocol was confirmed by the review of randomly selected recordings from 25% of the sessions. Audio reviews included the transcription and coding of the content by a trained research assistant, with each review being repeated by the principal investigator to confirm accuracy.	1.5	Individual	In person	1	60
Dwinger 2020	Telephone based health coaching using MI to change health behaviours	No coaching	Nurses	Trained in TBHC with MI and SDM components by experts directly trained by Health Dialog + supervised two to four times per year by two experienced supervisors	-	12	Individual	Telephone	8	-
Elley 2003	Prompt card + consultation to discuss increasing physical activity + MI telephone calls + newsletters about physical activity initiatives	Usual care + waiting list	Primary care clinicians	Four hours of training in how to use motivational interviewing techniques to give advice on physical activity and the green prescription	-	3	Individual	Combination of in-person and remote delivery	4	-
Ellingson 2019	Fitbit Charge wrist-worn activity monitor + discussion of habits and goals + habit education + brief	Fitbit Charge wrist-worn activity monitor	Staff member	Staff was trained	-	2	Individual	Combination of in-person and remote delivery	3	-

	conversation regarding experience, self-selected PA goals and motivation for change								
Elliot 2007	MI counselling to promote behaviour change + phone calls or/and additional meetings	No treatment	Counsellors	Approximately 90 hours of MI training, including seminars, educational videotapes, personal coaching from an expert trainer, and practice with standardized patients	Reflections-to-questions ratio of 2.2 and 74% of reflections were complex (criteria for competency are a ratio greater than 1 and more than 40% complex reflections).	10	Individual	Combination of in-person and remote delivery	7	60
Ferrara 2020	The intervention targeted behaviour changes for weight management, healthy eating, physical activity and stress management using MI techniques and a step-wise, phased approach based on social cognitive theory and the transtheoretical model	Standard KPNC antenatal medical care, which included an antenatal visit at 7–10 weeks' gestation, an additional seven antenatal visits on average, and periodic health education newsletters, including the Institute of Medicine GWG guidelines and information on healthy eating and physical activity in pregnancy. In addition to standard KPNC antenatal care, women in the usual care group received four study newsletters that focused on women's health and safety	Dietitians	-	-	3.25	Individual	Combination of in-person and remote delivery	13	-

		during pregnancy without addressing GWG.							
Friederichs 2016	Web-based computer tailored PA intervention based on the theoretical insights of SDT and the practical applications of MI: automated text-based sessions with tailored feedback text, motivational dialogue and short videos	Waitlist control- no intervention during study period (only research measures) had access to iMove intervention after study ended	-	-	-	3	Individual	Web-based	4	-
Frost 2004	Exercise Diary+ motivational exercise counselling session + telephone booster contacts	Exercise Diary + telephone calls to remind subjects to mail in their Exercise Diary sheets and to answer any questions about completing the Exercise Diary	Researcher	Training in all aspects of the research protocol and was supervised by the clinical psychologist	-	2	Individual	Combination of in-person and remote delivery	4	31.9
Gale 2019	Standardized educational materials + counselling including goal setting, feedback and problem-solving coach to improve physical activity, adherence to a Mediterranean diet, and social/cognitive stimulation	Standardized educational handout + routine care	Health coach	Training on the evidence-based lifestyle factors that influence cognitive decline and techniques that facilitate behavioural change	-	6	Individual	Combination of in-person and remote delivery	28	18.5
Gianos E 2018	Behavioural program including telephone-based MINT sessions with tailored daily text messages + usual care	Usual care + cardiovascular procedure with education + medication adjustment	Health educator	Trained by a member of the MINT network	-	6	Individual	Telephone	9	30

Gilbert 2018	Brief physician counselling as control group + motivational interviewing intervention to promote physical activity	Counselling with physician including discussion and laminated card of physical activity recommendations and encouraging participants to work toward or maintain physical activity	Physical activity advocates	Training with a member of the Motivational Interviewing Network of Trainers	-	24	Individual	Combination of in-person and remote delivery	6	19.2
Gillham 2010	Enhanced secondary prevention, comprising information about stroke pathology, explanation of individual stroke risk factors, and a motivational interviewing style of discussion about behaviour change intentions, with development of a plan for behaviour change + telephone support and follow-up to discuss progress	No intervention	-	-	-	1.5	Individual	In person	1	-
Gonzalez-Cutre 2020	Physical training sessions with activities focused on working physical capabilities with machines (e.g., cardiorespiratory fitness); and innovative sessions to enhance psychosocial aspects among participants (e.g., body expression). The	General recommendations of PA from their doctors as part of their usual care, which were focused on trying to maintain an active lifestyle after surgery, but without giving specific information regarding the type, frequency,	Sport sciences professionals	Standardized training in the application of SDT-based motivational strategies, consisting of a 10-h theoretical and practical seminar about SDT-based motivational strategies in	-	6	Individual	In person	70	75

	SDT-based motivational strategies were applied in both types of sessions.	duration or intensity of the physical activities.		exercise and 2-month practice in a real-world setting, where an external observer with expertise in SDT rated their agreement with the strategies using an observation sheet						
Grischott 2023	Health coaching with MI which included deciding unhealthy behaviours or risk factors they wanted to address and support to achieve beneficial changes	Patients received smoking cessation counselling according to' Free of Tobacco (state of the art smoking cessation counselling)	GP	5 training sessions from unvarying members of the study team with extensive experience in HC or FoT	-	12	Individual	In person	4	-
Groeneveld 2011	Telephone counselling using MI and aiming at PA and diet, or smoking + brochures containing information on PA, healthy eating, smoking cessation, and CVD	Usual care, consisting of brief oral or written information from the occupational physician about their risk profile, based on the periodical health screening results + brochures containing information on PA, healthy eating, smoking cessation, and CVD	-	-	-	6	Individual	Combination of in-person and remote delivery	7	35.4
Haejung 2021	MI sessions including discussion of participants' perceived importance of behaviour changes, severity of unhealthy behaviours, barriers to and benefits of healthy behaviours, and self-efficacy	Usual care from a physician at an outpatient clinic	One doctoral student and one master's student	Training of the theoretical basis of MI in a graduate program in nursing, MI practice through role play, and advice from a researcher who had experience conducting and researching MI	Treatment fidelity was ensured by regular debriefing meetings and rating of a random sample of 10.0% of the intervention recordings	1	Individual	Telephone	8	15

Hardcastle 2013	Counselling sessions using MI strategies integrated with a stage-matched approach + standard leaflet providing information on exercise and nutrition.	Standard leaflet providing information on exercise and nutrition.	Physical activity specialist and registered dietician	Two four-hour training sessions conducted by the first author	-	6	Individual	Combination of in-person and remote delivery	5	25
Havenar 2007	Face-to-face sessions, emails and phone calls using MI to change physical activity	Standard health care information from the American College of Sports Medicine: in-person session and email covering different topics and phone call consisting of the counsellor reviewing material from the previous email	Researcher	Training in client-centred therapy and counselling + MI training via written and video materials as well as non-training related correspondence with a MINT certified trainer.	-	3	Individual	Combination of in-person and remote delivery	11	35.7
Hollis 2015	Same materials as control group + consultations with principles of MI	Written individualised advice through the post to promote change in diet and physical activity, consisting of analysis of their food and physical activity record, tailored weight goals, dietary and physical activity behaviour change strategies, weight tracking sheet, menstrual calendar diary, and a food and physical activity diary and weight control pamphlet	Accredited Practising Dietitian and exercise physiologist	-	-	12	Individual	In person	5	60
Ismail 2020 (group delivery)	Motivational interviewing session focusing on physical	Referrals to locally commissioned community-based	Health trainer	8 weeks of didactic learning, role-playing, group	All sessions were audiotaped and competency was	12	Group	In person	10	120

	activity and diet delivered in group + workbook, key learning points for MI session, action planning worksheets, case studies, self-monitoring diaries and a pedometer	weight loss, smoking cessation and/or exercise programmes		exercises and case discussions using standardised materials on motivational interviewing and behaviour change techniques drawn from cognitive behaviour therapy	monitored and supervised weekly by the clinical psychologist. Fidelity to the manual consisted of the health trainer recording targets set and achieved per session.					
Ismail 2020 (individual delivery)	Motivational interviewing session focusing on physical activity and diet delivered individually + workbook, key learning points for MI session, action planning worksheets, case studies, self-monitoring diaries and a pedometer	Referrals to locally commissioned community-based weight loss, smoking cessation and/or exercise programmes	Health trainer	8 weeks of didactic learning, role-playing, group exercises and case discussions using standardised materials on motivational interviewing and behaviour change techniques drawn from cognitive behaviour therapy	All sessions were audiotaped and competency was monitored and supervised weekly by the clinical psychologist. Fidelity to the manual consisted of the health trainer recording targets set and achieved per session.	12	Individual	In person	10	40
Janssen 2013	Outpatient CR programme + self-regulation programme focused on maintenance of lifestyle change including motivational counselling session, group sessions (structured around the self-regulatory phases of goal pursuit), and follow-up sessions + standard care	Outpatient CR programme + individual interview encouraging participants to set a salient personal health goal + standard care consisting of regular follow-up appointments with cardiologist	Health psychologist	-	-	6	Individual	In person	1	60
Katz 2008	Treatment from physicians trained in	Treatment from physicians who took	Physicians	Controlled educational	-	6	Individual	In person	5	-

	Pressure System Model behavioural counselling methods	part in their usual residency curriculum		intervention aiming to engage first year internal medical resident's PA counselling skills						
Knittle 2015	Same educational session as control group + one-to-one MI to improve physical activity + exercise diary + one to-one self-regulation coaching session + follow-up phone call discussing self-regulating physical activity	Small group educational session including information about the importance of PA for people with RA, PA guidelines, and resolved myths surrounding PA and RA	Physical therapists	Training in the delivery of MI	-	1.25	Individual	In person	1	45
Koelewijn-van Loon 2010	Consultations and follow-up telephone call including risk assessment, risk communication, distribution of a decision aid and motivational interviewing	Consultation including risk profile and usual care	Practice nurses	Two-day training outside the practice	-	1	Individual	Combination of in-person and remote delivery	1	10
Kolt 2007	TeleWalk intervention comprising telephone counselling sessions based on the appropriate stage of change for individual to adoption of physical activity (including MI technique) and supplementary material of a walking log and pamphlets	No intervention	Counsellor	Counsellor was trained	-	3	Individual	Telephone	8	12.5

Lakerveld 2013	Counselling sessions + motivational interviewing + problem solving treatment to changes lifestyle behaviour	Brochures containing health guidelines regarding physical activity, a healthy diet, and smoking cession (if appliable)	Practice nurses	Training from experienced psychologists	-	12	Individual	Combination of in-person and remote delivery	9	30
Larsen 2020	Physical activity monitor + MI intervention (telephone calls MI counselling	Physical activity monitor (PAM) for everyday use + a pamphlet with the national recommendations on PA in aging population	Physiotherapists	Training and education in the MI approach to telephone-based health behaviour counselling	Motivational Interviewing Treatment Integrity Scale version 4 (MITI 4)	3	Individual	Telephone	7	20
Lawton 2008	The green prescription including briefly counsels using motivational interviewing techniques to increase physical activity, a written exercise advice on a “green script” given to the patient + follow-up telephone calls and visit	Usual care from primary care practice	Primary care nurse	-	-	9	Individual	Combination of in-person and remote delivery	6	30
Lilienthal 2014	Telephone-based MI sessions	Healthy activity guide	Clinical psychology doctoral candidate	Previous training and experience in MI	-	1	Individual	Telephone	4	50
Lin 2016	Same counselling session and educational brochure as control group + individualized lifestyle modification program focusing on physical activity promotion by telephone-delivered motivational interviewing	Individual brief lifestyle modification counselling session + educational brochure including information on MetS and standard management of MetS based on the guidelines of NCEP-ATP III and ACSM, including diet control, adequate level	Nurse/researcher	10-year experience in both metabolic control and physical activity training	-	3	Individual	Telephone	12	22.5

		of physical activity, and stress coping								
Lindeman 2020	Incentive as the control group + MI Sessions to increase physical activity	Incentive- introduced to a course with requirement to exercise where engagement in physical activity was worth of course grade	Graduate students	At least one graduate level course on Motivational Interviewing and a two-hour training to review the core concepts and techniques	Client Evaluation of Motivational and Therapist Evaluation of Motivational Interview scores	3	Individual	In person	3	30
Lion 2020	MI counselling to promote physical activity	Standard care	Research assistants	MI training of a 3-day workshop (21 hours) on MI delivered by a certified MI trainer	-	3	Individual	Combination of in-person and remote delivery	12	32.1
Lo 2021 (MI and others)	Evaluation of participants' medical history and lifestyle + coached individual exercise training + Borg scale to assess exercise intensity + monitoring to ensure safety + face to face MI + telephone MI session	Usual care: general recommendations for physical activity	Trained nurse	-	Fidelity of intervention delivery was ensured by combining different intervention components	3	Individual	Combination of in-person and remote delivery	24	22.5
Lo 2021 (MI only)	Telephone MI sessions	Usual care: general recommendations for physical activity	Trained nurse	-	Fidelity of intervention delivery was ensured by combining different intervention components	3	Individual	Telephone	12	22.5
MacKinnon 2010	MI counselling to promote physical activity	Brief explanations of their test results (anthropometric and aerobic fitness measures) and encouragement of taking their own	Counsellor	Previous experiences in MI	-	24	Individual	Combination of in-person and remote delivery	12	60

		initiative to alter their lifestyles								
Mahmood 2023	CCT techniques designed to help patients manage problems with memory, attention, and executive functions + mindfulness-based stress reduction practice + brief motivational interviewing techniques to increase engagement in healthy lifestyle behaviours	Intervention with the same frequency focusing on setting and achieving short or long-term goals which included components of empathy and non-directive reinforcement of health, coping, and symptom management behaviours	-	Training of theoretical basis of MI in a graduate program in nursing, MI practice through role play, and advice from a researcher who had experience conducting and researching MI	-	2	Individual	In person	8	120
Marcus 1998	Individually-tailored reports generated by a computer expert system including pre-planned counselling messages + self-help manuals matched to the participant's stage of motivational readiness for physical activity adoption	Self-help booklets promoting physical activity	PhD level psychologists	Previous experiences in health behaviour change	-	6	Individual	Web-based	4	-
Marcus 2007 (telephone)	Individually tailored messages based on the transtheoretical model and social cognitive theory, stage-targeted booklets, and physical activity related tip sheets + telephone counselling	Mailed health education information of physical activity + waiting list	Health educator	-	-	12	Individual	Telephone	14	12.5

Marcus 2007 (web-based)	Individually tailored messages based on the transtheoretical model and social cognitive theory, stage-targeted booklets, and physical activity related tip sheets + manuals matched to their stage of motivational readiness for physical activity adoption and tip sheets	Mailed health education information of physical activity + waiting list	Health educator	-	-	12	Individual	Web-based	14	
Marques 2017	A brief self-regulation (SR)-based intervention to promote physical activity including individual motivational interviewing sessions, informational booklet, SR-based workbook, brief SR-based telephone counselling sessions, pedometer and a leaflet for their partner or significant other with relevant information	Standard medical care + flyer with information about the general health benefits, guideline and personal goal of physical activity	Health psychologist	Training in motivational interviewing	-	3	Individual	In person	2	60
Mascola 2009	A counselling adapted from the motivational interviewing (MI) model with procedures adapted from a cognitive behavioural therapy model of enhancing self-regulation +	Encouraged to select personal health goals and use resources available in their health-care plan and community to achieve their goals	Non-specialist health consultant	Training in motivational interviewing	-	3	Individual	Combination of in-person and remote delivery	7	-

	feedback from a health assessment conveying the importance of physical activity independent of weight status									
Mose 2020	Educational programme helping to clarify possible ambivalences with respect to behavioural changes and become aware of their own motivation for change+ “take home message” for further reflection	Outpatient standard procedures	Nurse and physiotherapist	Previous experiences in MI	-	3	Combination of individual and group	In person	6	75
Nooijen 2016	Individual sessions including MI	Regular rehabilitation	Coach	Training in motivational interviewing	-	8	Individual	In person	13	60
Nourizadeh 2020	MI sessions + routine care	Routine preconception care	Counsellor	Training in motivational interviewing	-	1.5	Group	In person	6	75
O'Halloran 2016	In addition, the intervention group completed a telephone-based motivational interviewing intervention,12,20 provided in eight 30-minute sessions delivered weekly.	Received usual care in the community during the trial. This may have involved participants attending their general practitioner or a community physiotherapist when required.	Physiotherapist	Training in motivational interviewing including a two-day workshop, online training and one-on-one coaching from an experienced motivational interviewing practitioner	The physiotherapist’s proficiency in using motivational interviewing was rated as competent on the global clinician rating at both assessments. There was improvement on two of counts (a percentage of open questions and reflection to question ratio) from beginning proficiency to competency at the midpoint of the intervention.	2	Individual	Telephone	8	30

Pedersen 2019	Sessions of group-based intervention elements + workshops + PA support group meetings (the intervention environment was drawn from a model combining the tenets of SDT with techniques from motivational interviewing)	Advised to follow the individual recommendations + waiting list	Physiotherapists	Training and feedback on how to facilitate the group workshops and provide participants with autonomy support, structure, and interpersonal involvement	The fidelity of intervention implementation, defined as the percentage of all sessions in all clusters carried out according to plan, was 94%	4	Group	In person	6	75
Pellegrini 2022	Enhanced physical activity intervention including a goal setting of aerobic activity + MI to help participants set a SMART goal related to aerobic activity, resolve barriers that may arise, and identify strategies that could be used to reach that goal	Standard outpatient physical therapy	Physical therapists	a brief training on techniques aligned with the principles of motivational interviewing from the principal investigator	Enhanced physical activity intervention sessions were rated significantly higher on implementation of intervention content as compared to control sessions (20.1 [6.0] vs. 1.5 [3.8], p<.001) in fidelity check	1.5	Individual	In person	12	-
Quintiliani 2016	Same tailored report as control group + telephone motivational interviewing-based counselling sessions	Tailored report presenting their baseline levels of diet and physical activity behaviours, recommended levels of these behaviours, brief bulleted tips and links to health-related websites	Peer counsellors	-	-	2	Individual	Telephone	3	-
Quintiliani 2021	A printed 2-page report including current levels of six behaviours, national guidelines and	The same tailored feedback report	Community health workers	Four 1–2 h training sessions including the review of MI strategies and study-specific	-	3	Individual	Telephone	12	60

	tips/information to achieve these behaviours + counselling session including feedback for baseline survey, tailored suggestions using MI			protocols + printed resources + role-playing exercises + evaluation call with a volunteer unknown to the community health worker trainee						
Quirk 2012	MI session that focused on the primary outcome of health behaviours, barrier and motivation to change, establishing goals for health behavioural change, elicit change talk, importance and confidence to change	No details on control	Trained psychologist	-	-	3	Individual	In person	4	60
Rausch-Osthoff 2017	Usual 12-week pulmonary rehabilitation (PR) programme + counselling sessions including MI	Usual 12-week pulmonary rehabilitation (PR) programme	Physiotherapists (MSc level)	Trained by an experienced MI-trainer and member of the MINT (Motivational Interviewing Network of Trainers)	-	3	Individual	In person	5	30
Reid 2012	Intervention including gaining commitment, identifying valued outcomes, setting goals, action planning, self-monitoring, identifying opportunities for physical activity, problem solving to overcome barriers, feedback and encouragement	No intervention	Physiotherapists	2 days of training and regular case discussions to maintain skills over time	-	12	Individual	Combination of in-person and remote delivery	9	-

Reinhardt 2012	Phone-based motivational interviewing program + usual care	Usual care	Diabetes educators	Previous experiences in MI	-	6	Individual	Telephone	10	20
Resnicow 2005	Culturally tailored self-help nutrition and PA intervention materials + telephone counselling calls based on MI	Standard nutrition and PA intervention materials	Master's- or doctoral-level psychologists	Approximately 16 hr of initial training and 12 hr of ongoing individual/group supervision	-	10	Individual	Telephone	4	-
Scales 1998	Same interventions as control treatment + stage-matched approach to lifestyle change including motivational interview and skills-based counselling sessions	Supervised exercise sessions + didactic lectures with group discussion on topics related to heart disease + optional behavioural interventions to change lifestyle including personal feedback, cooking demonstrations, and classes in smoking cessation, weight control and stress management	Interviewers	Training on how to conduct the interviews and pilot their interviews	-	1.75	Individual	In person	1	60
Scott 2019	The MI-CB intervention including MI counselling and a toolkit of tailored CB techniques	Discounted gym membership or exercise classes by the leisure centre	Researcher	Six MI workshops facilitated by a trainer from the MI Network of Trainers (MINT), which included theory and audio recorded supervised role plays	Competence was assessed using the MI Treatment Integrity (MITI) form (v 3.1.1)20 by the trainer. Six supervisions post-training were received where feedback was provided on audio-recorded sessions.	3	Individual	Combination of in-person and remote delivery	7	33.2
Selcuk-Tosun 2019	The TTM-based motivational interviews to promote physical exercise, adequate and proper	Usual care including diagnosis tests and medication treatment	Researcher	A two- stage motivational interview technique course (9 hours per stage)	-	6	Individual	In person	9	37.5

	nutrition and medication use targeted for behavioural change + follow-up table for medication use, walking and food consumption									
Sheppard 2016	Intervention based on the Theory of Planned Behaviour (TPB), Social Cognitive Theory (SCT) and MI to improve diet and physical activity	General health information for cancer survivors + waiting list	Exercise physiologist, nutritionist, survivor coach	-	-	3	Combination of individual and group	Combination of in-person and remote delivery	12	52.5
Simpson 2015 (intensive)	Intensive, individually tailored MI sessions + professional-led peer group support sessions	Information pack based on useful resources for weight loss and healthy lifestyle, and advice on weight loss	Motivational interviewing practitioners and group facilitators	Training in motivational interviewing	Quality of MI delivery; Are MIPs delivering the WLM and theory components; Barriers to delivering the intervention; Participant's understanding or experience of the intervention. Out of all practitioners and all measures, in only 6% of the ratings did MIPs fail to reach proficiency. For most measures practitioners reached proficiency or competence.	12	Individual	Combination of in-person and remote delivery	15	40
Simpson 2015 (less intensive)	Less intensive, individually tailored MI sessions + professional-led peer group support sessions	Information pack based on useful resources for weight loss and healthy lifestyle, and advice on weight loss	Motivational interviewing practitioners and group facilitators	Training in motivational interviewing	Quality of MI delivery; Are MIPs delivering the WLM and theory components; Barriers to delivering the intervention; Participant's	12	Individual	Combination of in-person and remote delivery	4	36

					understanding or experience of the intervention. Out of all practitioners and all measures, in only 6% of the ratings did MIPs fail to reach proficiency. For most measures practitioners reached proficiency or competence.					
Turner 2016	Telephone counselling including individually tailored feedback and support using MI to increase physical activity + telehealth monitoring	Home DVD including health information to promote physical activity	Study therapist	Multiday MI training program conducted by an experienced MI trainer unrelated to the study + training from the study principal investigator + experiential and role play exercises, case-consultation exercises utilizing core MI skills, pilot test on three test participants + weekly (tapered to biweekly over the course of the study) supervision with the principal investigator	A 10% random subsample of treatment sessions was selected and reviewed using a fidelity checklist of behaviours consistent with MI practices and spirit. Across sessions, 75.1% of questions were open-ended, the ratio of reflections to questions was. 98% and 98.3% of counsellor behaviour was MI-consistent. On average, participants exhibited .15 (SD .49) resistant behaviours per session. The average rating of MI global style was 4.8 (range 4–5). With the exception of the reflection-to-question ratio, counsellor behaviour met or exceeded established guidelines.	6	Individual	Telephone	6	52.5
Tuvemo 2020	Home-based exercise program including balance training,	Pamphlet with general safety recommendations for	Physical trainers	3-day MI education session and three booster sessions by	The average was 3.8 for the PTs, which was interpreted as an	12	Individual	Combination of in-person and	9	60

	strength training, and walks, with collaborative conversations according to the MI to reinforce and activate the participants' intrinsic resources	older adults, including fall prevention recommendations		two MINT (Motivational Interviewing Network of Trainers) instructors	acceptable score rated on a 5-point Likert scale, where 0 is a low MI spirit and 5 is high			remote delivery		
Valeiro 2022	A motivational interview to explore the patient's ambivalences and to assist them in tipping the decision balance in favour of change + a personalized physical activity program with a pedometer and a printed calendar + weekly telephone calls to assess their progress, discuss possible barriers and set new weekly physical activity goals	Standard clinical discharge management plan: brief advice to meet international recommendations and to complete their prescribed medical treatment	Respiratory physiotherapist	Adequate training in behavioural strategies	-	3	Individual	In person	1	-
Van Keulen 2011 (MI and others)	Telephone calls based on motivational interviewing discussing PA and fruit and vegetable consumption	Tailored letter addressing PA and fruit and vegetable consumption after study	Motivational interviewing counsellors	Six 3-h training sessions by two certified trainers	-	12	Individual	Telephone	2	-
Van Keulen 2011 (MI only)	Telephone calls based on motivational interviewing discussing PA and fruit and vegetable consumption + Tailored Print Communication	Tailored letter addressing PA and fruit and vegetable consumption after study	Motivational interviewing counsellors	Six 3-h training sessions by two certified trainers	-	12	Individual	Telephone	4	-

	including introduction, specific behavioural feedback on targeted behaviour, stage-matched advice to change behaviour, and conclusions									
vanBakel 2022	Face-to-face consultations for SIT LESS coaching + SIT LESS manual covering all core components of SIT LESS + activity tracker-derived ST reports + telephone coaching + usual care	Usual care, consisting of a comprehensive CR programme. One to three regular, individual consultations focusing on lifestyle, medication and psychosocial wellbeing + an outpatient physical activity programme, consisting of~12 supervised, one hour exercise group sessions across six weeks	Nurse specialists	Basic MI training as part of register nurse education + a comprehensive and accredited training course under the guidance of a behavioural psychologist. The training course consisted of a self-study document (±3 h of preparation) and an on-site group training (±8 h) where theory on MI techniques was explained and subsequently practiced through active role play	-	3	Individual	Combination of in-person and remote delivery	12	-
Vlaar 2017	Individual counselling using motivational interviewing + family session + cooking classes + supervised physical activity programme	Group sessions including generic information about T2D and discussion of current guidelines for diet and physical activity + leaflets with simple generic lifestyle advice	Dietitians	Training in motivational interviewing, in which previous successes, skills and strengths of the client were highlighted to support self-efficacy	-	6	Individual	In person	12	-
Weinstock 2019	Motivational interviewing to	Gym membership	Therapists (clinical psychologist,	An initial 2-day workshop on MI	Six independent raters assessed 42 randomly	4	Individual	In person	2	50

	increase exercise + contingency management (CM) exercise contracting sessions		exercise specialists, advanced clinical psychology doctoral students)	lead by an outside expert and a 1-day workshop on CM followed by annual 1-day refresher trainings. Ongoing supervision consisted of regular review of intervention binders, audiotapes, and case discussion	selected audiotapes (about 10% of all sessions) of the MI intervention. For the sessions that included MI, mean rating and standard deviation of the MI-related items were 5.11 (0.90; reflecting average rankings of "good/quite a bit"). The MI component of the intervention was distinguishable and rated as having "good" therapist adherence and competence.					
West 2016	Behavioural weight control treatment as control group + MI chat sessions	Chat sessions + website with behavioural lessons, educational resources, regularly updated tips and notices for weight loss and physical activity, self-monitoring tool + email with tailored feedback + pedometers + weekly homework	Clinical psychologists	Training and ongoing supervision in MI from the first author, a MI network trainer and a clinical psychologist	MI chat transcripts were reviewed and constructive feedback provided to refine therapist skills. Group telephonic coaching was provided weekly with a focus on maintaining an MI spirit, adhering to the protocol, and role-playing around difficulties encountered during MI chats.	18	Individual	Web-based	6	30
Whitehead 2022	Mailed intervention materials + two phone delivered brief interventions adapted from MI to improve physical activity + a one-page monthly physical activity newsletter	Mailed information on reducing the sodium in their diet + telephone calls regarding a different topic, e.g. low sodium diet.	Masters level therapist	Two trainings in MI techniques	Fidelity ensured by self-rating from interventionist and discussion with a clinical supervisor	6	Individual	Telephone	2	17.5

Young 2019	MI calls + packet including information about rate of perceived exertion scale, expected changes while exercising, tips about exercising, 24-week physical activity log, and a KP-branded physical activity personal action plan	Physical activity resource handout that including fitness classes and facilities, their costs, and available walking paths located in their communities as well as online resources	Research associate	A 30-h web-based MI program with webinars, skill-building activities and a 2-day face-to-face training with a MI expert trainer	-	6	Individual	Telephone	7	20

Supplementary Figure 1: Risk of bias assessment for individual studies

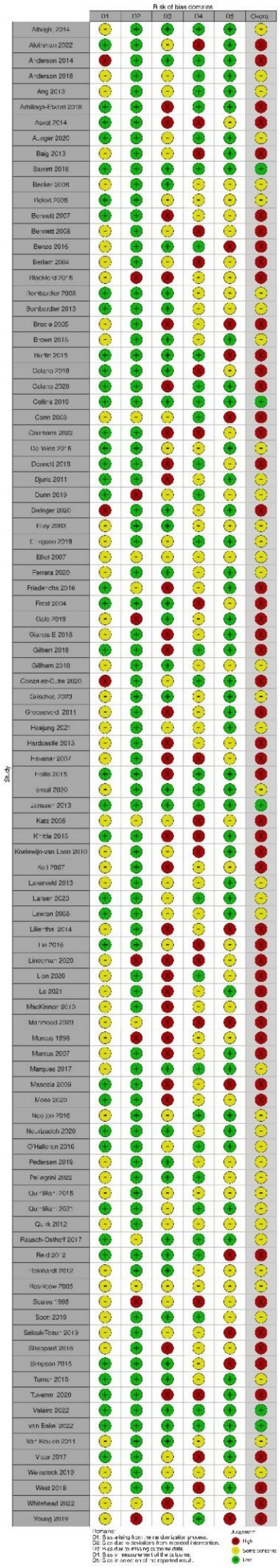

Supplementary Figure 2: Funnel plots with pseudo 95% confidence intervals of studies reporting total PA (A) MVPA (B) and sedentary time (C) outcomes.

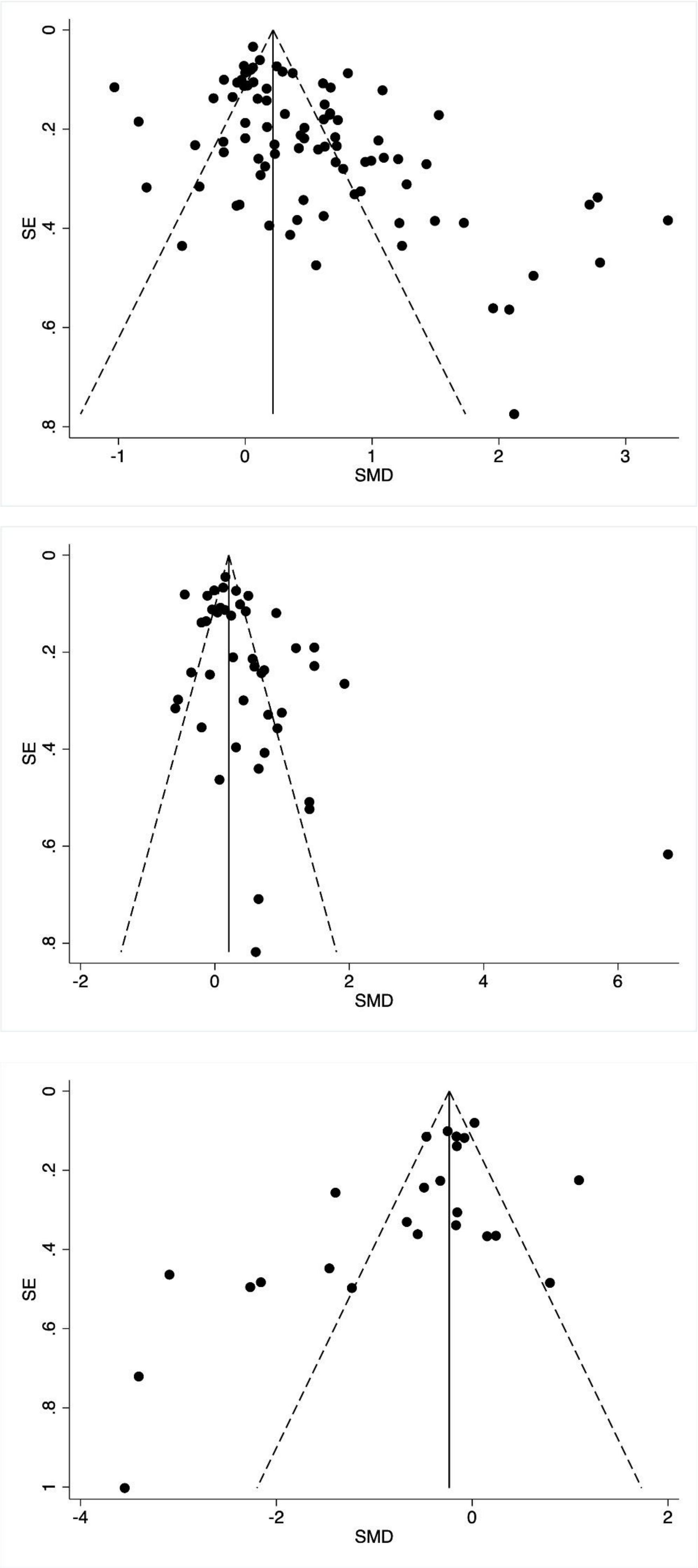

Supplementary Figure 3: Forest plot on studies reporting total physical activity.

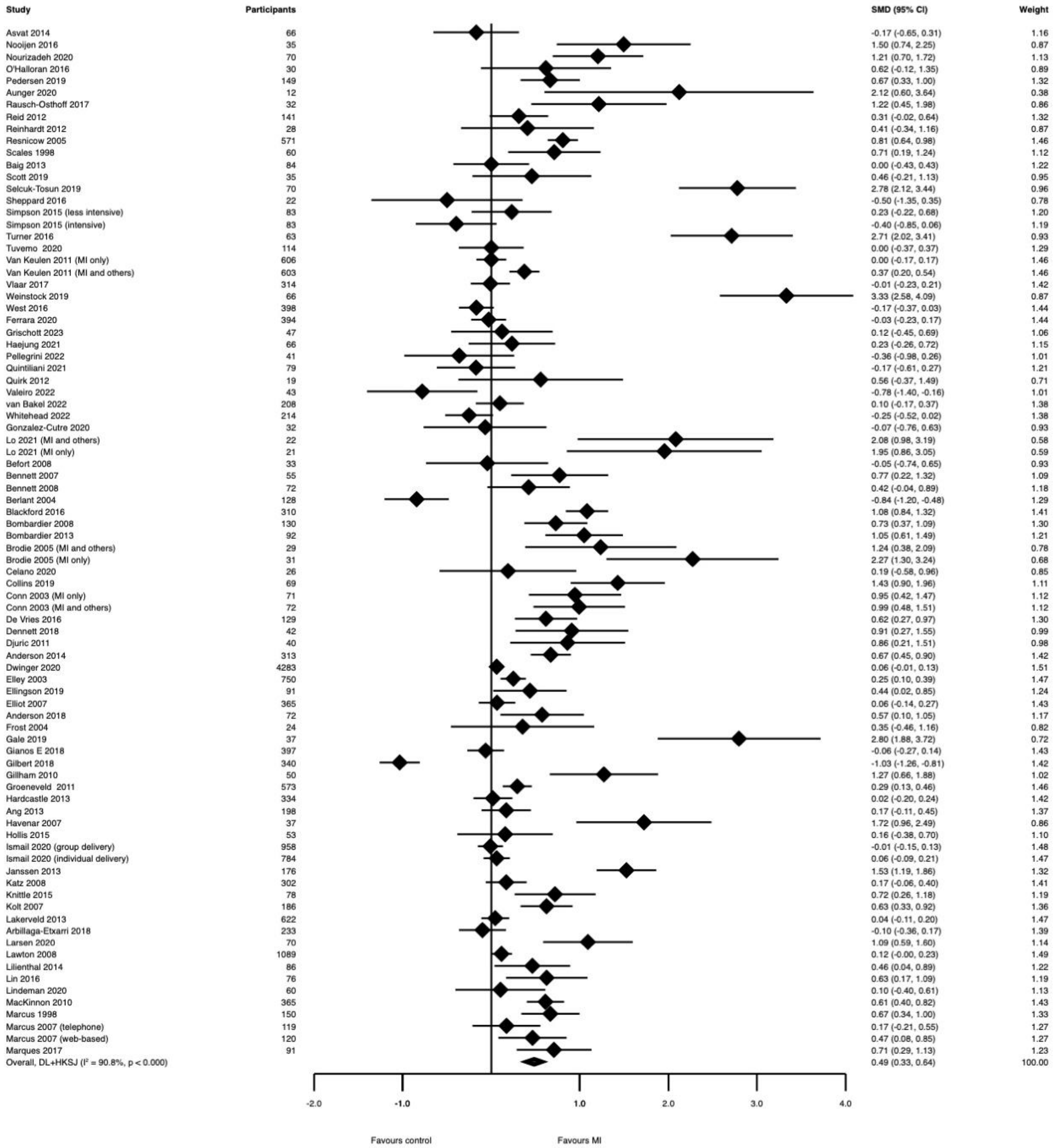

Supplementary Figure 4: Forest plot on studies reporting moderate & vigorous physical activity (longest follow-up).

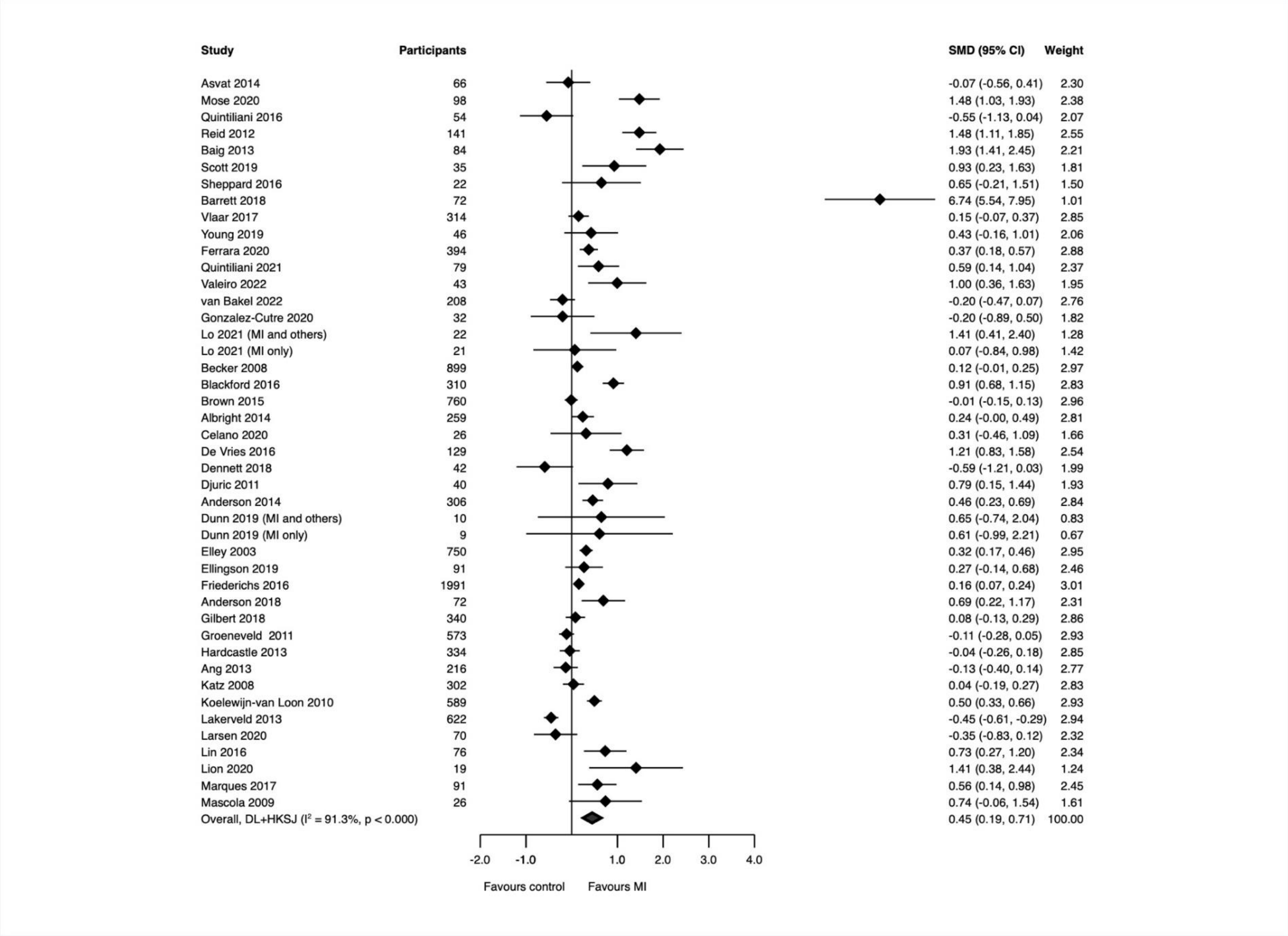

Supplementary Figure 5: Forest plot on studies reporting sedentary time (longest follow-up).

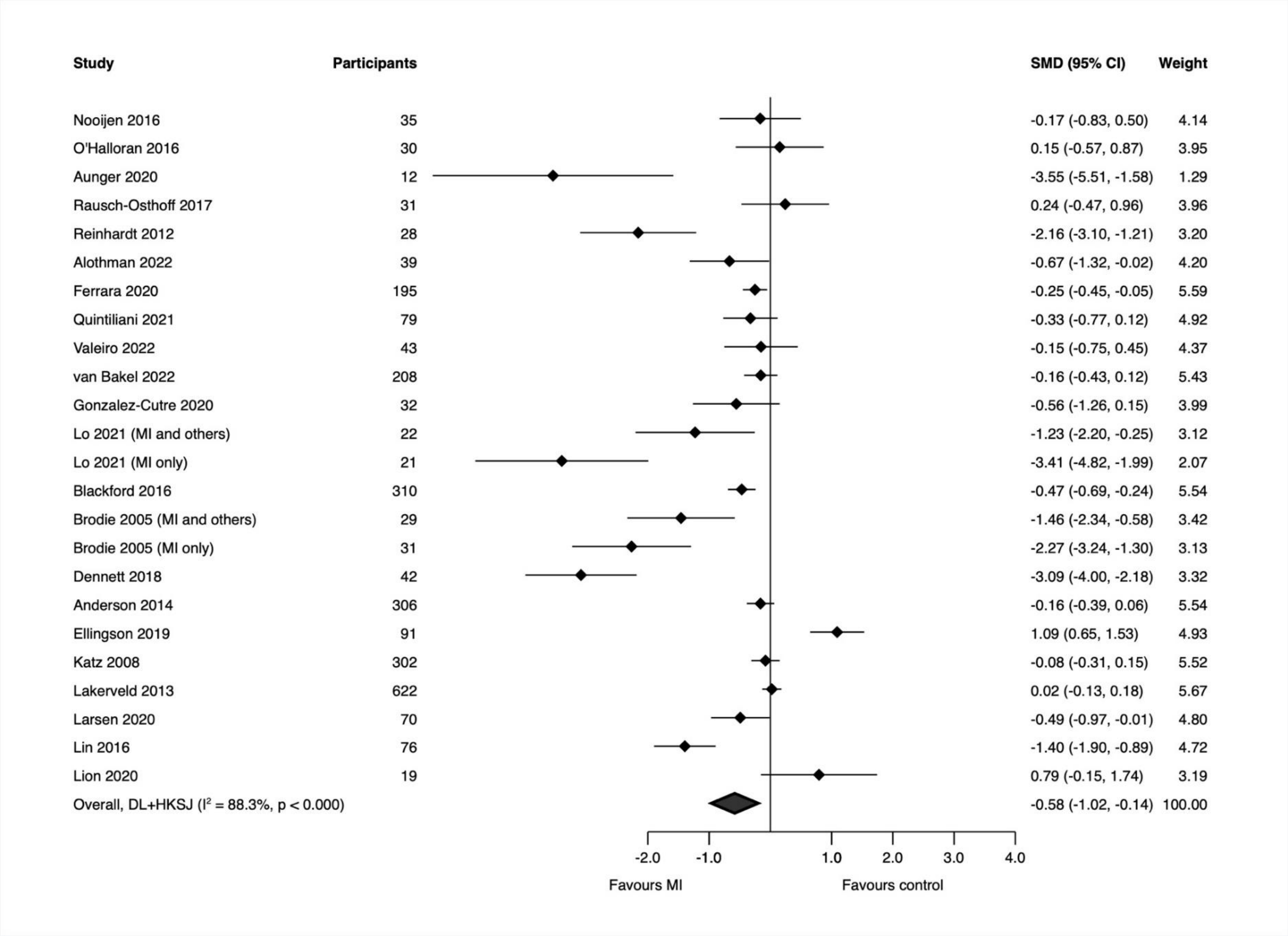

Supplementary Figure 6: Forest plot of studies comparing MI interventions with no or minimal intervention comparators on Total PA.

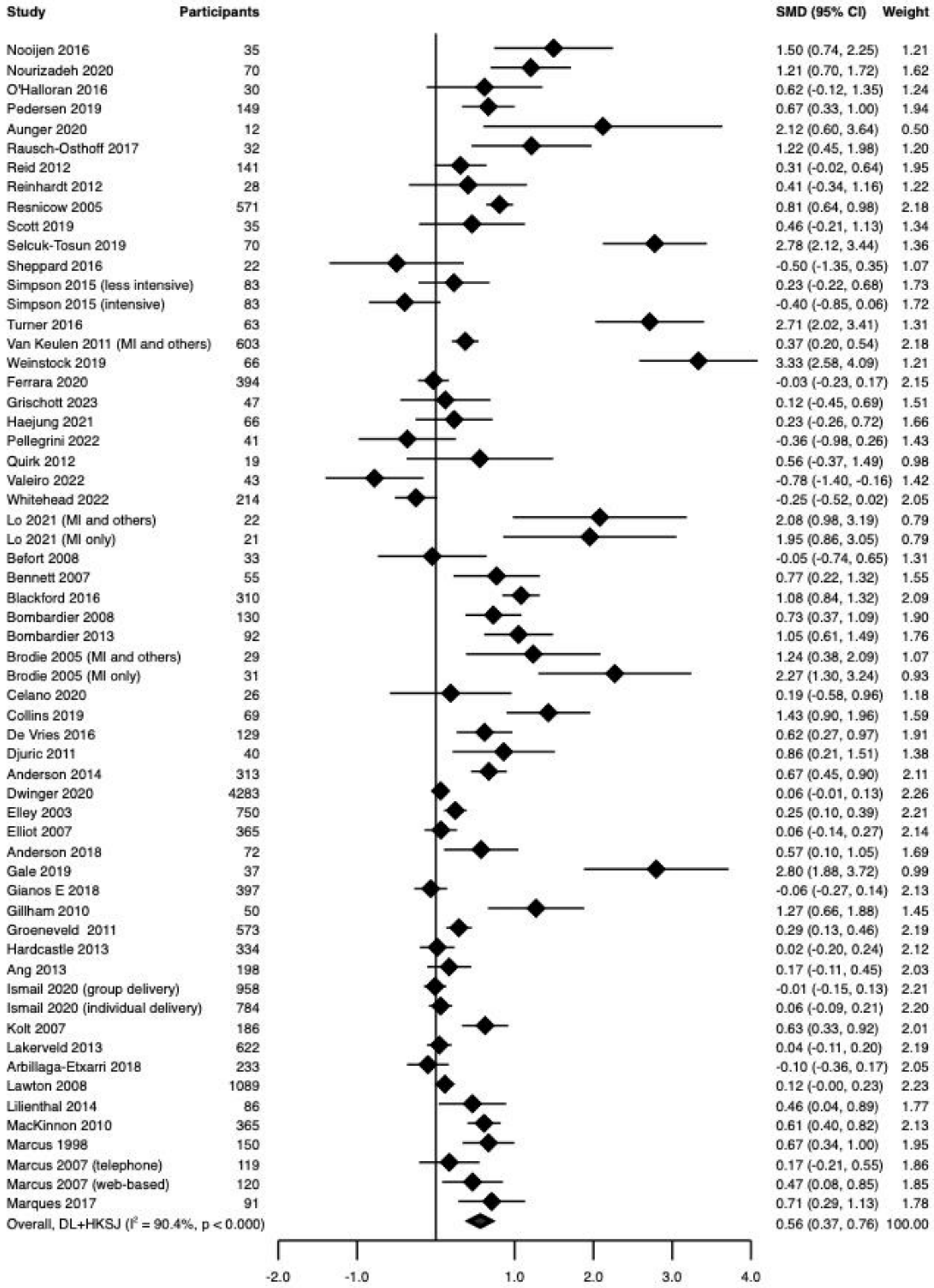

Supplementary Figure 7: Forest plot of studies comparing MI interventions with no or minimal intervention comparators on MVPA

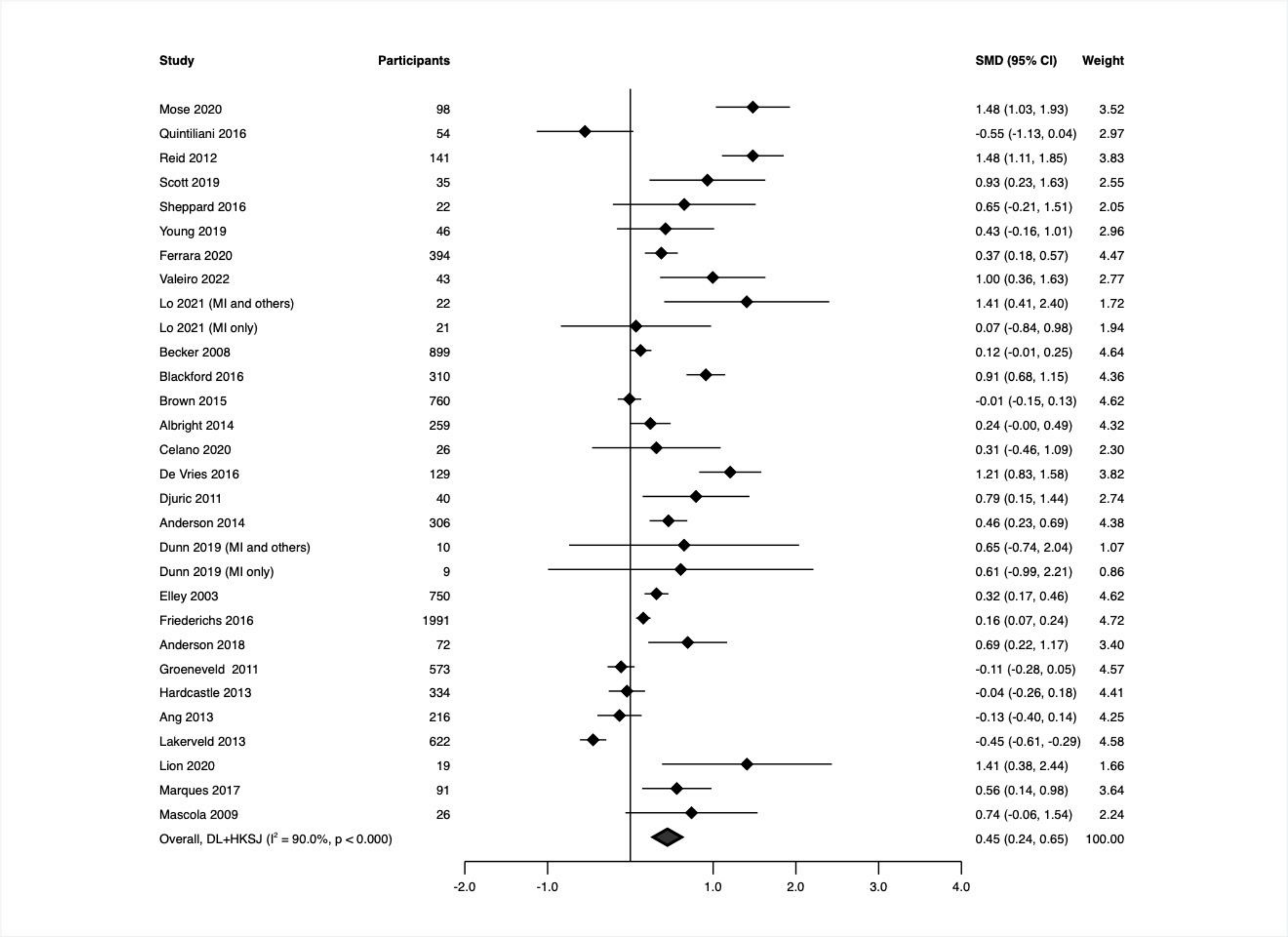

Supplementary Figure 8: Forest plot of studies comparing MI interventions with no or minimal intervention comparators on sedentary time.

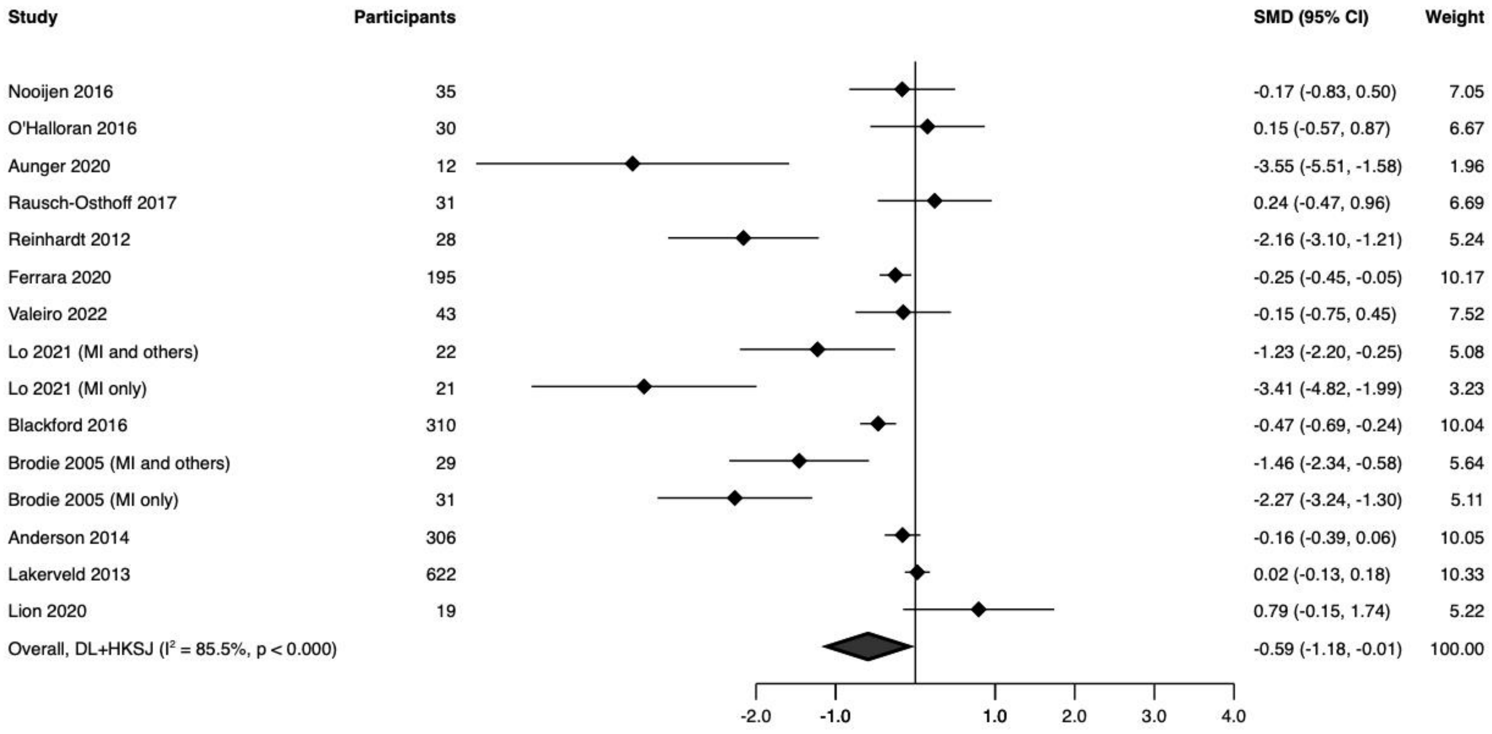

Supplementary Figure 9: Forest plot of studies comparing MI interventions with other active interventions of lower intensity that did not include MI on Total PA, MVPA and sedentary time.

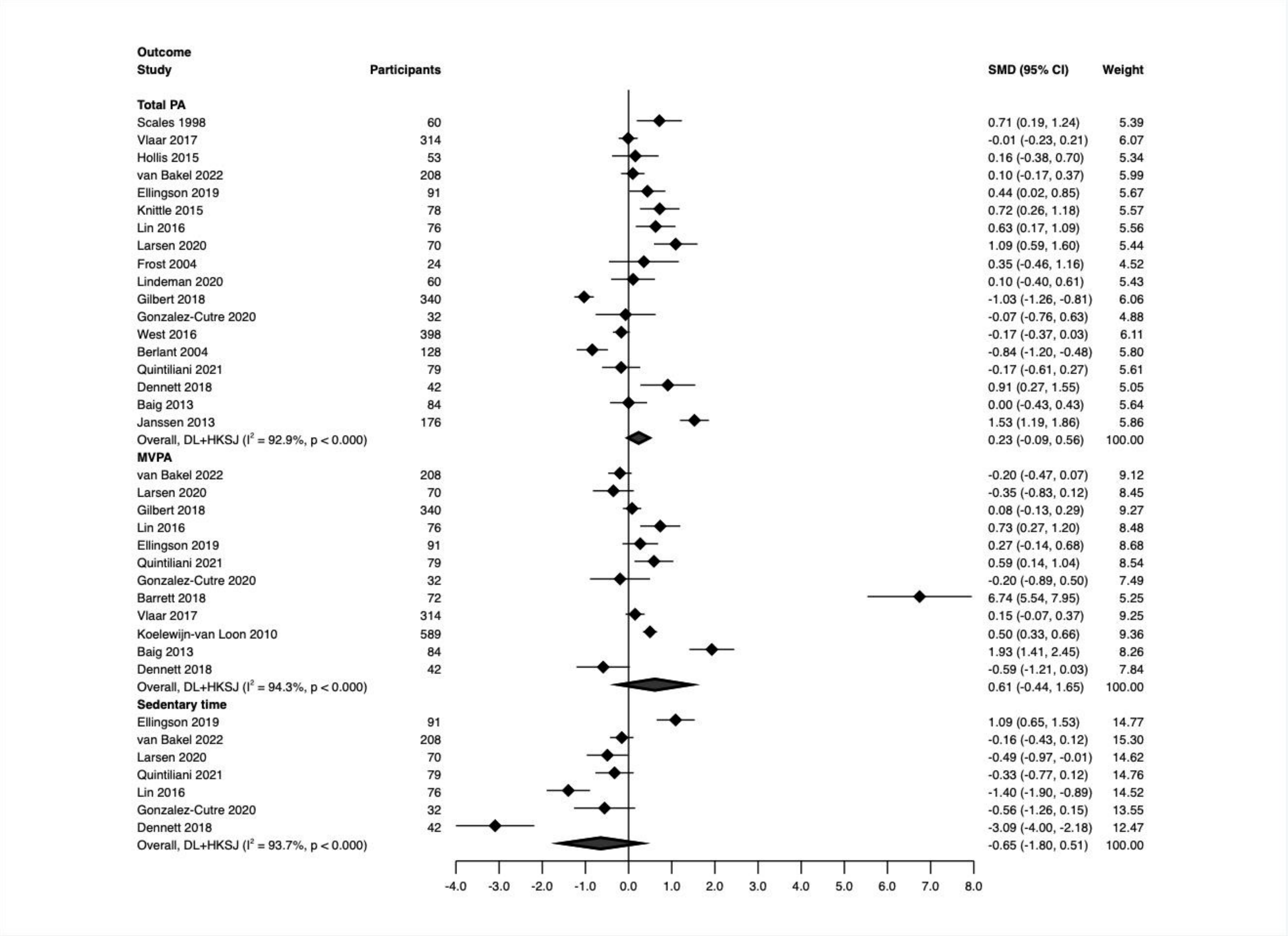

Supplementary Figure 10: Forest plot of studies comparing MI interventions vs. comparator interventions of similar or lesser intensity on all outcomes.

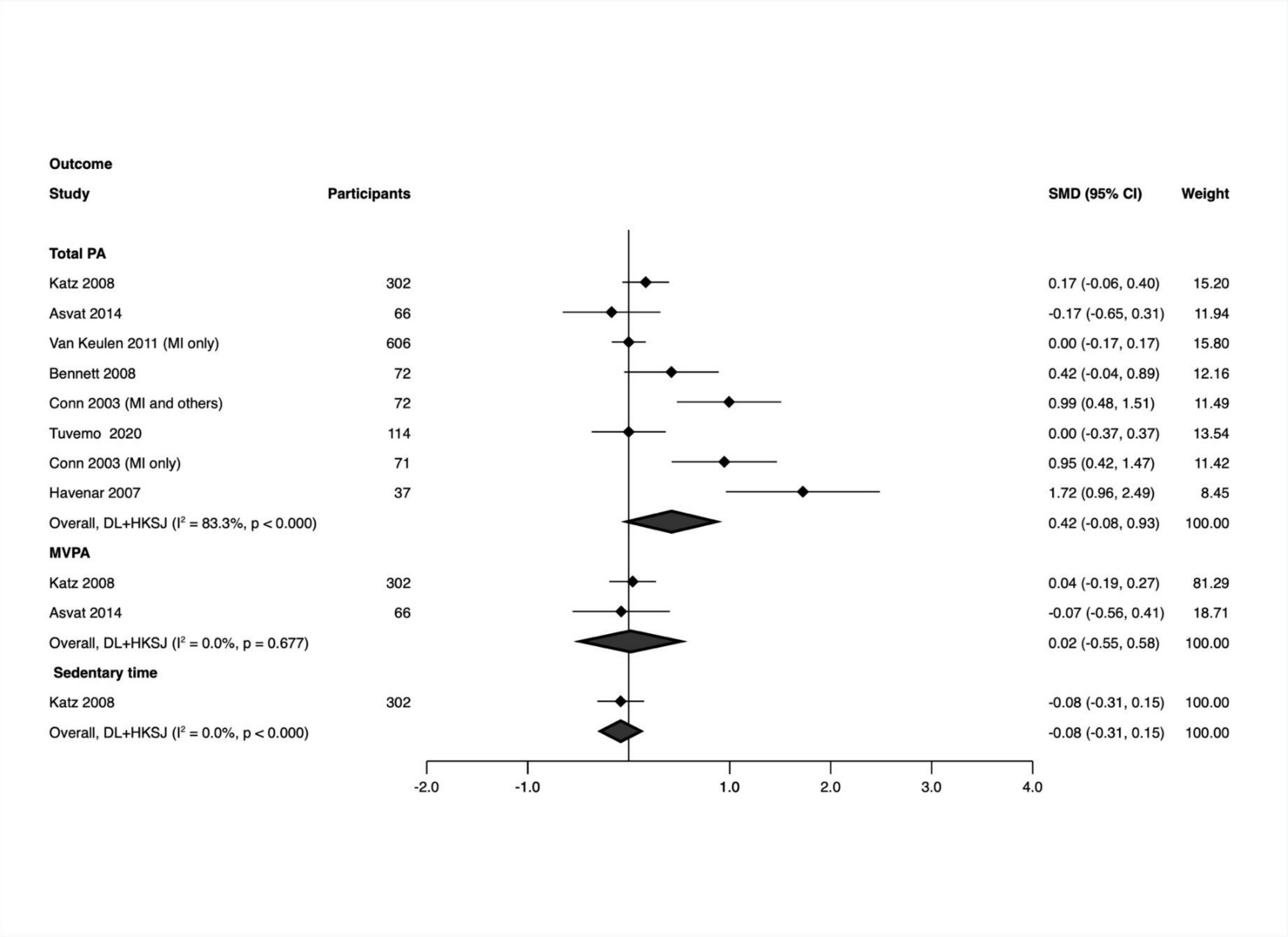

Supplementary Figure 11: Forest plot of studies using device measured outcomes on total PA, MVPA and sedentary time outcomes

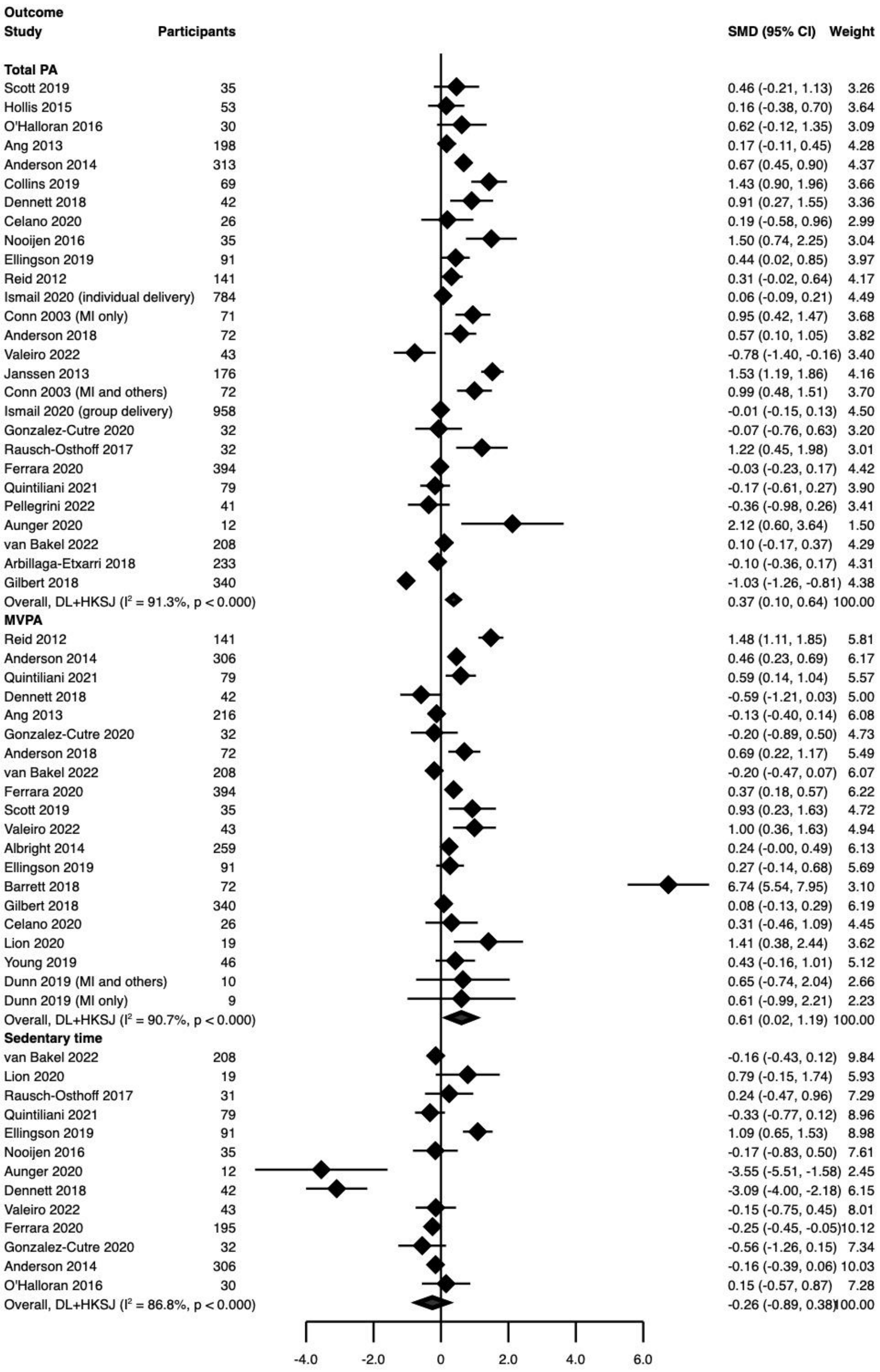

Supplementary Figure 12: Forest plot of studies using self-reported outcome assessment methods for total PA outcomes

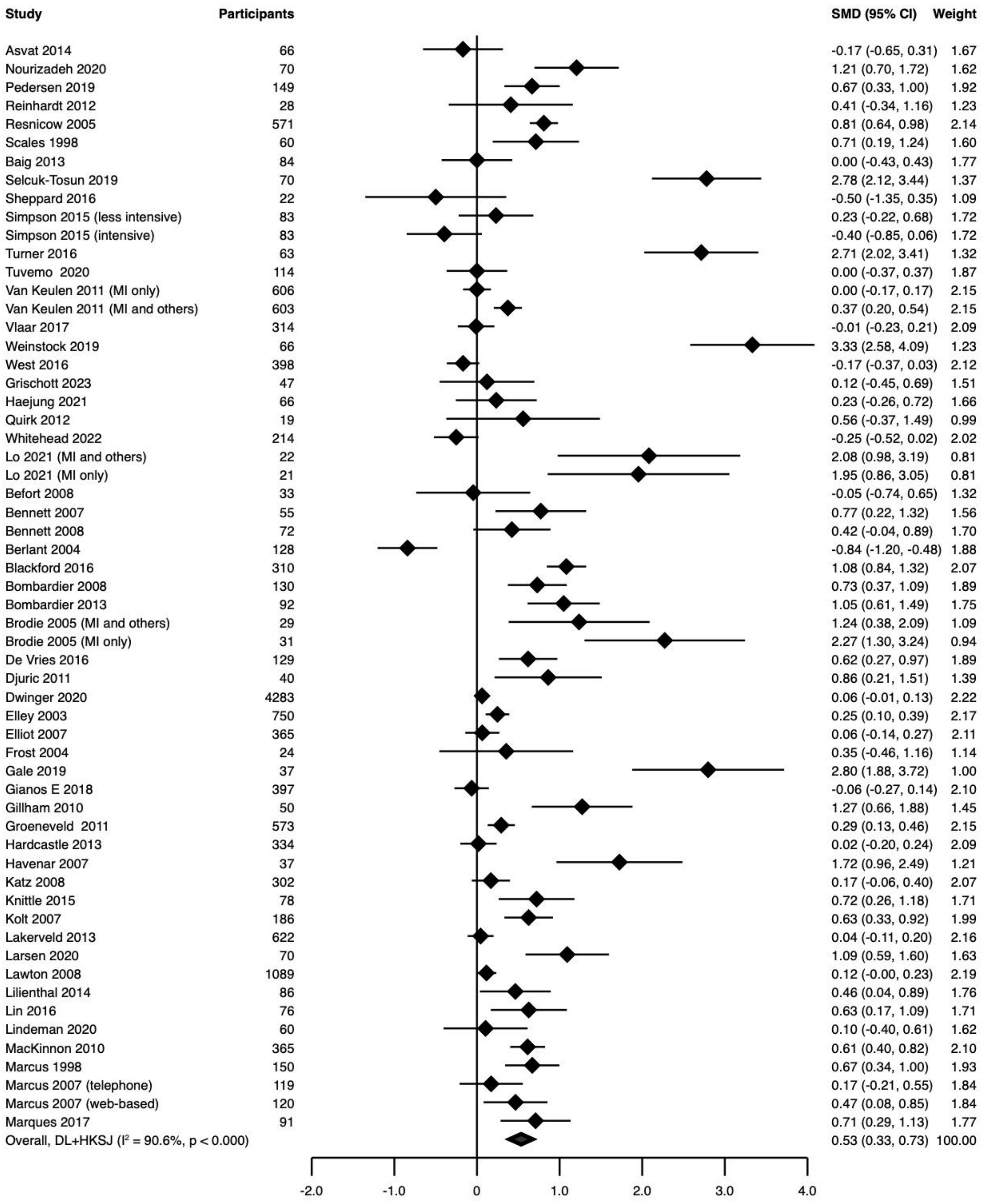

Supplementary Figure 13: Forest plot of studies using self-reported outcome assessment methods for MVPA and sedentary time outcomes

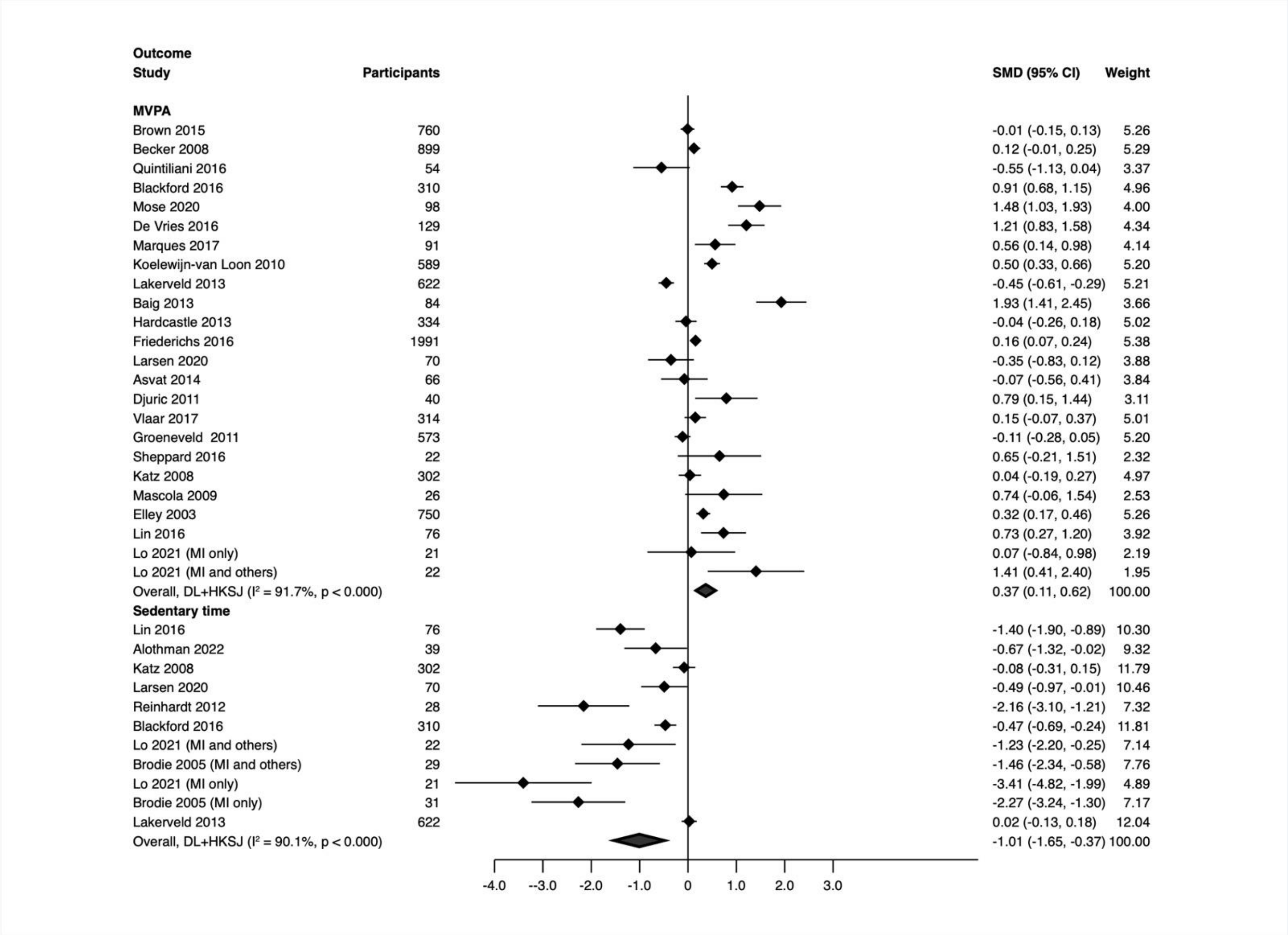

Supplementary Figure 14: Forest plot of studies reporting total PA at 0-3 months follow-up

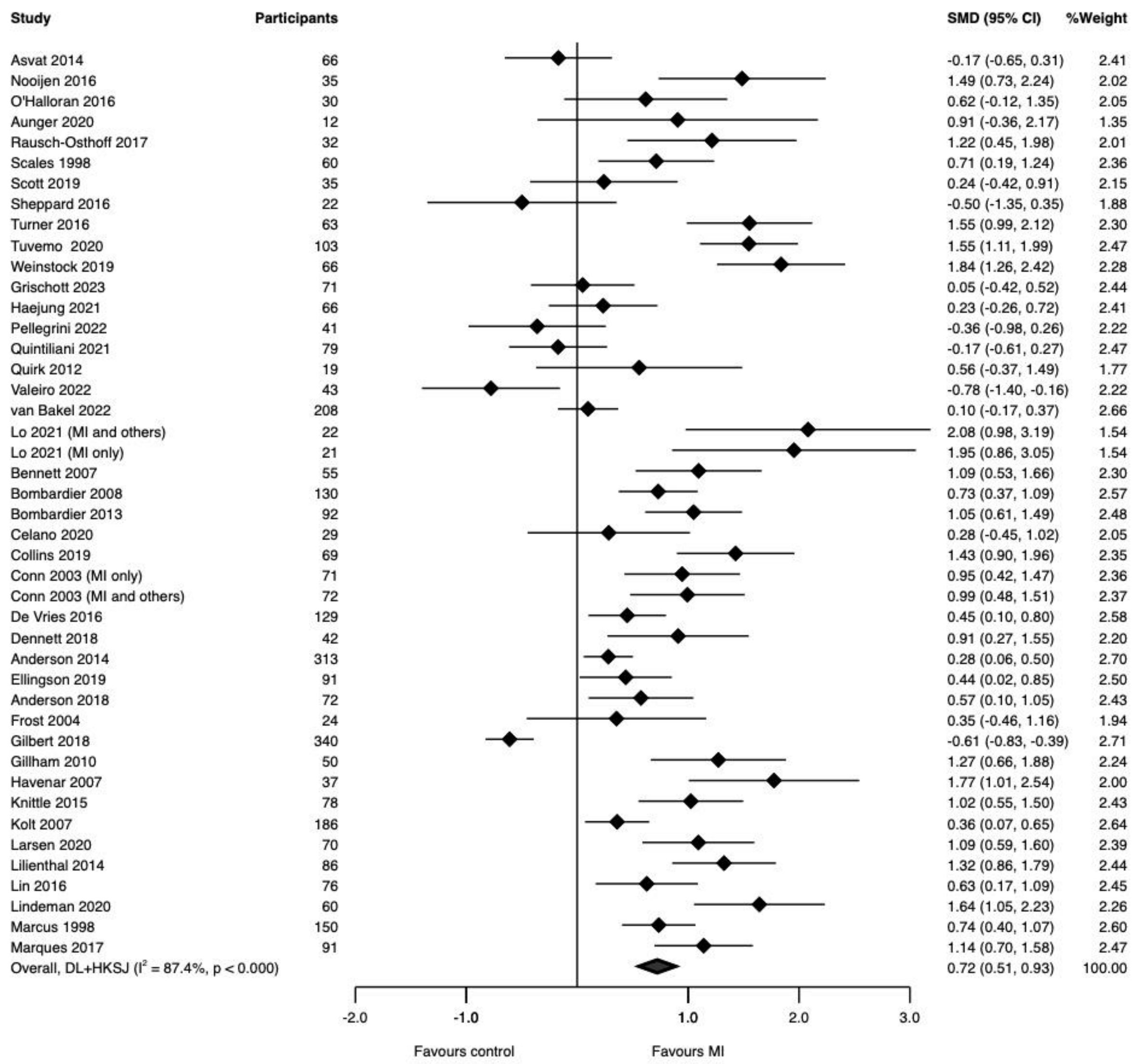

Supplementary Figure 15: Forest plot of studies reporting total PA at 4-6 months follow-up

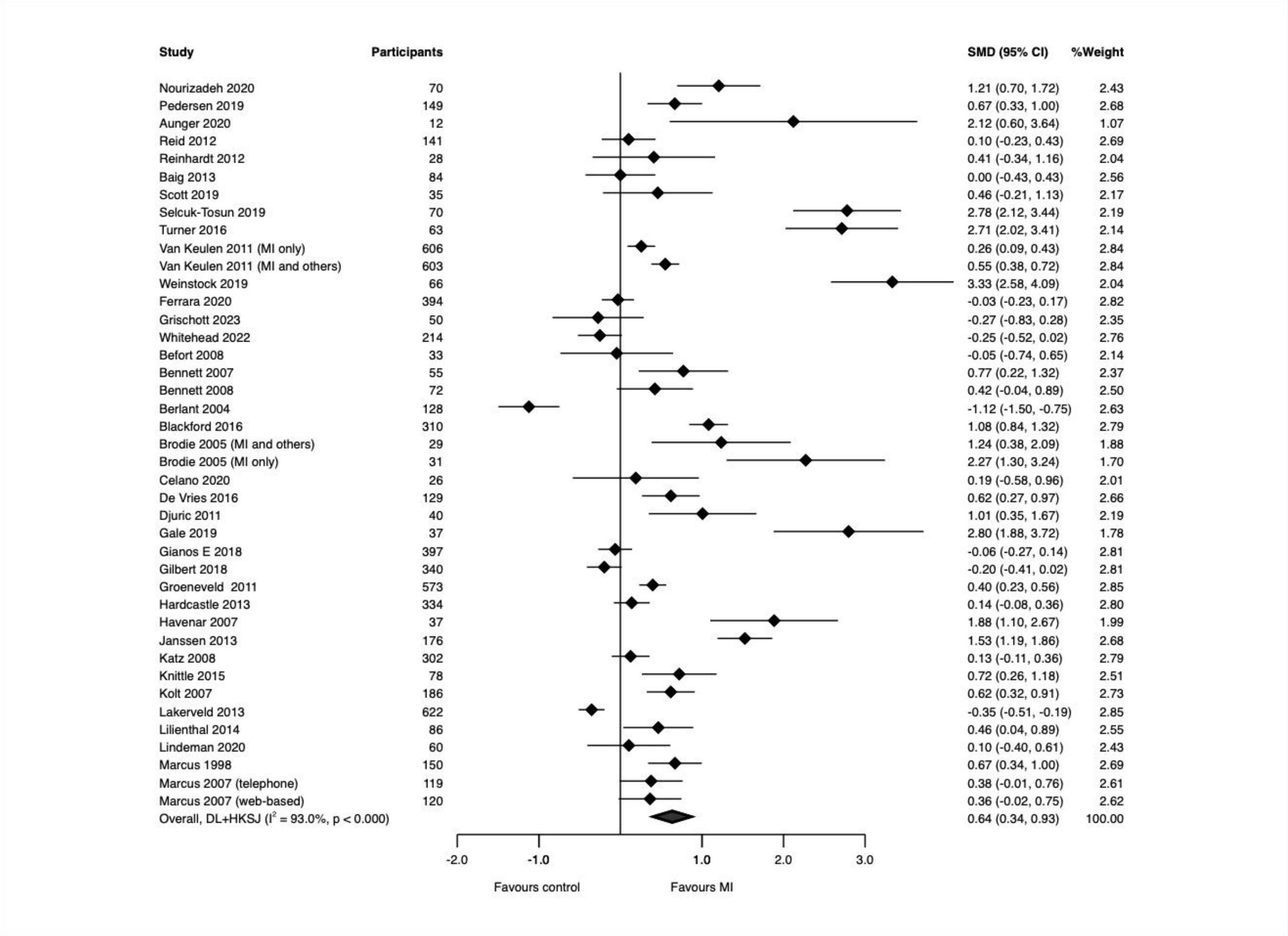

Supplementary Figure 16: Forest plot of studies reporting total PA at 7-12 months follow-up

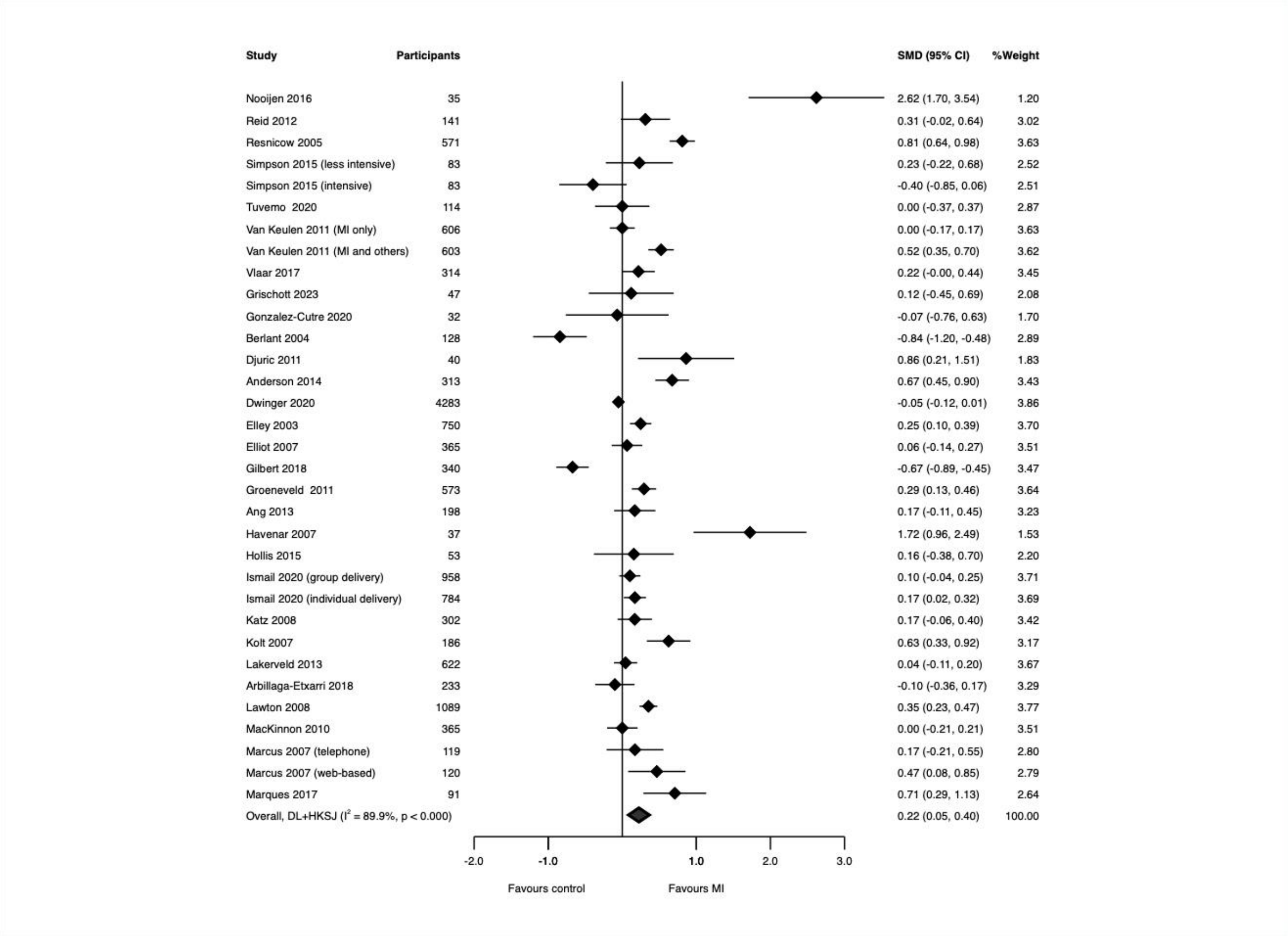

Supplementary Figure 17: Forest plot of studies reporting total PA of >1 year follow-up

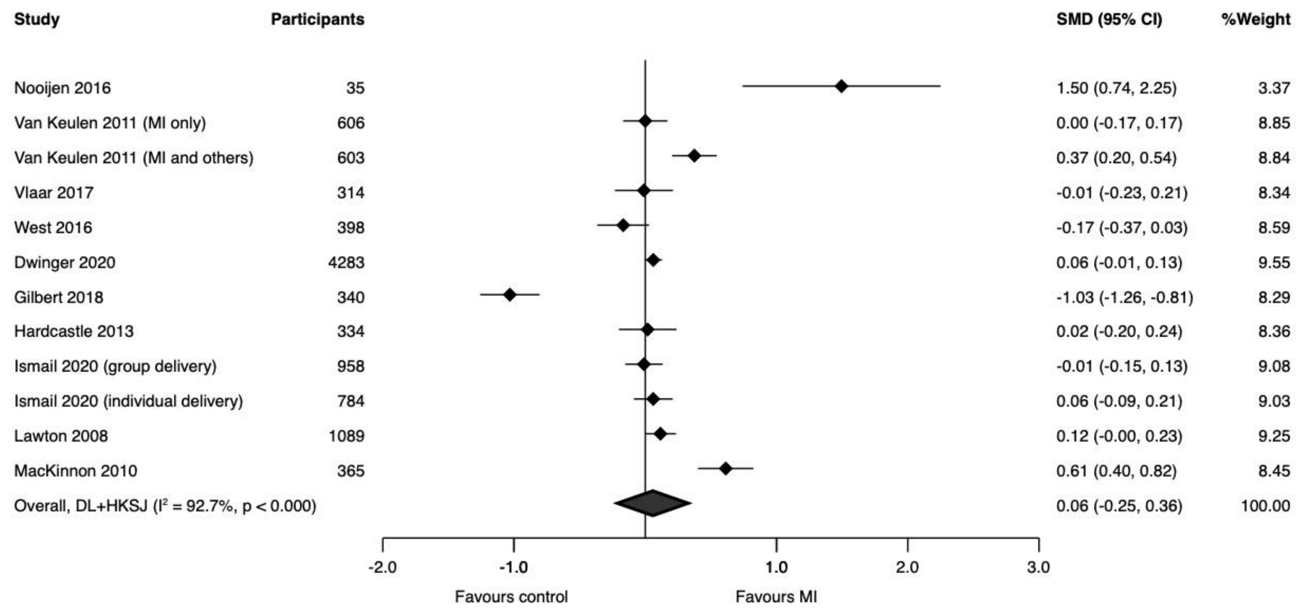

Supplementary Figure 18: Forest plot of studies reporting total MVPA at 0-3 months follow-up

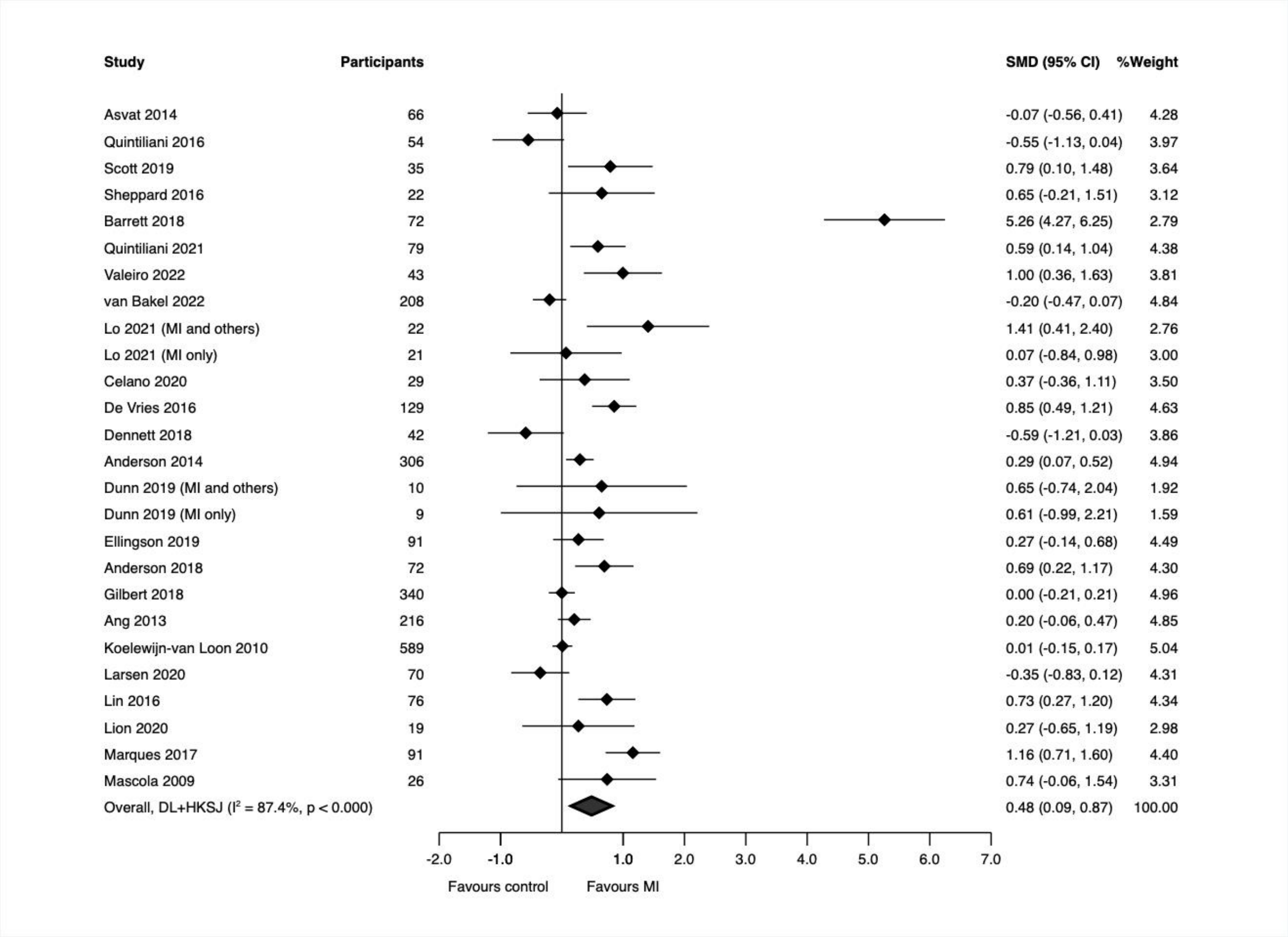

Supplementary Figure 19: Forest plot of studies reporting total MVPA at 4-6 months follow-up

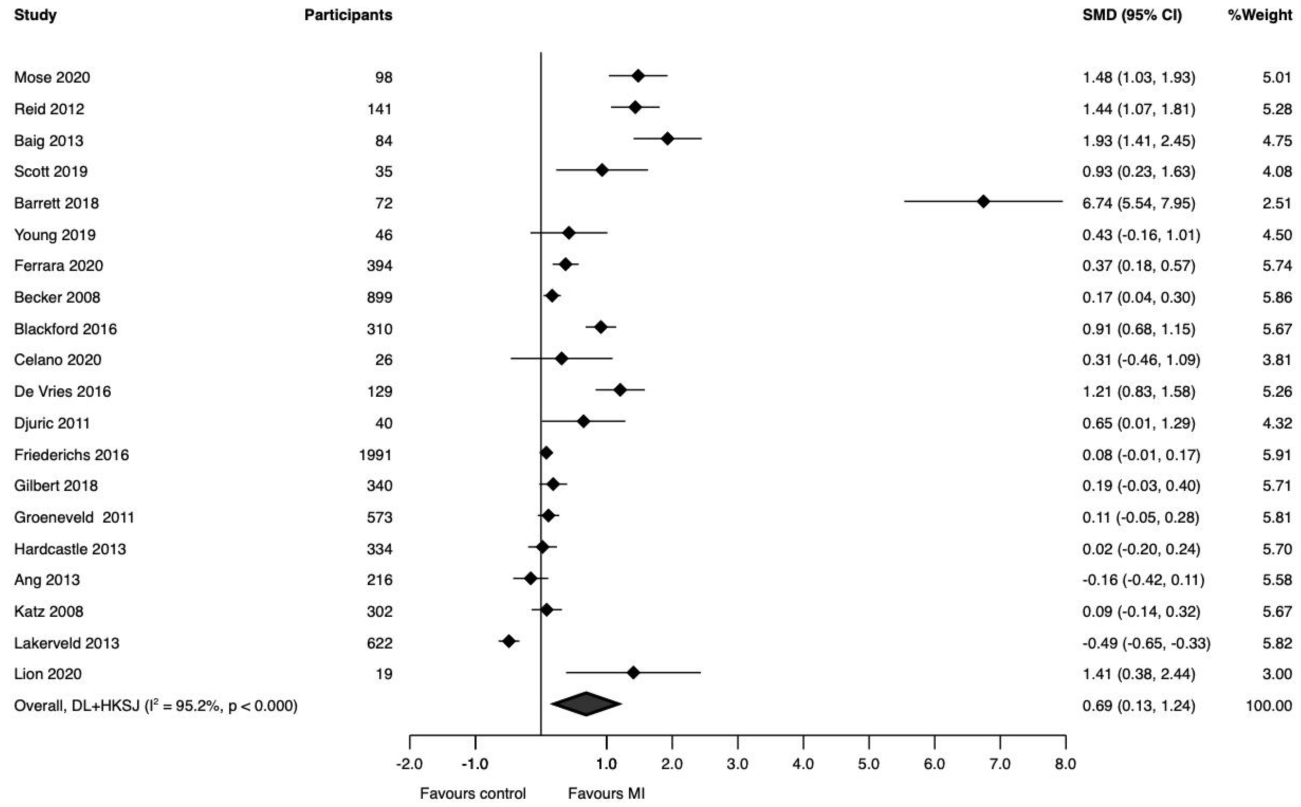

Supplementary Figure 20: Forest plot of studies reporting total MVPA at 7-12 months follow-up

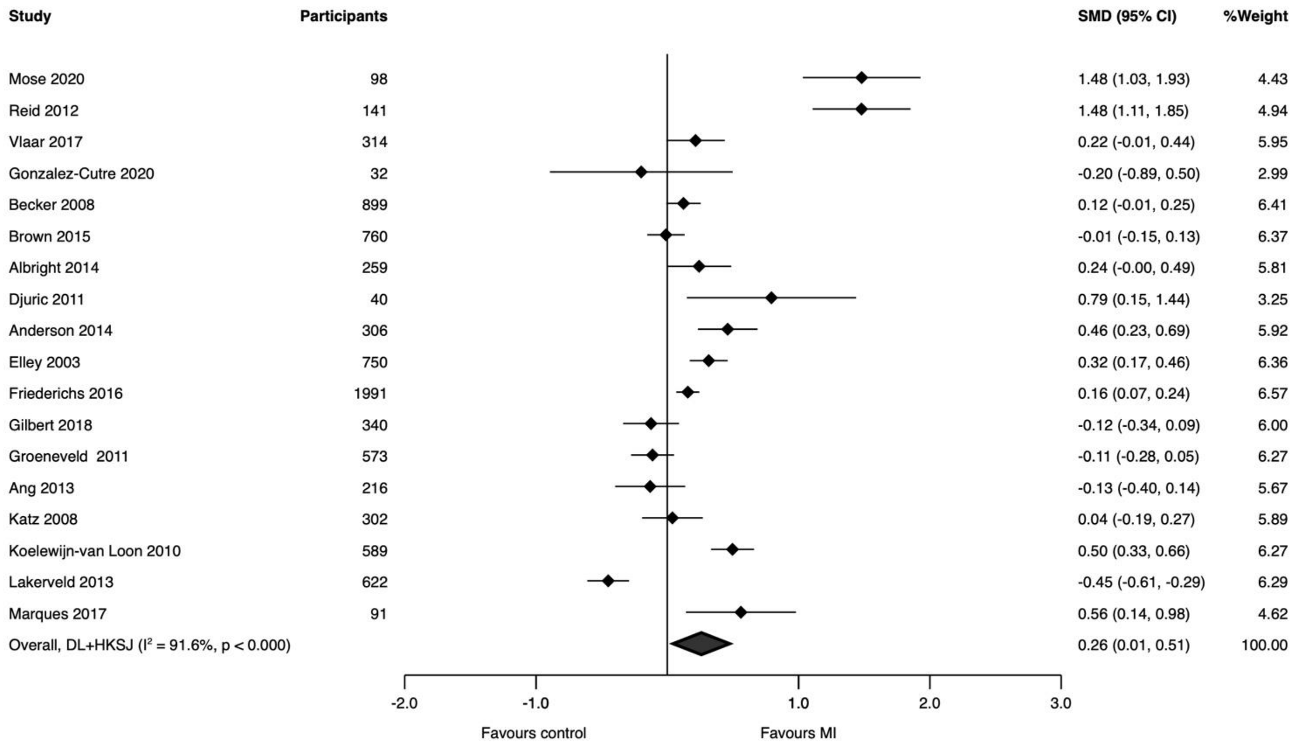

Supplementary Figure 21: Forest plot of studies reporting total MVPA at >1 year follow-up

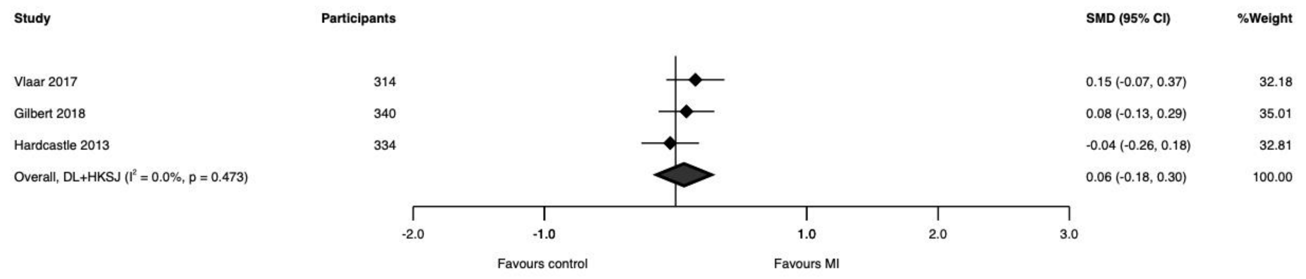

Supplementary Figure 22: Forest plot of studies reporting sedentary time at 0-3 months follow-up

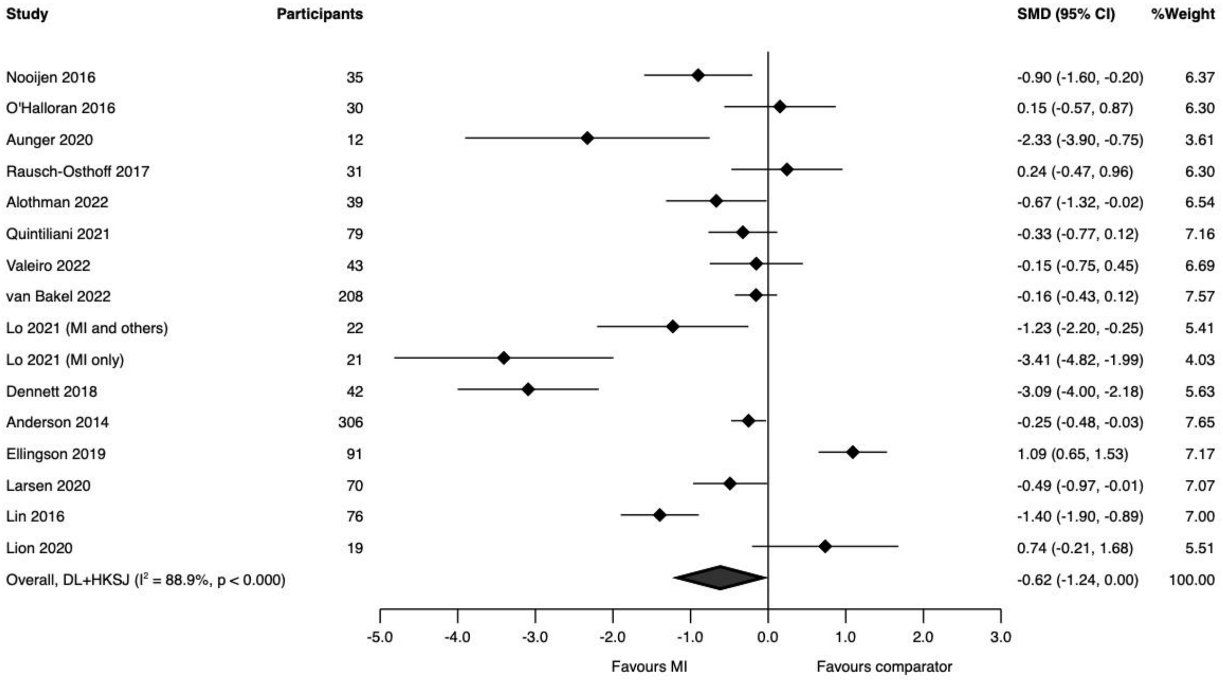

Supplementary Figure 23: Forest plot of studies reporting sedentary time at 4-6 months follow-up

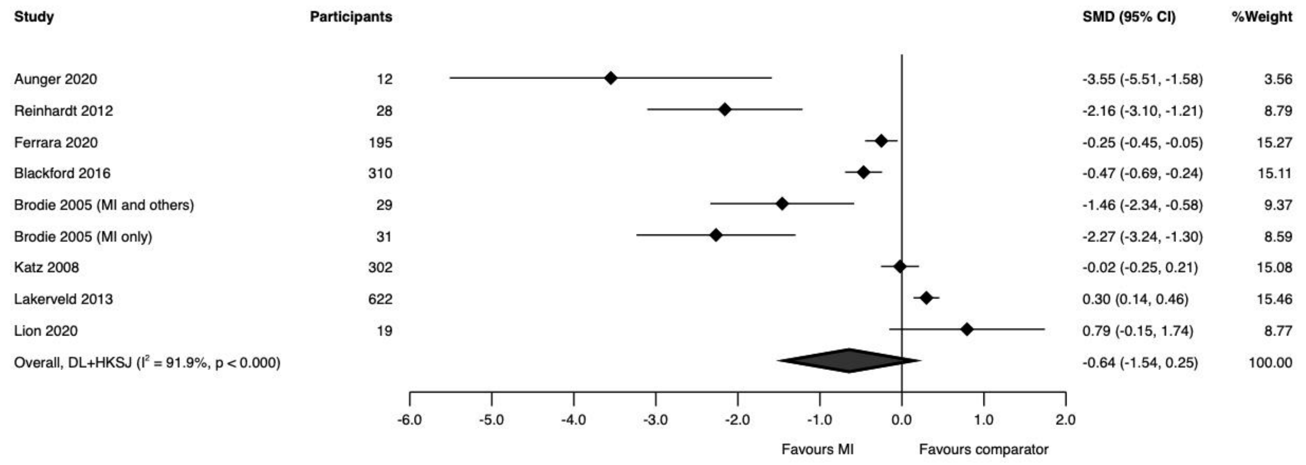

Supplementary Figure 24: Forest plot of studies reporting sedentary time at 7-12 months follow-up

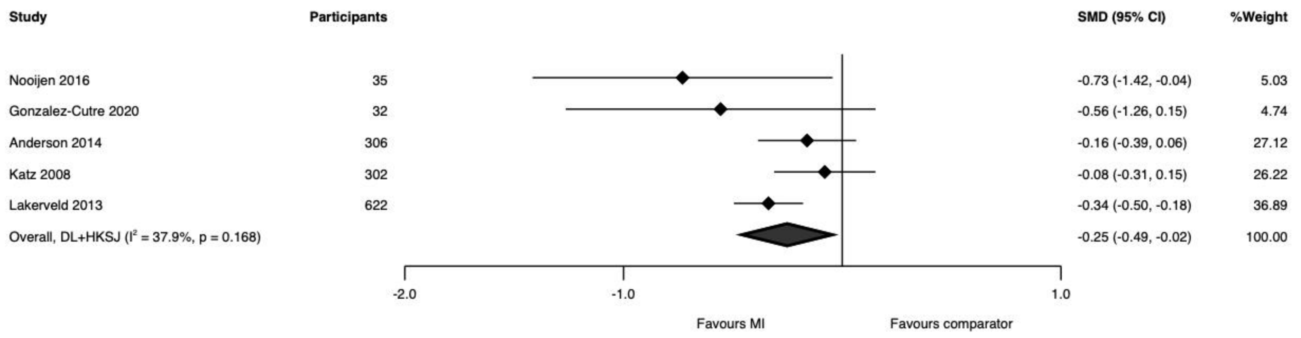

Supplementary Figure 25: Forest plot of studies reporting sedentary time >1 year follow-up

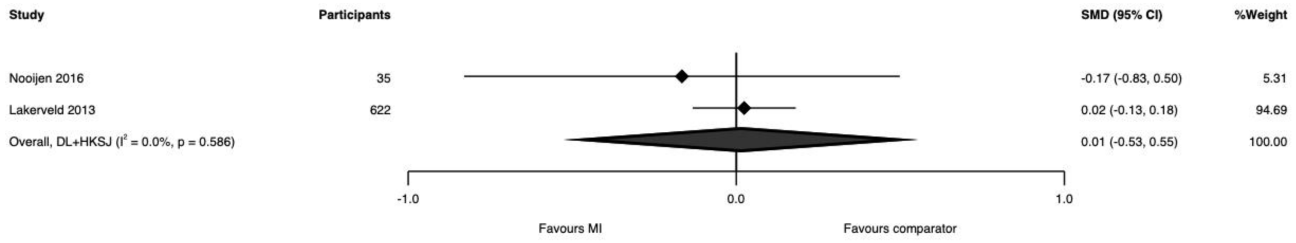

Supplementary Figure 26: Forest plot of studies with interventions 0-3 months duration reporting total physical activity outcomes at 0-3 months follow-up

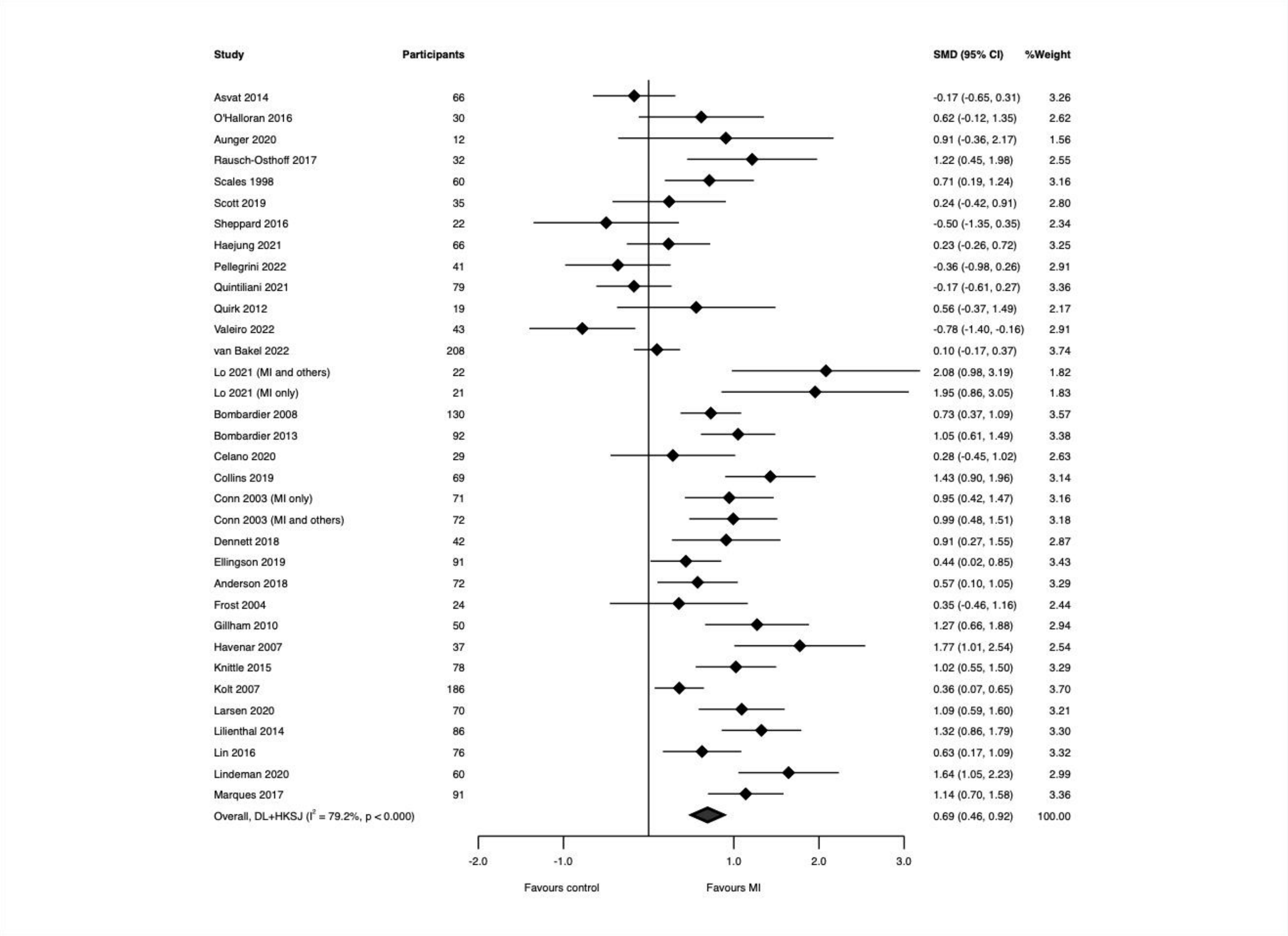

Supplementary Figure 27: Forest plot of studies with interventions 0-3 months duration reporting total physical activity outcomes at 4-6 months follow-up

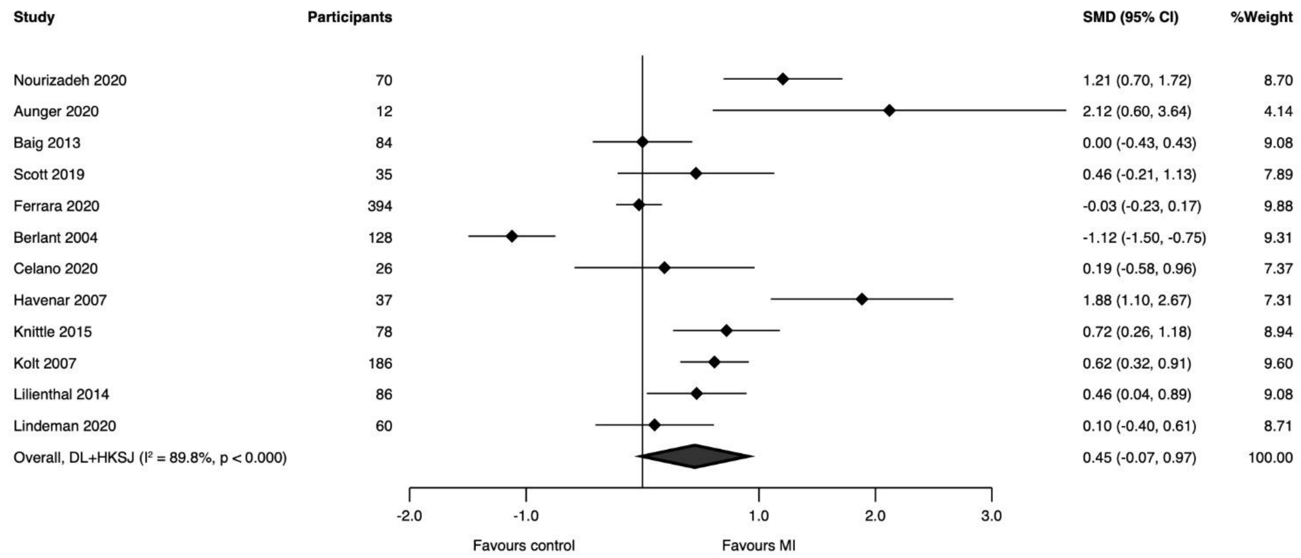

Supplementary Figure 28: Forest plot of studies with interventions 0-3 months duration reporting total physical activity outcomes at 7-12 months follow-up

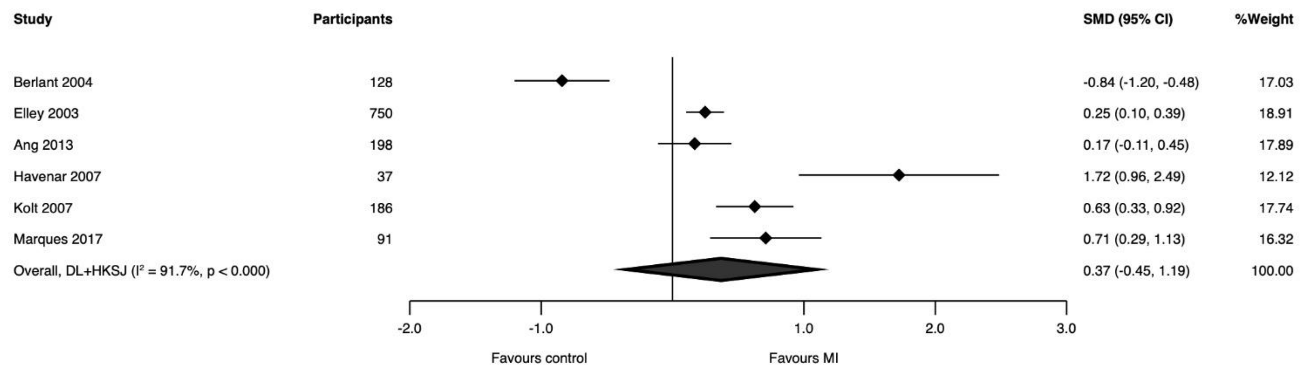

Supplementary Figure 29: Forest plot of studies with interventions 4-6 months duration reporting total physical activity outcomes at 0-3 months follow-up

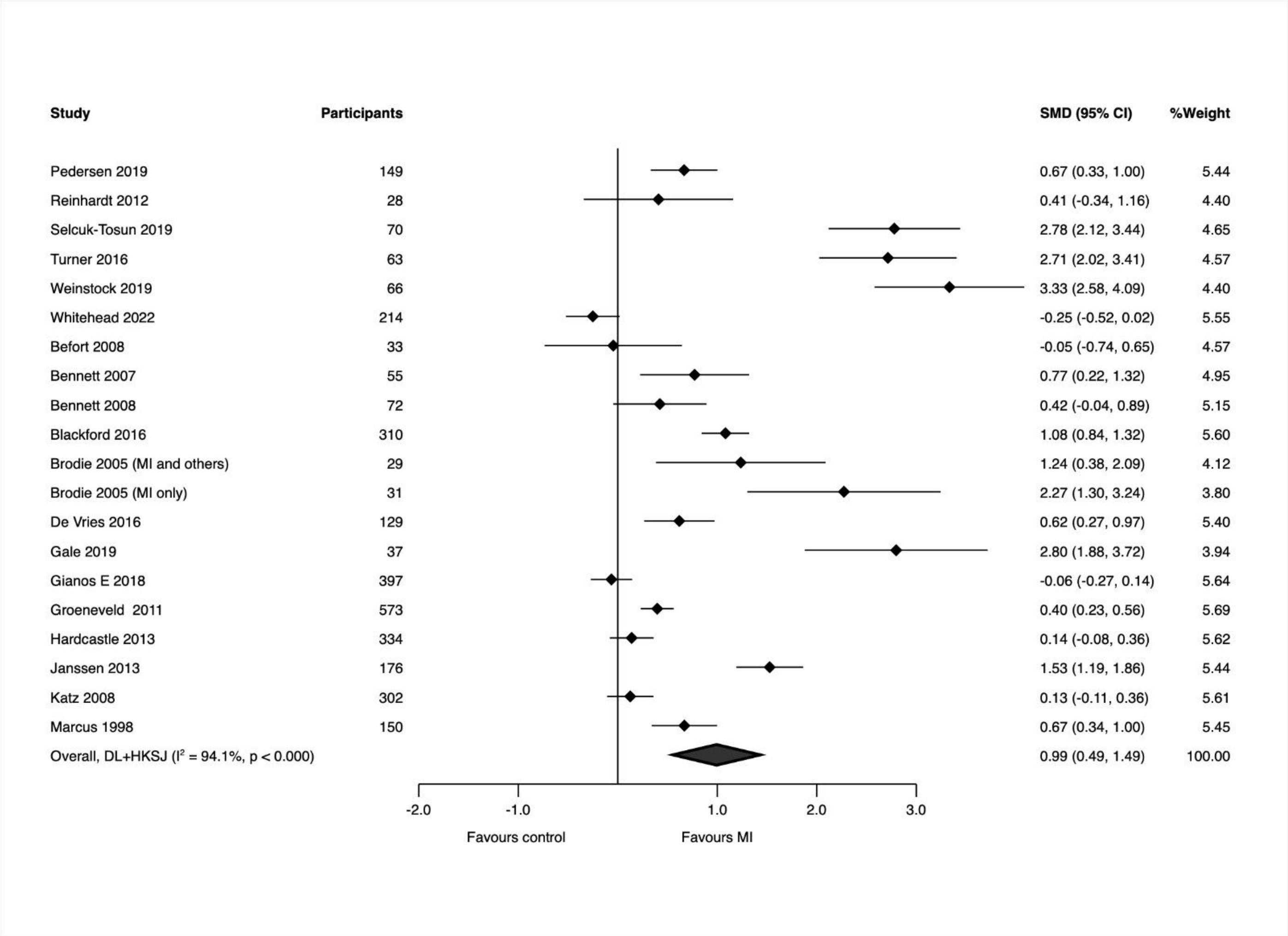

Supplementary Figure 30: Forest plot of studies with interventions 4-6 months duration reporting total physical activity outcomes at 7-12 months follow-up

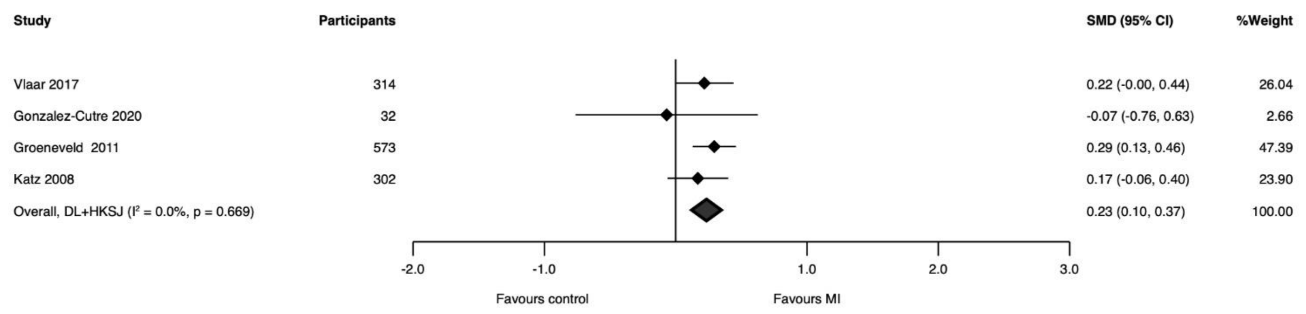

Supplementary Figure 31: Forest plot of studies with interventions 4-6 months duration reporting total physical activity outcomes at >1 year follow-up

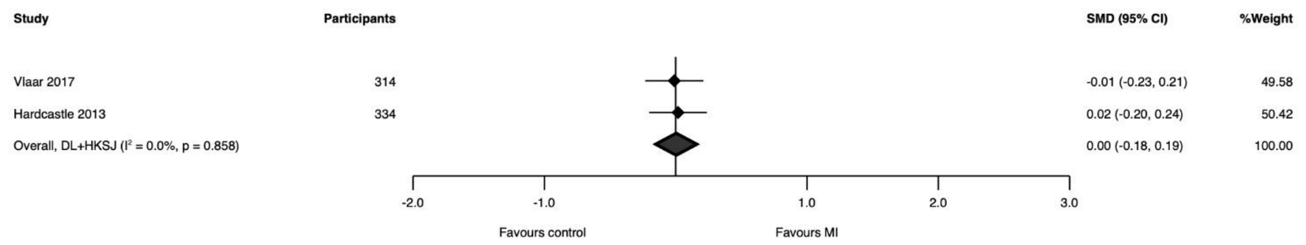

Supplementary Figure 32: Forest plot of studies with interventions 7-12 months duration reporting total physical activity outcomes at 7-12 months follow-up

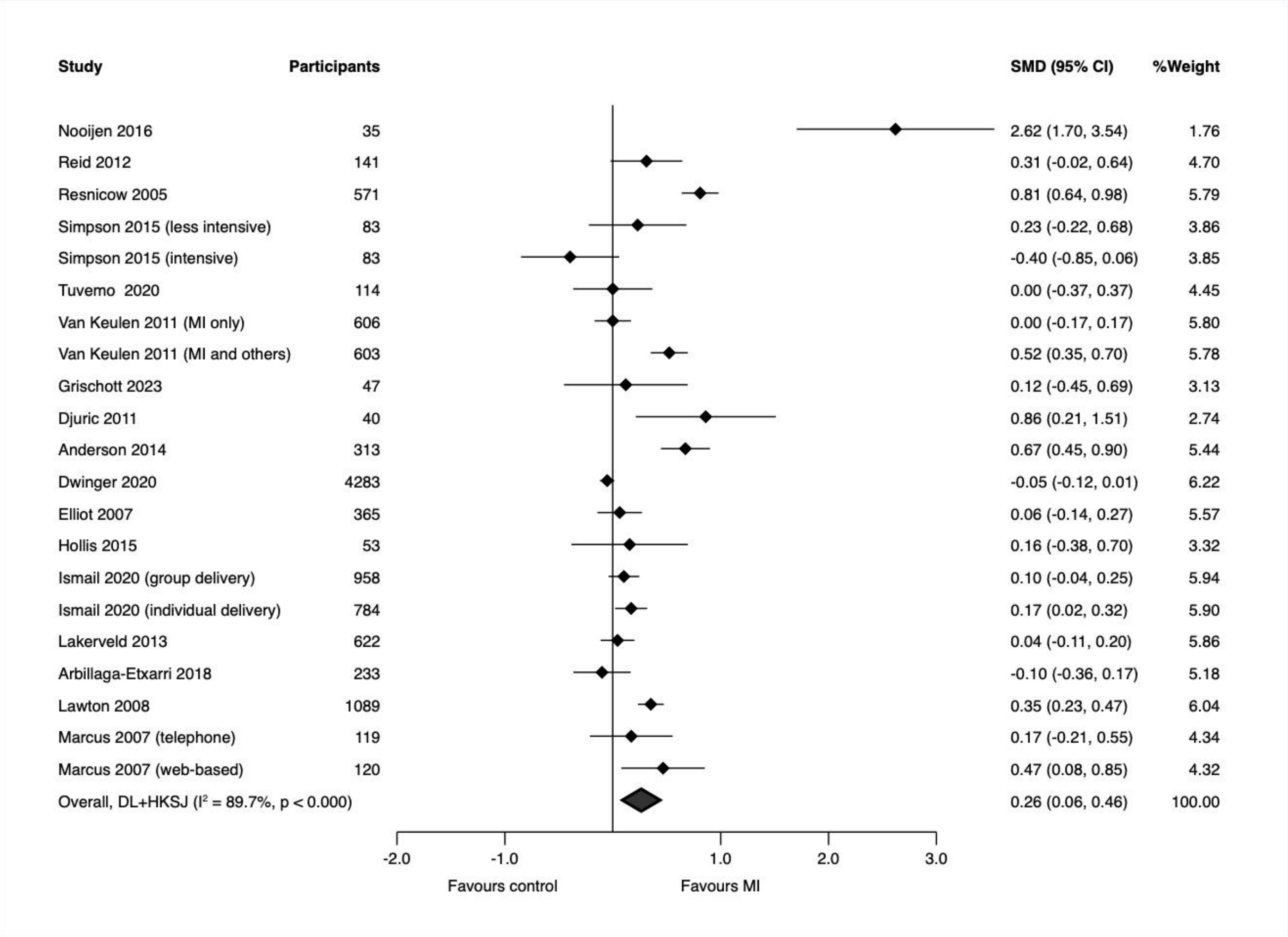

Supplementary Figure 33: Forest plot of studies with interventions 7-12 months duration reporting total physical activity outcomes at >1 year follow-up

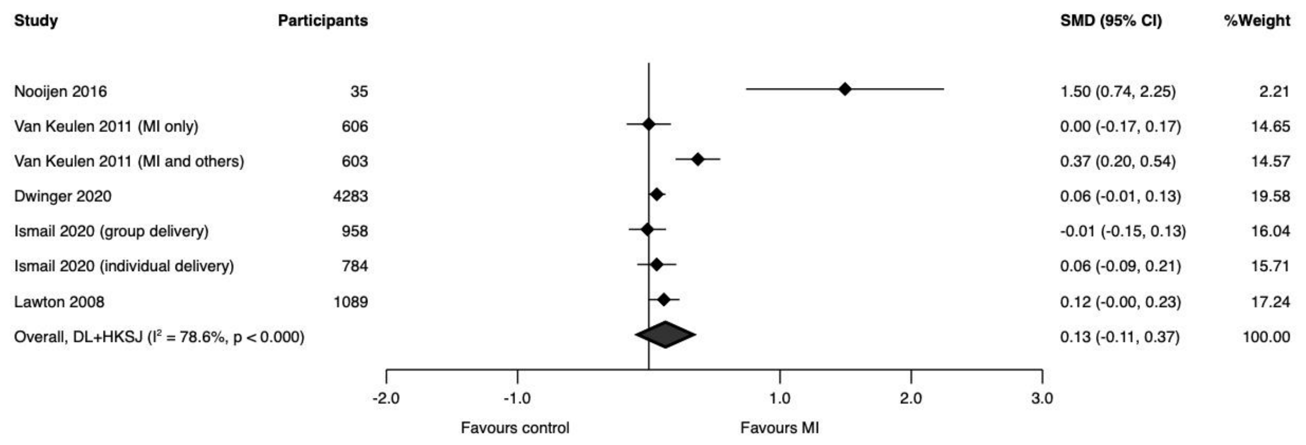

Supplementary Figure 34: Forest plot of studies with interventions >1 year duration reporting total physical activity outcomes at >1 year

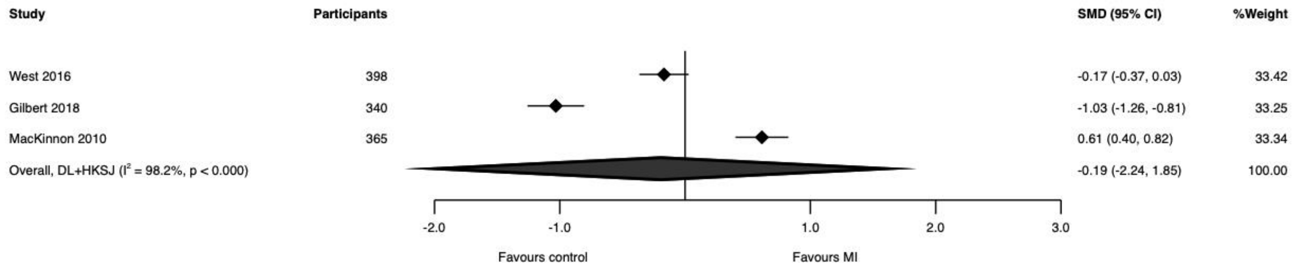

Supplementary Figure 35: Forest plot of studies with interventions reporting MVPA outcomes at 0-3 months follow-up by intervention duration

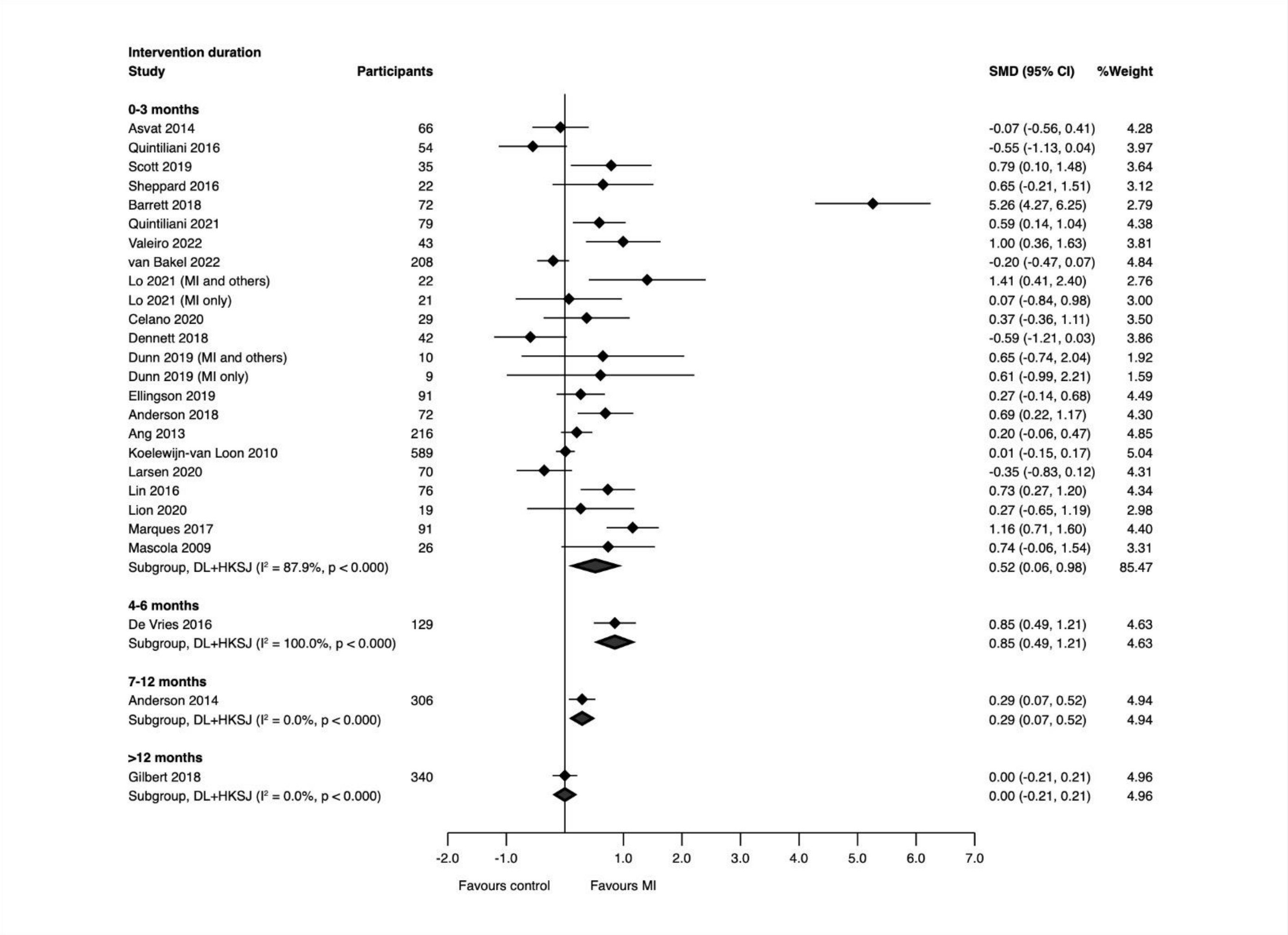

Supplementary Figure 36: Forest plot of studies with interventions reporting MVPA outcomes at 4-6 months follow-up by intervention duration

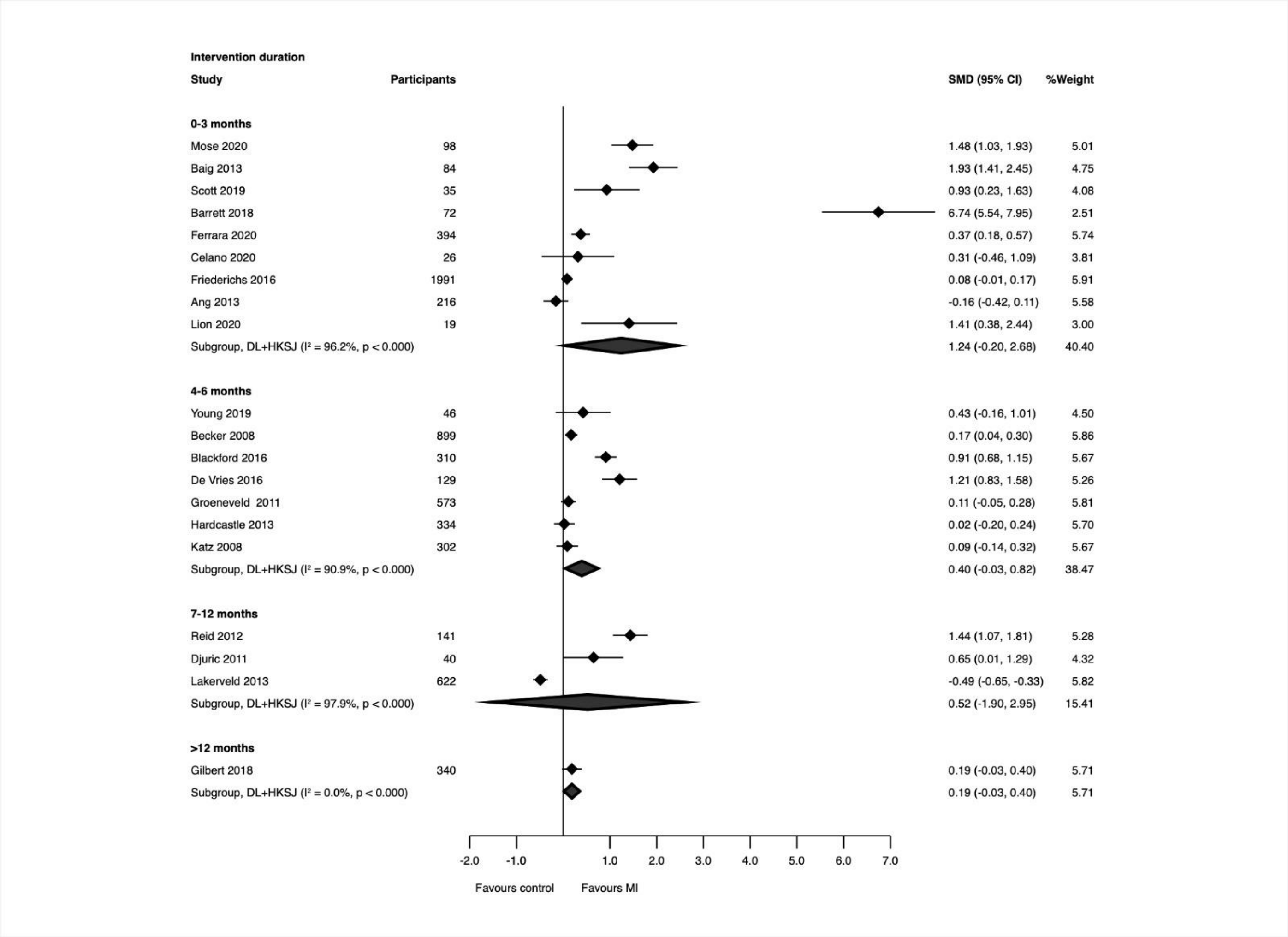

Supplementary Figure 37: Forest plot of studies with interventions reporting MVPA outcomes at 7-12 months follow-up by intervention duration

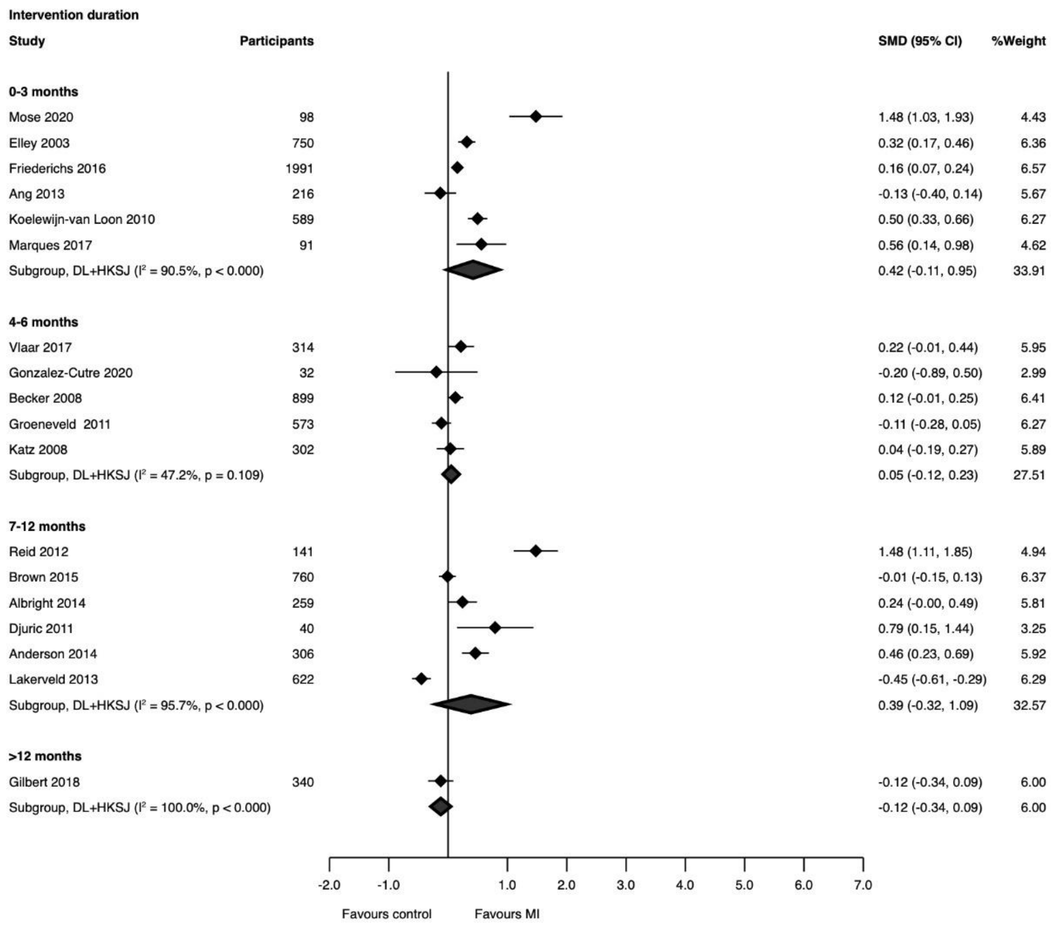

Supplementary Figure 38: Forest plot of studies reporting MVPA outcomes at >1 year follow-up by intervention duration

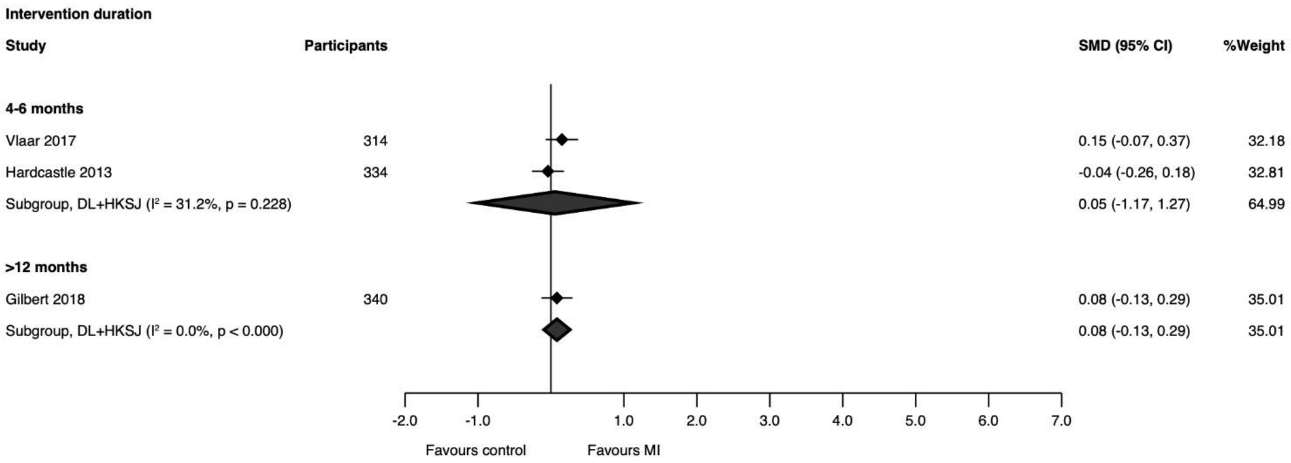

Supplementary Figure 39: Forest plot of studies with interventions reporting sedentary time outcomes at 0-3 months follow-up by intervention duration.

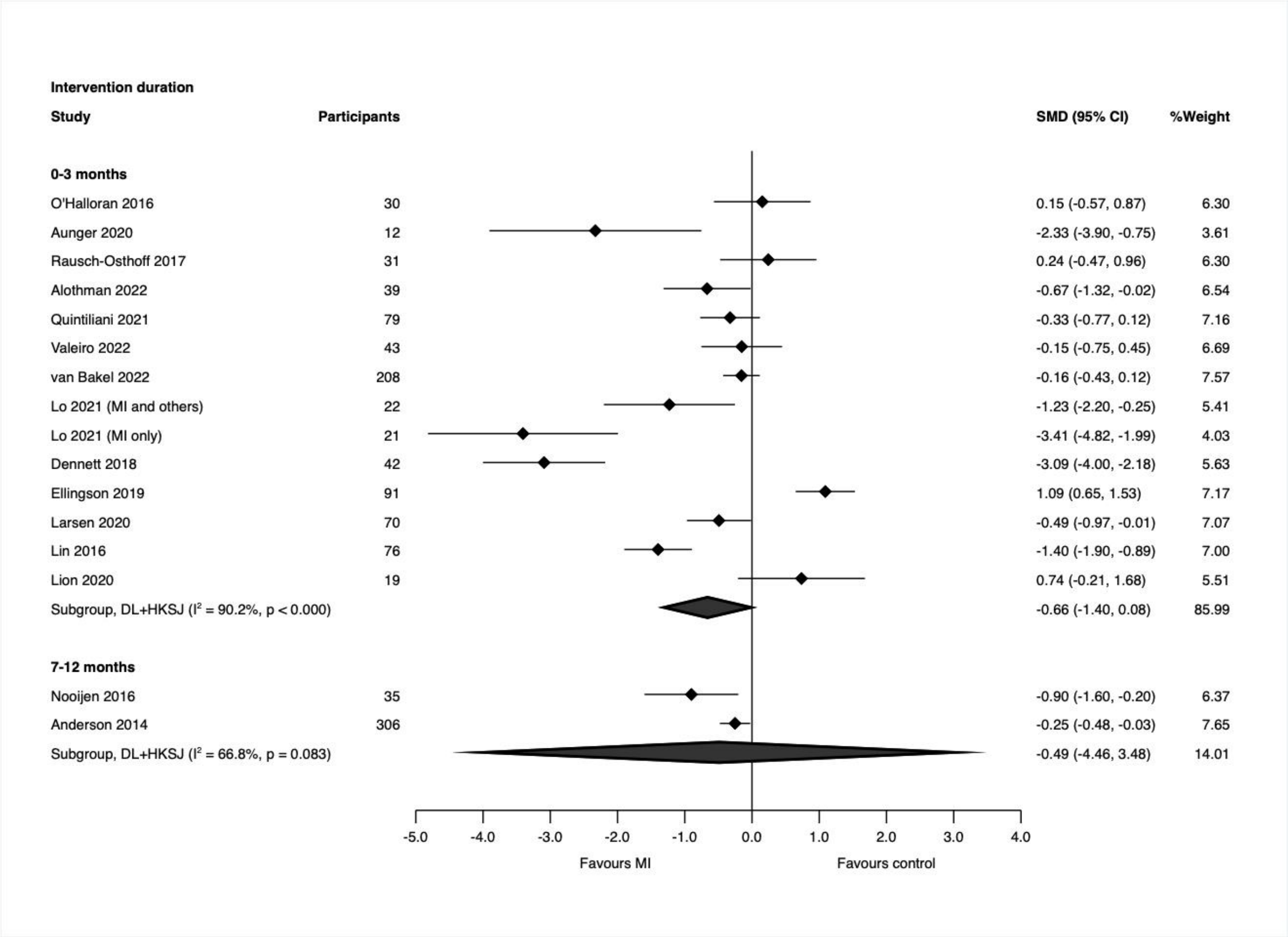

Supplementary Figure 40: Forest plot of studies with interventions reporting sedentary time outcomes at 4-6 months follow-up by intervention duration.

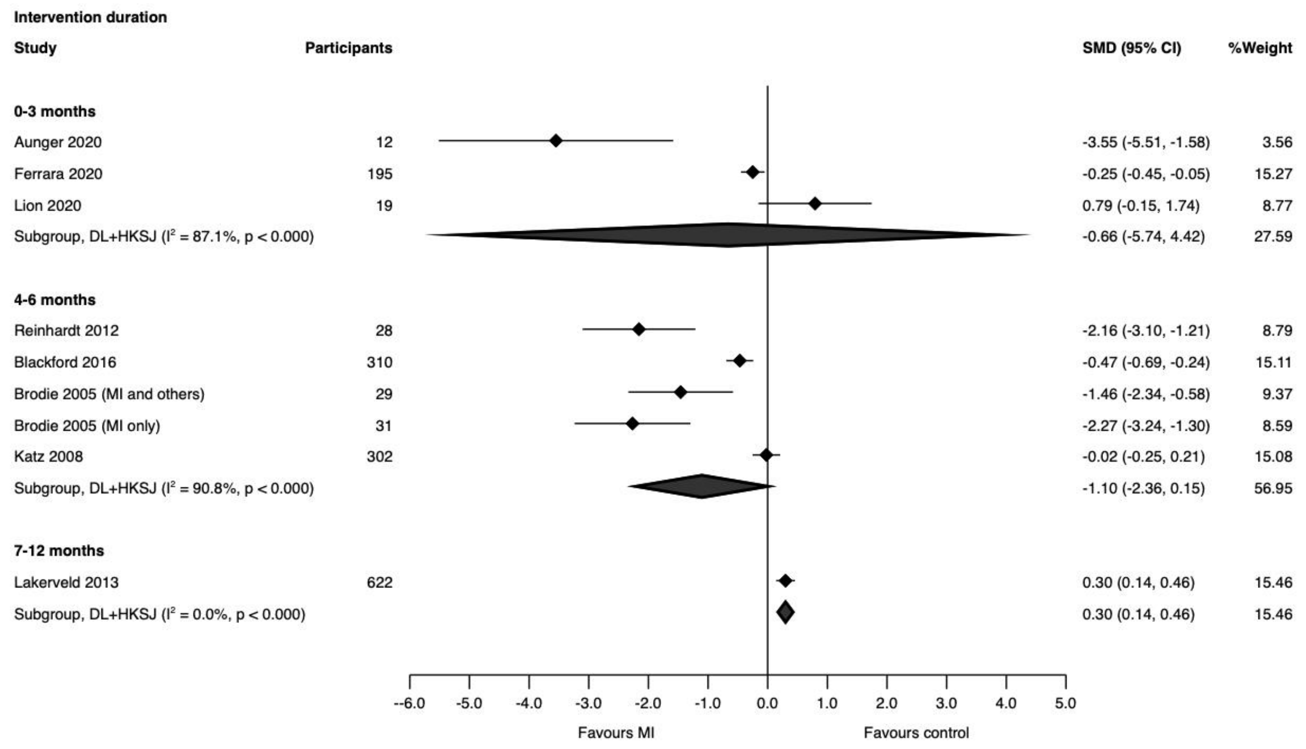

Supplementary Figure 41: Forest plot of studies with interventions reporting sedentary time outcomes at 7-12 months follow-up by intervention duration.

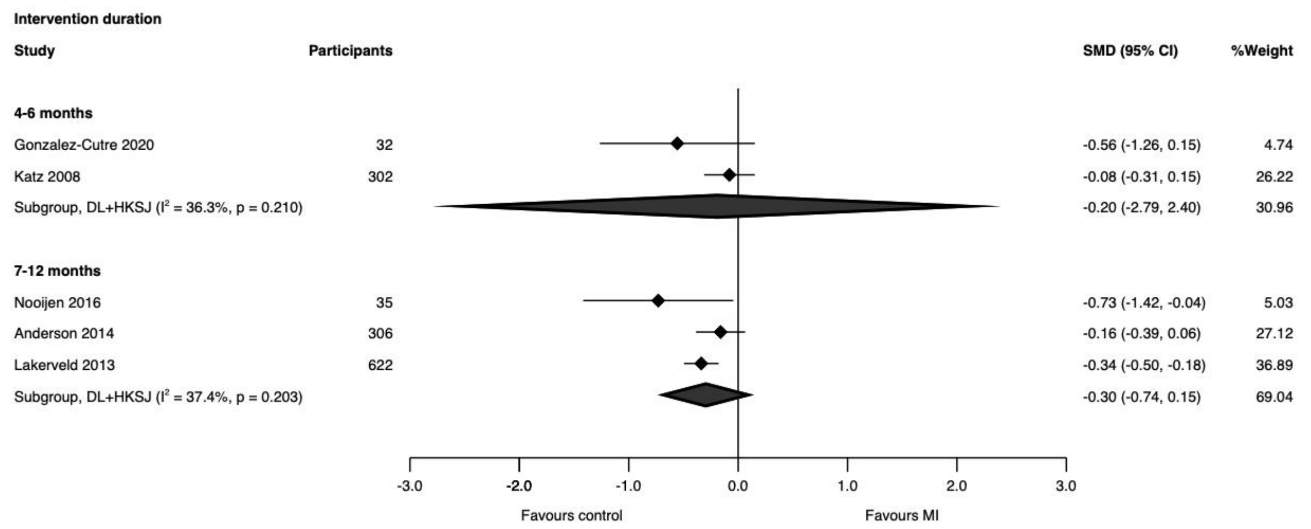

Supplementary Figure 42: Forest plot of studies with interventions reporting sedentary time outcomes at >1 year follow-up by intervention duration.

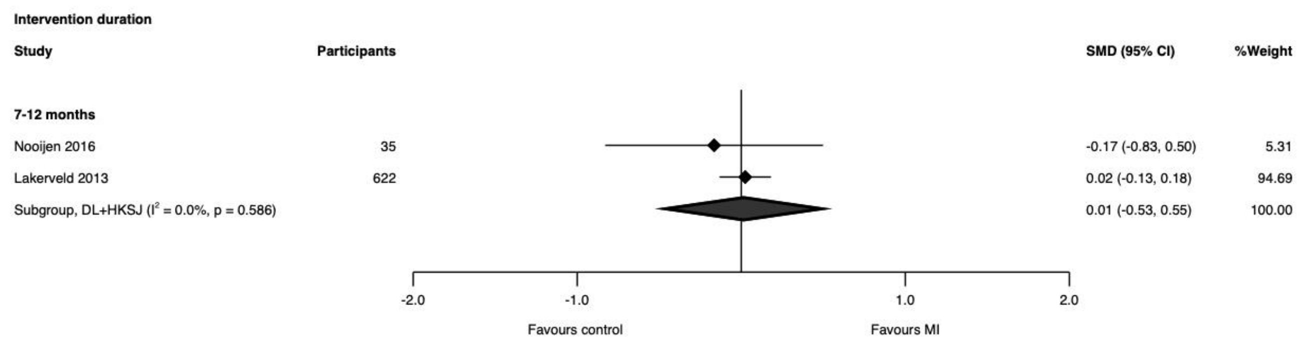

Supplementary Figure 43: Total PA outcomes in studies conducted in people with pre-existing disease or health condition

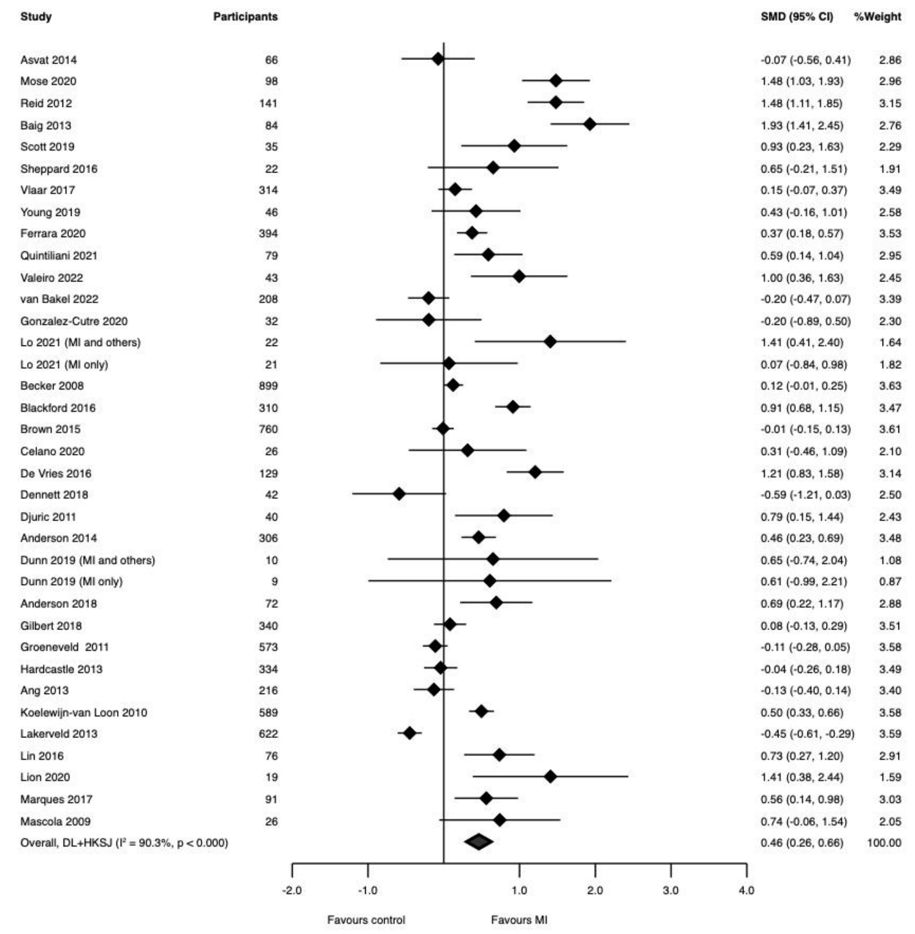

Supplementary Figure 44: MVPA outcomes in studies conducted in people with pre-existing disease or health condition

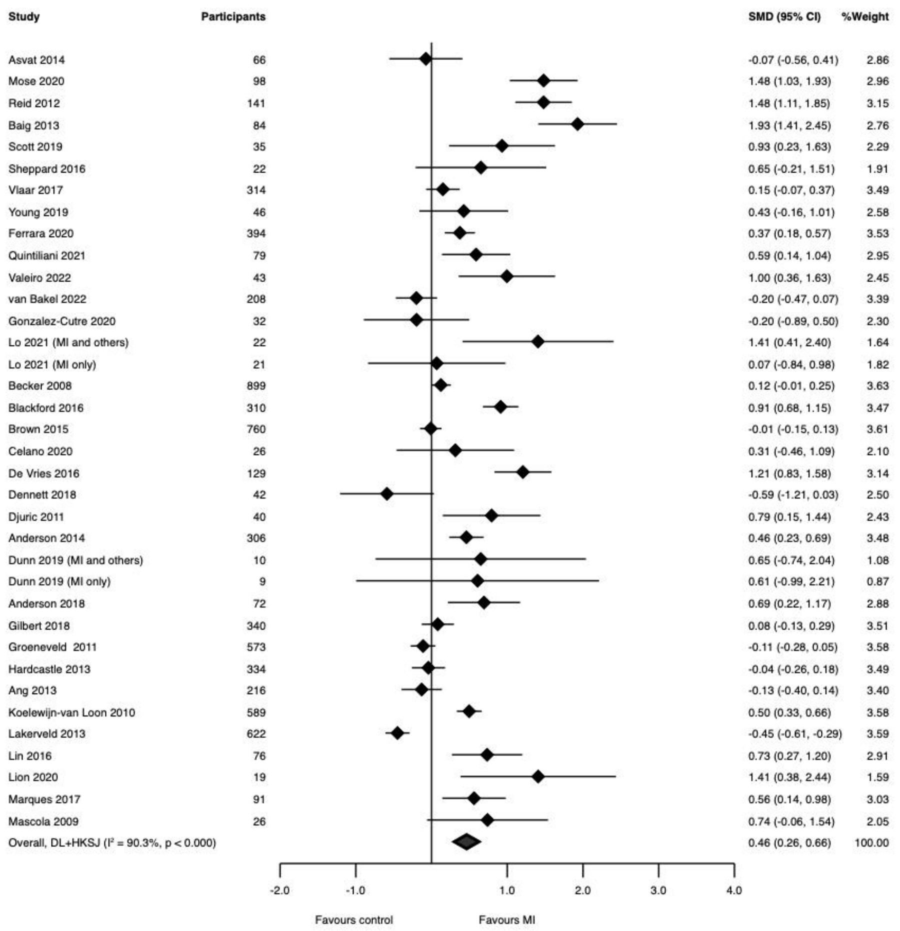

Supplementary Figure 45: Sedentary time outcomes in studies conducted in people with pre-existing disease or health condition

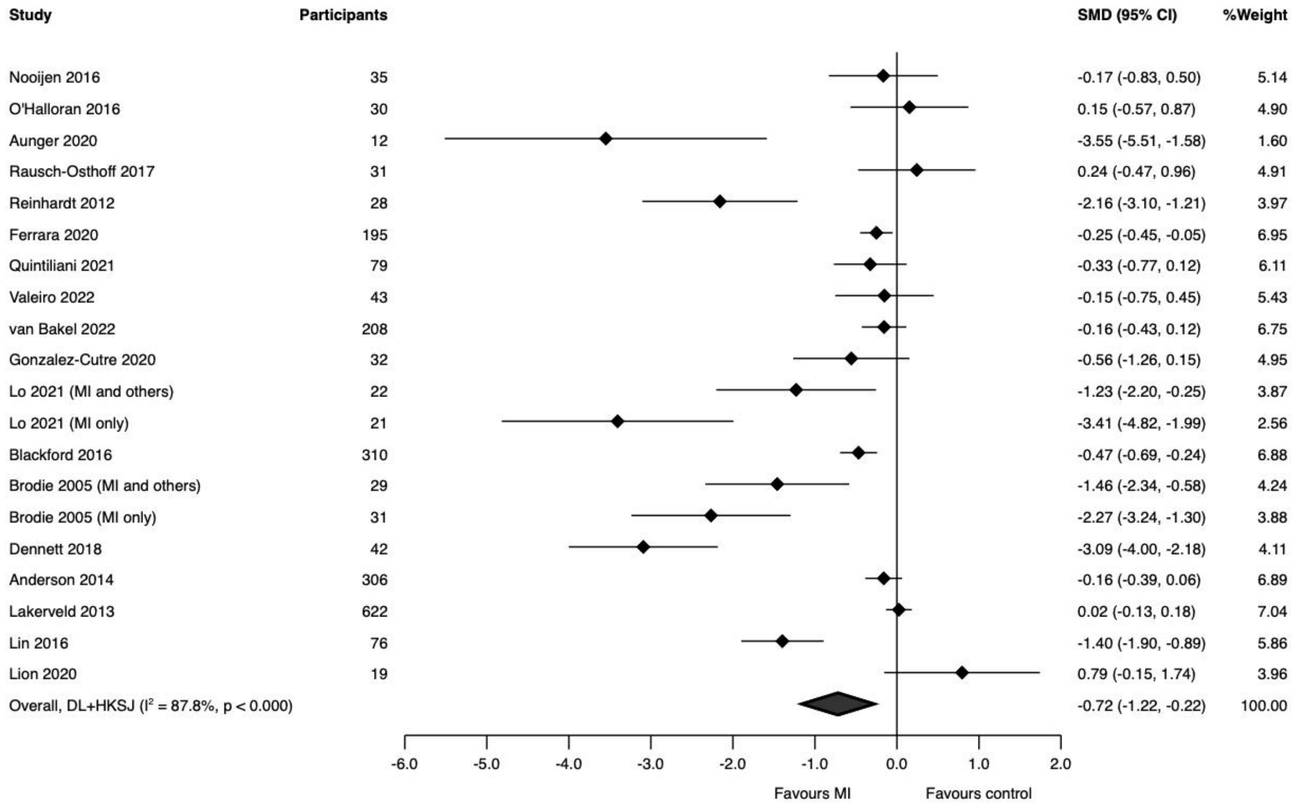

Supplementary Figure 46: Total PA outcomes in studies conducted in healthy people

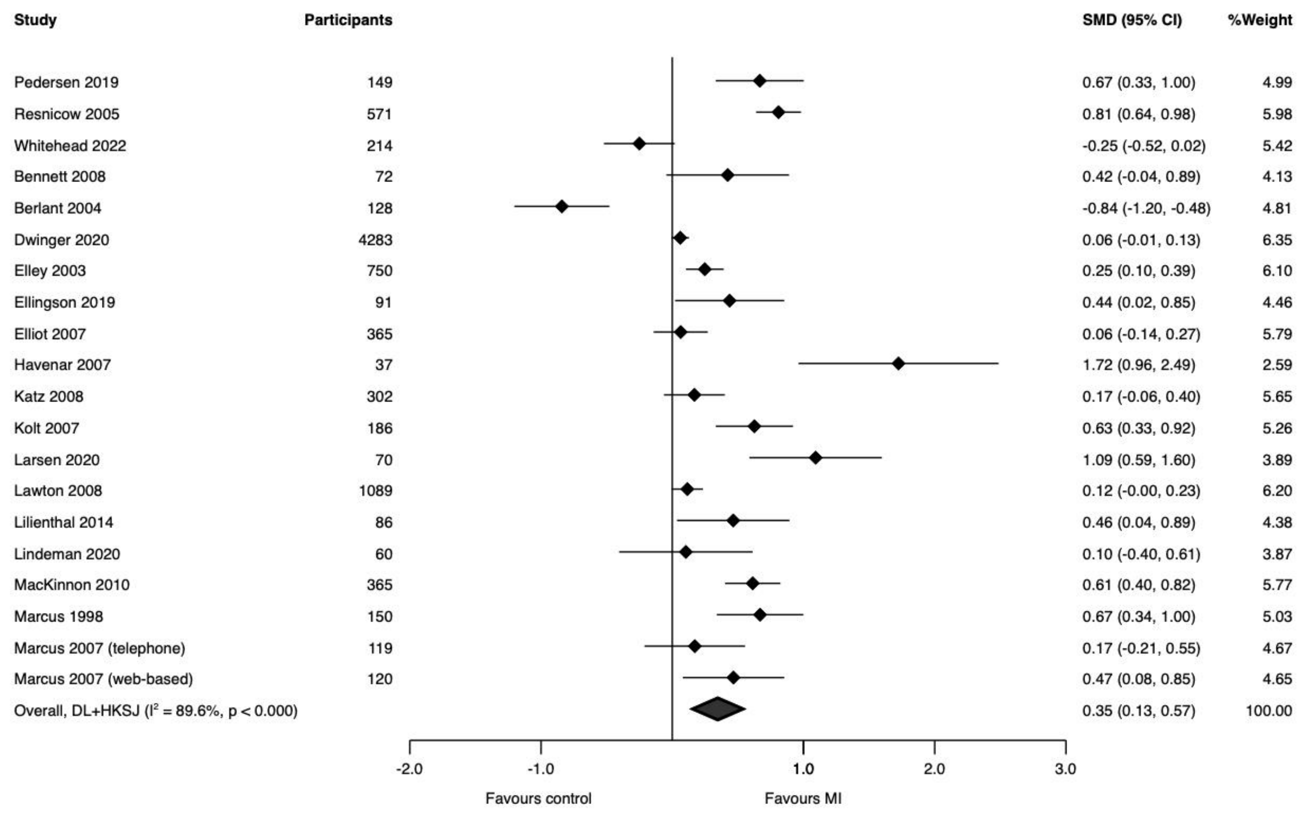

Supplementary Figure 47: MVPA outcomes in studies conducted in healthy people

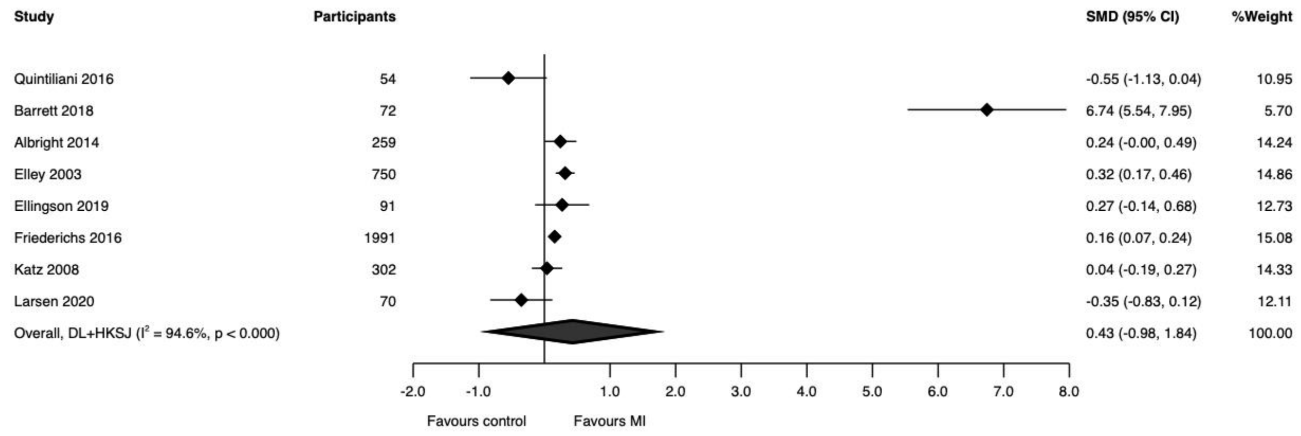

Supplementary Figure 48: Sedentary time outcomes in studies conducted in healthy people

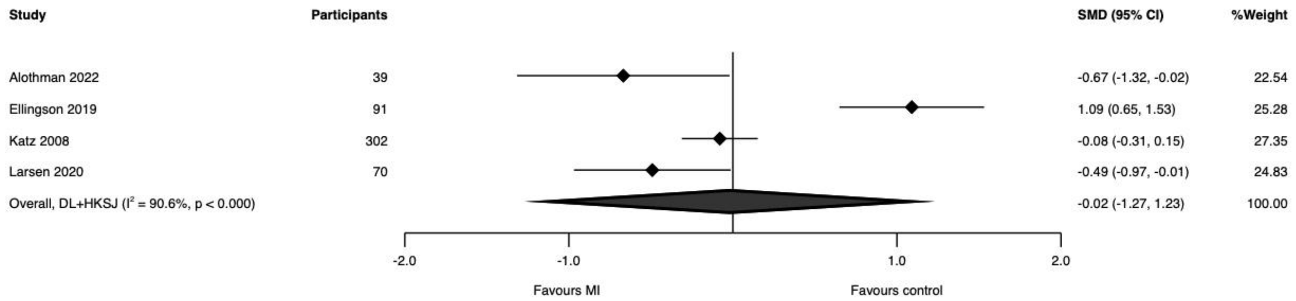

Supplementary Figure 49: Sensitivity analysis of studies reporting total PA outcomes excluding studies judged to be at overall high risk of bias

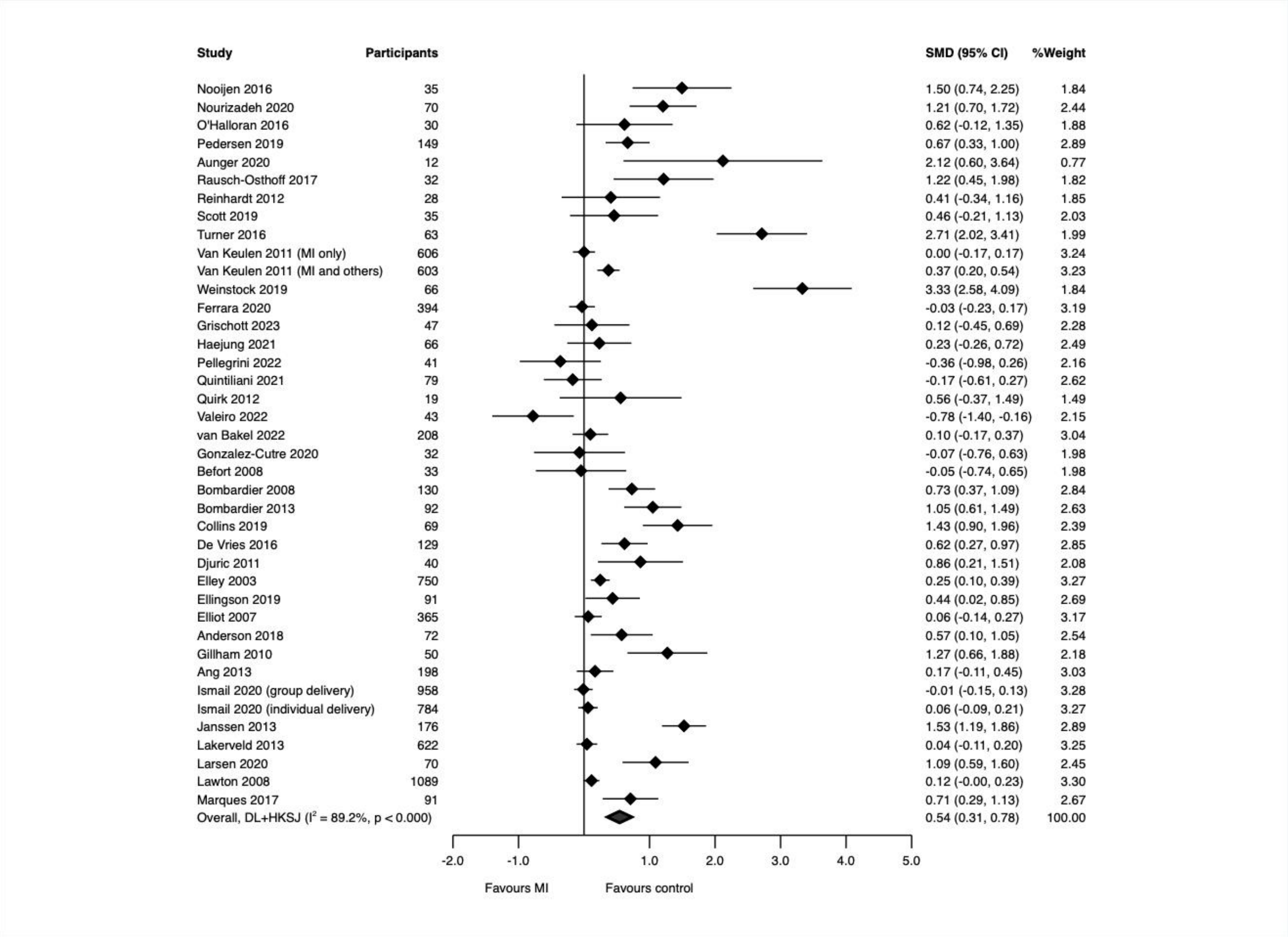

Supplementary Figure 50: Sensitivity analysis of studies reporting MVPA outcomes excluding studies judged to be at overall high risk of bias

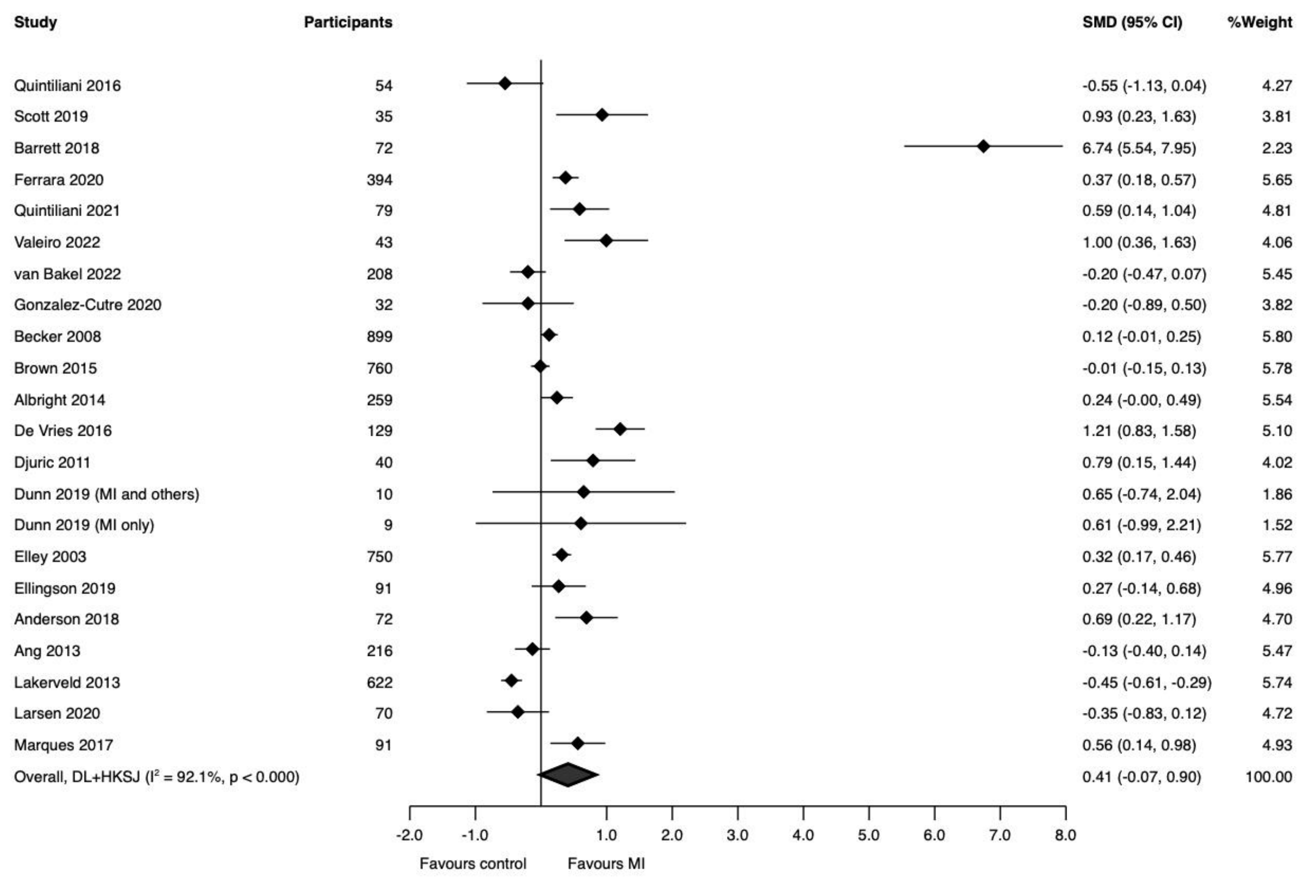

Supplementary Figure 51: Sensitivity analysis of studies reporting sedentary time outcomes excluding studies judged to be at overall high risk of bias

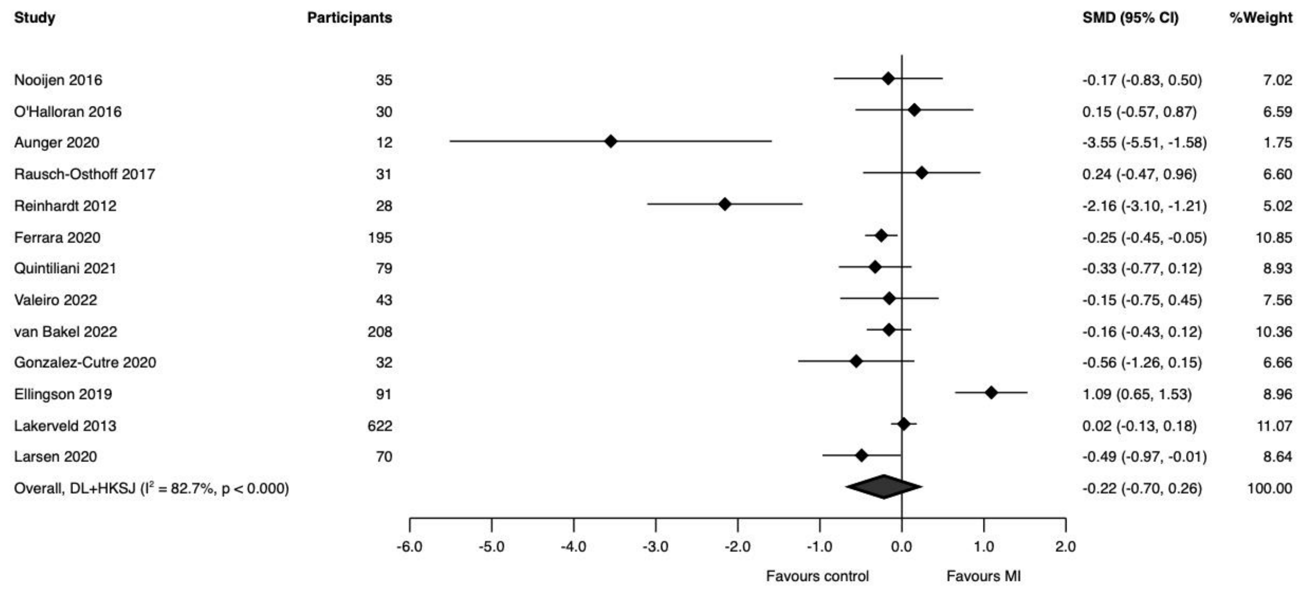

Supplement: Supplementary file 1 — Supplementary information: Additional tables 1-3 and figures 1-51 [file zhus078713.ww.pdf]
